# Efficacy and Safety of Pharmacological, Endoscopic, and Surgical Treatments for Obesity: A GRADE‐Based Network Meta‐Analysis

**DOI:** 10.1002/oby.70083

**Published:** 2026-01-15

**Authors:** Maurizio De Luca, Ricardo V. Cohen, Amanda Belluzzi, Giuseppe Navarra, Nicola Di Lorenzo, Tarissa B. Z. Petry, Paolo Sbraccia, Luca Busetto, Silvio Buscemi, Rocco Barazzoni, Benedetta Ragghianti, Edoardo Mannucci, Matteo Monami

**Affiliations:** ^1^ Department of General Surgery Rovigo Hospital Rovigo Italy; ^2^ Center for Obesity and Diabetes, Oswaldo Cruz German Hospital São Paulo Brazil; ^3^ University of Messina Messina Italy; ^4^ University La Sapienza Rome Italy; ^5^ University Hospital Policlinico Tor Vergata Rome Italy; ^6^ University of Padua Padua Italy; ^7^ University Hospital Policlinico “P. Giaccone,” Palermo Italy; ^8^ University Hospital Trieste Italy; ^9^ Unit of Diabetology and Metabolic Diseases. Careegi Teaching Hospital University of Florence Florence Italy

**Keywords:** endoscopic bariatric procedures, intervention, metabolic bariatric surgery, network meta‐analysis, no therapy, obesity, obesity management medications, placebo

## Abstract

**Objective:**

This review compared antiobesity strategies—obesity management medications (OMM), endoscopic bariatric procedures (EBP), and metabolic bariatric surgery (MBS)—with lifestyle intervention, placebo, or no therapy (LSI/Pbo/NT).

**Methods:**

This network meta‐analysis included randomized clinical trials comparing OMM, EBP, and MBS versus LSI/Pbo/NT or active comparators in adults with obesity. MEDLINE and Embase were searched up to December 1, 2024. The primary endpoint was total body weight loss percentage (TBWL%), analyzed at 26–52, 53–104, 105–156, and ≥ 156 weeks. This study was registered with PROSPERO (CRD42024623707).

**Results:**

Of 139 RCTs, 54 evaluated MBS (*n* = 61,961), 21 EBP (*n* = 2934), and 64 OMM (*n* = 5991). At 26–52 weeks, most treatments showed significant effects versus the reference. TBWL% exceeded 10% with most surgeries and tirzepatide. Long‐term data were lacking for most OMM and all EBP. Most treatments maintained their efficacy over time, except greater curvature plication. EBP and MBS were generally associated with a higher SAE risk than OMM; BPD showed the highest long‐term SAE incidence.

**Conclusions:**

MBS appears superior in the long term (particularly for higher‐efficacy procedures, such as RYGB, SG, SADI, and BPD). EBP, except ESG, was less effective than newer OMM. Semaglutide and tirzepatide showed no inferior short‐term results in comparison with MBS.

**Study Importance**

- What is already known?
  ○ Obesity has emerged as a global health crisis and is associated with numerous chronic diseases. The development of pharmacological, endoscopic, and surgical research has led to the discovery of novel treatments in recent years, providing clinicians with a relatively wide range of therapeutic options. However, the availability of randomized controlled trials directly comparing different pharmacologic approaches, other than lifestyle interventions, is still limited.
- What does this review add?
  ○ This network meta‐analysis (NMA), providing indirect comparisons of efficacy and safety, can be of help in guiding physicians’ choices. Several NMA studies on different therapeutic options for obesity were recently published; however, they were limited to either MBS or OMMs. Moreover, NMA studies on OMMs did not include the most recently approved agent, tirzepatide.
- How might these results change the direction of research or the focus of clinical practice?
  ○ This NMA enables health care practitioners and professionals involved in obesity management to have a complete picture of the efficacy and safety of available European Medicines Agency and Food and Drug Administration–approved treatment options, with data from the highest level of evidence during the selection process.

## 1 Introduction

Obesity is a growing public health issue worldwide because of its increasing prevalence and impact on metabolic and cardiovascular diseases, cancer, and overall mortality [1]. The treatment of obesity, which requires a long‐term modification of energy balance [2], represents a complex challenge for all health care professionals involved in this field. Unless combined with other treatments, lifestyle interventions (LSIs) have limited long‐term efficacy [3, 4] and require relevant health care resources [5]. Current guidelines suggest that, at least in a fraction of people with obesity, LSIs (i.e., structured dietary approaches and structured prescription of physical exercise) can be combined with either obesity management medications (OMMs) or metabolic bariatric surgery (MBS) [6, 7, 8, 9].

The development of pharmacological, endoscopic, and surgical research has led to the discovery of novel treatments in recent years, providing clinicians with a relatively wide range of therapeutic options [10]. However, the availability of randomized controlled trials (RCTs) directly comparing different OMMs, other than LSIs, is still limited. In particular, there are very few studies comparing OMMs with MBS. A network meta‐analysis (NMA), providing indirect comparisons of efficacy and safety, can therefore be of help in guiding physicians' choices. Several NMA studies on different therapeutic options for obesity were recently published; however, they were limited to either MBS [11] or OMMs [12]. Moreover, NMA studies on OMMs did not include the most recently approved agent, tirzepatide [12, 13], whereas one of them [12] analyzed molecules that have never been approved by regulatory authorities for treating obesity, being marketed for other indications.

The main objective of the following NMA is to provide health care practitioners and professionals involved in obesity management with a complete picture of the efficacy and safety of available European Medicines Agency (EMA) and Food and Drug Administration (FDA)–approved treatment options, with data from the highest level of evidence during the selection process.

## 2 Methods

The meta‐analysis has been reported following the criteria of the Preferred Reporting Items for Systematic Reviews and Meta‐Analyses (PRISMA) statement [14] (Table S1).

### 2.1 Search Strategy and Selection Criteria

The protocol of the present meta‐analysis and NMA was published on the PROSPERO website (https://www.crd.york.ac.uk/prospero/#recordDetails, registration number: CRD42024623707). NMA is a statistical approach that simultaneously compares multiple treatments by integrating direct and indirect evidence across a network of studies. It enables the ranking of interventions and often yields more precise estimates than traditional pairwise meta‐analyses. The present analysis included all RCTs enrolling patients with obesity (body mass index [BMI] ≥ 27 kg/m^2^), comparing different either EMA‐approved OMMs and/or FDA‐approved OMMs and surgical interventions (MBS and endoscopic bariatric procedures [EBP]) versus LSI/placebo or no intervention or comparing two different active treatments. To be included in the analyses, RCTs should have a minimum follow‐up (for MBS)/treatment (for OMMs) of 52 weeks, except for EBP, for which a follow‐up/treatment period of 6 months was considered. A MEDLINE and Embase search was performed up to December 1, 2024. The search was conducted using a combination of subject terms and free terms. The subject terms included “obesity or overweight” AND “orlistat OR phentermine OR topiramate OR naltrexone OR bupropion OR liraglutide OR semaglutide OR tirzepatide OR sleeve gastrectomy OR Roux‐en‐Y gastric bypass OR one‐anastomosis gastric bypass OR laparoscopic adjustable gastric banding OR biliopancreatic diversion OR single anastomosis duodenal‐ileal bypass OR intragastric balloon OR primary obesity surgery endoluminal OR endoscopic sleeve gastroplasty” and “randomized controlled trial.” References in other relevant articles and gray literature were manually searched to pinpoint studies that met the criteria. Detailed information on the search strategy and keywords used is reported in Table S2. Animal studies were excluded, whereas no language or date restriction was imposed.

Duplicate records were removed with EndNote X9 (Clarivate Analytics). Teams of paired reviewers independently used EndNote X9 to screen titles and abstracts, then full‐text manuscripts, and extracted data on studies fulfilling inclusion and exclusion criteria.

### 2.2 Interventions Assessed

*OMMs*: Orlistat (360 mg), phentermine (15 mg), ephedrine plus caffeine (75/150 mg), phentermine plus topiramate (PT, 15/92 mg), naltrexone plus bupropion (NB, 32/360 mg), liraglutide (3.0 mg), semaglutide (2.4 mg), and tirzepatide (10–15 mg) versus placebo/none or active comparators.

*MBS*: Sleeve gastrectomy (SG), Roux‐en‐Y gastric bypass (RYGB), one‐anastomosis gastric bypass (OAGB), laparoscopic adjustable gastric banding (LAGB), biliopancreatic diversion (BPD), single anastomosis duodenal‐ileal bypass (SADI), and greater curvature plication (GCP) versus placebo/none or active comparators.

*EBP*: Intragastric balloon (IGB), primary obesity surgery endoluminal (POSE), endoscopic sleeve astroplasty (ESG), and aspiration therapy (AT) versus placebo/none or active comparators.

### 2.3 Data Extraction

Information on the baseline characteristics of the samples enrolled (age, gender, proportion of people with diabetes, baseline BMI), total body weight loss (TBWL%), quality of life (QoL), serious adverse events (SAE), and all‐cause mortality were extracted from the principal publication, when available (Table S3 reports a detailed list). The weight loss outcome was reported in TBWL%, = [(weight at the time of study inclusion) − (postop weight)]/[(weight at the time of study inclusion)] 100.

Whenever needed, secondary publications and the ClinicalTrials.gov registry were used to retrieve missing information in the hierarchical order reported earlier. Clinically meaningful weight loss was defined as achieving ≥ 10% TBWL from baseline. For each trial, TBWL% was extracted at 52 weeks and at the last available time point between 53 and 104 weeks, 105 and 156 weeks, and after 156 weeks. Two authors performed data extraction independently (B.R., A.B.), and conflicts were resolved by a third investigator (M.M.). Both intention‐to‐treat (ITT) and per‐protocol (PP) analyses were performed.

The risk of bias (RoB) was assessed using the Cochrane recommended tool to determine the RoB in RCTs [15]. The RoB was described and evaluated in seven specific domains: random sequence generation, allocation concealment, blinding of participants and personnel, blinding of outcome assessment, incomplete outcome data, selective reporting, and other biases. The results of these domains were graded as “low” RoB, “high” RoB, or “uncertain” RoB. Two researchers (A.B. and B.R.) independently assessed the RoB in individual studies, with discrepancies resolved by a third researcher (M.M.).

Random‐effects NMA was employed using a frequentist approach and a consistency model for benefit outcomes. Transitivity and coherence were assessed using meta‐regressions and global and local coherence tests. Statistical analyses were completed, including homogeneity tests, sensitivity analysis, transitivity tests, consistency tests, and publication bias. *H* values ≥ 3 are considered.

### 2.4 Data Analysis

The principal endpoint was TBWL%; secondary endpoints were QoL, SAE (an event is defined as serious when it results in death, is life‐threatening, requires inpatient hospitalization or extends a current hospital stay, results in an ongoing or significant incapacity or interferes substantially with normal life functions, or causes a congenital anomaly or birth defect; https://clinicaltrials.gov/study‐basics/glossary), and all‐cause mortality. The primary endpoint was analyzed at different time points: 26–52 (up to 1 year), 53–104 (1–2 years), 105–156 (2–3 years), and ≥ 156 (≥ 3 years) weeks. Secondary endpoints (usually reported at the end of the study) were analyzed separately in trials with a duration of 26–52 (up to 1 year), 53–104 (1–2 years), 105–156 (2–3 years), and ≥ 156 (≥ 3 years) weeks.

### 2.5 Statistical Analyses

Mean and 95% confidence intervals (95% CIs) for continuous variables and Mantel–Haenszel odds ratios (MH‐OR) for categorical variables were calculated using random‐effect models. When data were reported as least‐squares mean and standard error (SE), standard deviation (SD) was obtained for each group using the following formula: SD = √(number of patients) * (CI upper limit − CI lower limit)/3.92 and SD = √(number of patients) * SE, respectively (http://handbook‐5‐1.cochrane.org/chapter_7/7_7_3_2_obtaining_standard_deviations_from_standard_errors_and.htm).

Several prespecified subgroup analyses were performed for the following baseline variables: different types of antiobesity strategies (i.e., surgical and endoscopic procedures and OMMs), BMI categories (mean BMI at the enrollment < 30, 30–34.9, 35–39.9, and ≥ 40 kg/m^2^), and type 2 diabetes mellitus (T2DM; yes: RCTs enrolling at least 75% of patients with diabetes; no: RCTs enrolling no more than 25% of patients with T2DM). Traditional meta‐analyses were performed for all the placebo‐ and active‐controlled trial endpoints. Heterogeneity was assessed by using *I* ^2^ statistics. A random‐effects model was applied for all the analyses reported.

### 2.6 NMA Elements

NMA [16] was performed for all outcomes to verify differences across individual antiobesity strategies concerning their effects on primary and secondary endpoints. These analyses enable indirect comparisons when direct trials are unavailable, by utilizing differences from standard comparators and then combining direct and indirect comparisons to obtain a final effects estimate. The reference category was LSI/none/placebo (considered unique).

NMA was performed within a pairwise modeling (GPM) framework using three different software programs: Metainsight version 6.0.0 (https://crsu‐metainsight.le.ac.uk/MetaInsight/), MetaXL (www.epigear.com), and CINeMA (https://cinema.ispm.unibe.ch/#).

#### 2.6.1 Assessment Network Geometry

The graphical representation of the geometry of all networks of interventions was depicted using diagrams that allowed for the representation of whether information comparing each pair of interventions came from direct evidence (i.e., studies comparing two interventions head‐to‐head against one another), indirect evidence (i.e., studies comparing two interventions through a common comparator, called reference category), or both (combination of direct and indirect evidence for estimating the relative effect of pairs of interventions across a network of interventions). All diagrams were composed of nodes (i.e., circles representing each intervention included in NMA) and links (i.e., lines connecting two nodes). A link between two nodes indicates that there is direct evidence for the comparison. Node size and edge thickness, as well as colors, were used to represent different characteristics of the network, including the number of studies comparing two interventions, the number of participants in each comparison, and the RoB. Multiarm studies (i.e., primary studies with three or more arms comparing different interventions) were reported for the primary endpoint.

#### 2.6.2 Assessment of Transitivity

When direct comparisons (i.e., no head‐to‐head comparisons) are not available between two different interventions (A and B), but each of those interventions has been compared against a common intervention (i.e., A and B have been directly compared to C), the indirect comparison is reliable and unbiased only if the study characteristics (modifiers) of the direct comparisons are not significantly different between the two direct comparisons (i.e., A vs. C and B vs. C). We decided to use any type of “control group” (i.e., either placebo, LSI, or no intervention: “none”) as the reference category for all analyses. Due to possible transitivity concerns, we explored several possible modifiers (i.e., baseline mean age, BMI, proportion of women, and participants with diabetes) across the three different types of control groups. In case of statistically significantly different study characteristics across different comparisons, whenever possible, post hoc subgroup analyses were performed considering placebo as the reference category.

#### 2.6.3 Consistency Assessment

The level of statistical agreement between direct and indirect evidence was assessed for the principal outcome to verify that differences between direct and indirect estimates (used to calculate NMA estimates) were trivial. Inconsistency was tested within each comparison and with the notesplitting model for all studies (Metainsight). *H* values were also calculated to test consistency between direct and indirect evidence; an *H* value <3 indicates minimal inconsistency in treatment effects (MetaXL).

#### 2.6.4 Risk of Bias

The Risk of Bias in Network Meta‐Analysis (RoB NMA) tool was adopted to assess the RoB of each included comparison (CINeMA). Whenever possible, we conducted sensitivity post hoc analyses, excluding high‐risk studies, to demonstrate the robustness of NMA findings.

All other analyses were performed using Review Manager (RevMan), version 5.3 (Copenhagen: The Nordic Cochrane Centre, The Cochrane Collaboration, 2014) and SPSS version 25.0 (IBM, SPSS Inc.).

GRADE methodology [7] was used to assess the quality of the body of retrieved evidence (individual studies) for the principal endpoint, using the GRADEpro GDT software (GRADEpro Guideline Development Tool, McMaster University, 2015, available from https://www.gradepro.org/).

## 3 Results

### 3.1 Retrieved Trials

The trial flow summary is reported in Figure S1. The search of MEDLINE and Embase databases allowed the identification of 3646 items. After excluding 1993 studies by reading the title and the abstract, 14 trials were excluded when reviewing the full text (Table S4). Of 139 trials fulfilling all inclusion criteria, 54, 21, and 64 trials on MBS, EBP, and OMMs were compared with either LSI/no therapy/placebo or other active antiobesity strategies. Some trials reported multiple comparisons [15, 17, 18, 19, 20, 21]. Therefore, the number of available comparisons was 150. The numbers of patients enrolled were 61,961, 2934, and 5991 in trials with OMMs, EBP, and MBS, respectively (Table S5).

The main characteristics of the included trials are reported in Table S5. Only one study [22] reported a mean BMI at entry of < 30 kg/m^2^ and a further trial of subgroup analysis for the primary endpoint for different BMI categories [23].

The quality of studies was heterogeneous (Figure S2). All trials on surgical and endoscopic procedures, except seven (11%) [24, 25, 26, 27, 28, 29, 30], were open‐label. In many trials, the attrition rate and/or the description of allocation and blinding of assessors were inadequate (Figures S2 and S3). Trials on OMMs were more frequently double‐blind (66%), with fewer trials with inadequate attrition and/or description of allocation or blinding of assessors (29.3%).

### 3.2 Meta‐Analysis of Placebo‐Controlled Trials

**FIGURE 1:**
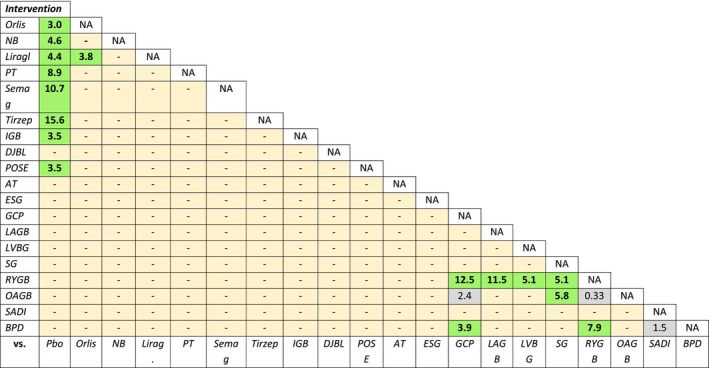
Effects of each antiobesity strategy on TBWL% at the endpoint in placebo‐ and active‐controlled trials. A positive value means an effect favoring the intervention in the first column. Significant results are reported in bold characters. For details, see Figure S4. AT, aspiration therapy; BPD, biliopancreatic diversion; DJBL, duodenal jejunal bypass liner; ESG, endoscopic sleeve gastroplasty; GCP, greater curvature plication gastric; IGB, intragastric balloon; LAGB, laparoscopic adjustable gastric banding; Lirag, liraglutide; LVGB, laparoscopic vertical banded gastroplasty; NA, not applicable; NB, naltrexone/bupropion; OAGB, one‐anastomosis gastric bypass; Orlis, orlistat; Pbo, placebo; POSE, primary obesity surgery endoluminal; PT, phentermine/topiramate; RYGB: Roux‐en‐Y gastric bypass; SADI, single anastomosis duodenal switch; Semag, semaglutide; SG, sleeve gastrectomy; Tirzep, tirzepatide. [Color figure can be viewed at wileyonlinelibrary.com]

Out of 59 placebo‐controlled trials, 52 reported information on TBWL% (*N* = 53,313 patients). No RCT was performed on MBS; only 2 trials with IGB [24, 30] and 1 with POSE [28] were placebo‐controlled studies. The remaining 49 RCTs were all performed with OMMs (Figure S4). Placebo‐subtracted effects for any antiobesity intervention included in the present meta‐analysis are reported in Figure 1. All investigated interventions determined a significant weight loss compared to placebo (Figure S4).

### 3.3 Meta‐Analysis of Direct Head‐to‐Head Comparisons

Only one head‐to‐head comparison between different OMMs was retrieved (i.e., liraglutide vs. orlistat [17]), showing a greater efficacy for liraglutide than for orlistat. Several trials compared different types of MBS, showing that the efficacy of RYGB is greater than that of GCP, LAGB, laparoscopic vertical banded gastroplasty (LVGB), and SG, similar to OAGB, and statistically inferior to BPD (Figure 1).

### 3.4 NMA: OMMs, MBS, and EBP Versus LSI/Placebo/No Therapy

**FIGURE 2:**
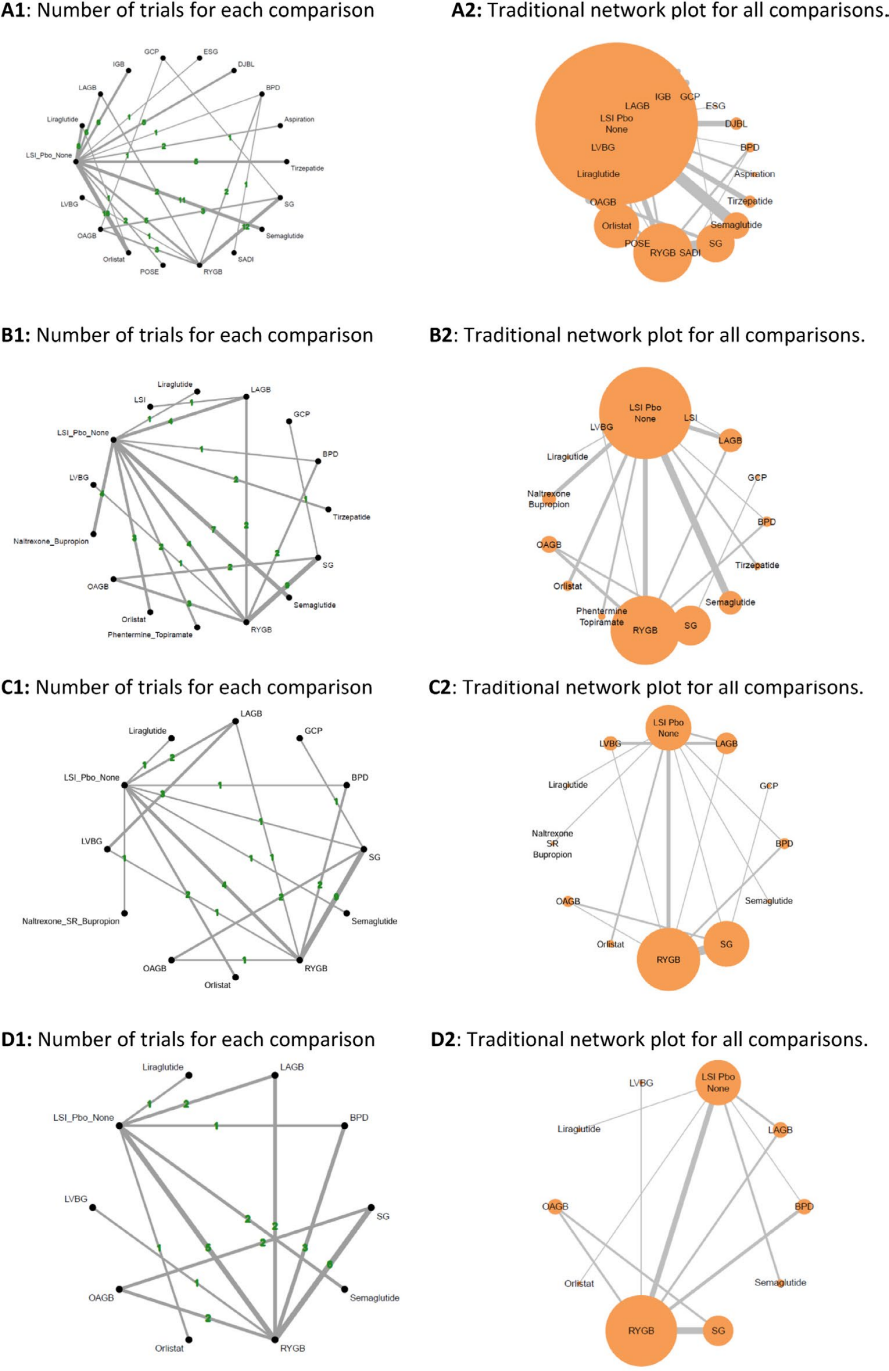
Network meta‐analysis for different antiobesity strategies. Network plots for TBWL% at (A) 26–52 weeks, (B) 53–104 weeks, (C) 105–156 weeks, and (D) 156–520 weeks. The size of the nodes represents the number of participants randomized to each node across studies. The thickness of the edges represents the number of studies comparing two interventions. Colors represent the quality of each comparison (red, yellow, and green: high, medium, and low risk of bias, respectively). BPD, biliopancreatic diversion; GCP, greater curvature plication gastric; LAGB, laparoscopic adjustable gastric banding; LSI, lifestyle intervention; LVGB, laparoscopic vertical banded gastroplasty; OAGB, one‐anastomosis gastric bypass; Pbo, placebo; RYGB, Roux‐en‐Y gastric bypass; SADI, single anastomosis duodenal switch; SG, sleeve gastrectomy. [Color figure can be viewed at wileyonlinelibrary.com]

**FIGURE 3:**
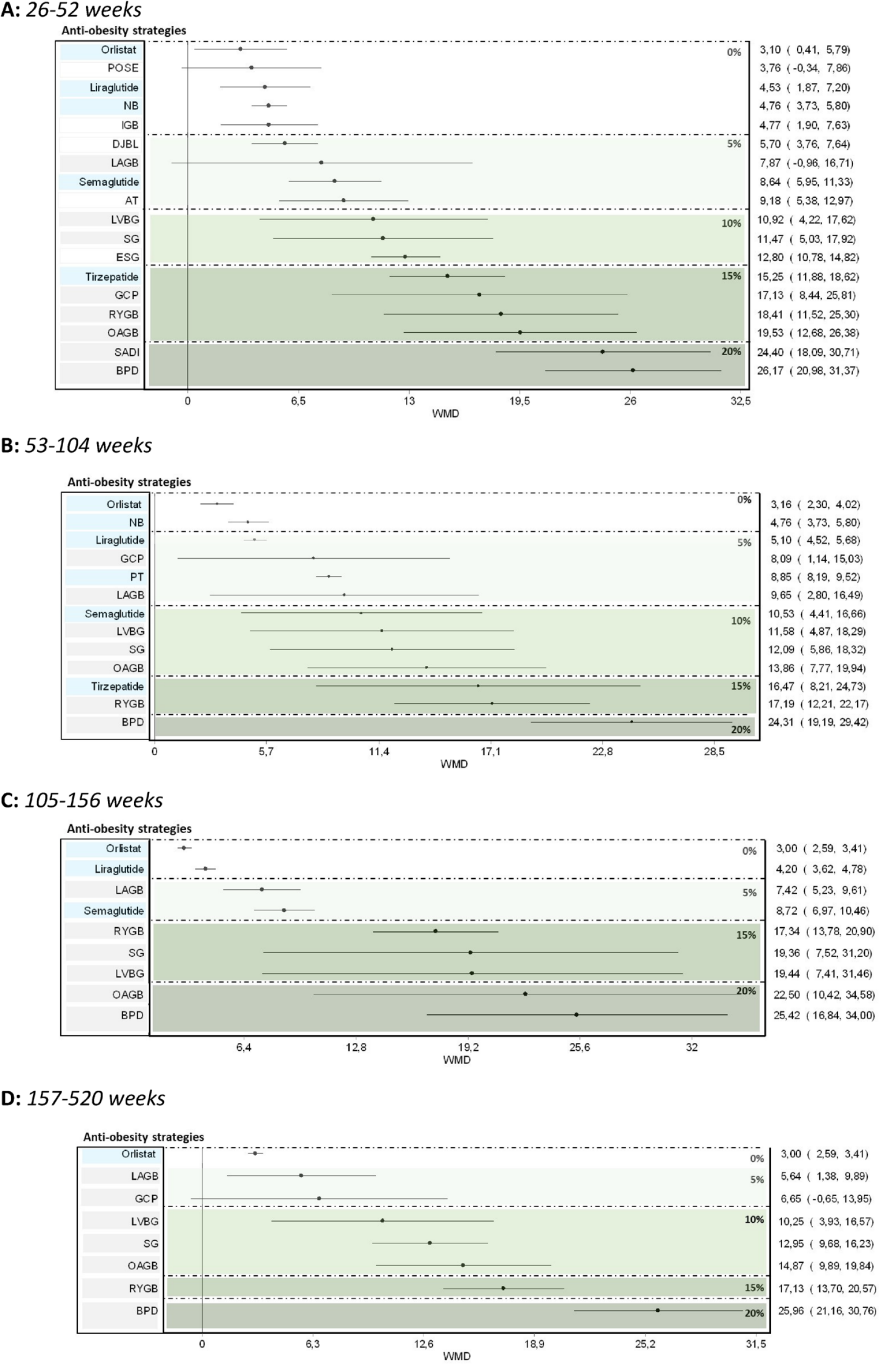
Forest plots for TBWL% at (A) 26–52 weeks, (B) 53–104 weeks, (C) 105–156 weeks, and (D) 156–520 weeks. AT, aspiration therapy; BPD, biliopancreatic diversion; ESG, endoscopic sleeve gastroplasty; GCP, greater curvature plication gastric; IGB, intragastric balloon; LAGB, laparoscopic adjustable gastric banding; LVGB, laparoscopic vertical banded gastroplasty; NB, naltrexone/bupropion; OAGB, one‐anastomosis gastric bypass; POSE, primary obesity surgery endoluminal; PT, phentermine/topiramate; RYGB, Roux‐en‐Y gastric bypass; SADI, single anastomosis duodenal switch; SG, sleeve gastrectomy; WMD, weighted mean difference. [Color figure can be viewed at wileyonlinelibrary.com]

Figure 2 illustrates the graphical representation of the geometry of all networks of interventions at each time point, while Figure 3 and Table 1 present the efficacy results from NMA on weight loss at various time points.

**TABLE 1:** Efficacy and safety of each strategy at different time points. [Color table can be viewed at wileyonlinelibrary.com]

Intervention	TBWL (%)				Any SAE (OR)			
	26–52	53–104	105–156	≥ 156	26–52	53–104	105–156	≥ 156
	***Obesity management medications***							
Orlistat	3.1	3.2	3.0	3.0	1.16	1.27	*	0.00
Liraglutide	4.5	5.1	4.2	—	1.01	1.29	*	*—*
Naltr./Bupr.	4.8	4.8	—	—	1.12	1.12	*—*	*—*
Phen./Topir.	*—*	8.8	*—*	*—*	*—*	1.28	*—*	*—*
Semaglutide	8.6	10.5	8.7	*—*	0.51	0.91	*	*—*
Tirzepatide	15.2	16.5	*—*	*—*	1.00	0.91	*—*	*—*
	***Endoscopic bariatric procedures***							
POSE	3.8	*—*	*—*	*—*	3.12	*—*	*—*	*—*
IGB	4.8	*—*	*—*	*—*	3.40	*—*	*—*	*—*
AT	9.2	*—*	*—*	*—*	2.52	*—*	*—*	*—*
ESG	12.8	*—*	*—*	*—*	4.42	*—*	*—*	*—*
	***Metabolic bariatric surgery***							
LAGB	7.9	9.6	7.4	5.6	*	1.84	*	8.06
GCP	17.1	8.1	*—*	6.6	1.68	*—*	*	10.75
LVBG	10.9	11.6	19.4	10.2	*	*—*	*	5.84
SG	11.5	12.1	19.4	13.0	1.68	*—*	*	1.87
OAGB	19.5	13.9	22.5	14.9	3.54	*—*	*	1.39
RYGB	18.4	17.2	17.3	17.1	2.24	5.0	*	5.84
SADI	24.4	*—*	*—*	*—*	*	*—*	*—*	*—*
BPD	26.2	24.3	25.4	26.0	*	*	*	22.78

*Note*: Dashes (—) indicate no data available for that time point. Asterisk (*) indicates that data were not derived from NMA (lack of comparisons between the intervention and the reference category or few trials retrieved for that time point). The safety evaluation for these interventions (color's intensity means a higher risk of serious adverse events [SAE]) has been performed based on a sensitivity analysis of the overall incidence of SAE at the endpoint (Table S10). TBWL (%): Dark green, TBWL < 15%; Light green, TBWL 10‐14.9%; Lighter green, TBWL 5‐9.9%; Neutral color, TBWL < 5%; Any SAE (OR): Green, no increased risk; Neutral color, no information; Red, significantly increased risk.

Abbreviations: AT, aspiration therapy; BPD, biliopancreatic diversion; ESG, endoscopic sleeve gastroplasty; GCP, greater curvature plication gastric; IGB, intragastric balloon; LAGB, laparoscopic adjustable gastric banding; LVGB, laparoscopic vertical banded gastroplasty; Naltr./Bupr., naltrexone/bupropion; OAGB, one‐anastomosis gastric bypass; Phen./Topir., phentermine/topiramate; POSE, primary obesity surgery endoluminal; RYGB, Roux‐en‐Y gastric bypass; SADI, single anastomosis duodenal switch; SG, sleeve gastrectomy; TBWL, total body weight loss.

#### 3.4.1 Primary Endpoint

##### 3.4.1.1 At Different Time Points

###### 3.4.1.1.1 26–52 Weeks

Table S6 shows the principal characteristics of NMA at 26–52 weeks, with information on the number of interventions, studies, patients included, pairwise comparisons with direct data, and multiarm studies.

At 26–52 weeks (*N* = 95 studies), all treatments showed a significant effect versus reference (LSI/none/placebo), except POSE surgery and LAGB. The estimated TBWL% was greater than 10% for most surgical procedures and tirzepatide. Funnel plot visual analysis did not suggest any relevant publication bias. Direct and indirect estimates are reported in Table S6. Figure 3, Figure S6, and Table S7 show no overall evidence of inconsistency (*H* = 1.07), with the notable exception of LAGB (Figure S6 and Table S7).

Since transitivity concerns in adopting this heterogeneous reference category (i.e., LSI/none/placebo) cannot be completely ruled out, we explored several possible modifiers across the three different types of control groups. The mean age, mean BMI, proportion of women, and trials enrolling participants with diabetes were 45 years, 36 kg/m^2^, 73%, and 57% for trials versus LSI, 44.9 years, 37.6 kg/m^2^, 70%, and 44% for trials versus no intervention, and 50 years, 36 kg/m^2^, 67%, and 41% for placebo‐controlled trials (all *p* > 0.20, with the exception of age: *p* = 0.004). Since it was not possible to assume the populations included in the three comparator groups (i.e., LSI, no intervention, and placebo) as homogeneous, we decided to perform a sensitivity post hoc NMA considering the placebo group as the reference category. When considering placebo as the reference category, non‐pharmacological (i.e., MBS and EBP) treatments showed, on average, a trend toward lower reduction of TBWL% in comparison with the principal analyses (i.e., LSI/placebo/none category) as reported in online Supporting Information (Figures S7–S10). In a further sensitivity post hoc analysis, when excluding high‐quality trials, non‐pharmacological (i.e., MBS and EBP) treatments showed, on average, a trend toward higher reduction of TBWL% in comparison with the principal analyses (i.e., LSI/placebo/none category; Figure S11).

###### 3.4.1.1.2 53–520 Weeks

Only a limited number of comparisons (Tables S8–S10) were available at 53–104 weeks (*N* = 47 studies; *H* = 1.05; Table S11), 105–156 weeks (*N* = 33 studies; *H* = 1.03; Table S12), and 157–520 weeks (*N* = 28 studies; *H* = 1.04; Table 13). Funnel plot visual analysis did not suggest any relevant publication bias. No relevant inconsistency was observed for any of the considered time points (Tables S11–S13 and Figures S12–S14).

No long‐term data were available for EBP and for most OMMs. For most of the assessed treatments, the estimated efficacy was similar to that at 26–52 weeks, except for LAGB and GCP, the effects of which seem to reduce after 104 weeks (Figure 3).

The limited number of retrieved studies and possible comparisons between different antiobesity strategies prevented reliable subgroup analyses for all these time points.

##### 3.4.1.2 For Classes of BMI

Although some trials (particularly for OMMs) allowed the enrollment of patients with BMI between 27 and 30 kg/m^2^, the mean BMI of patients enrolled for MBS was above 30 kg/m^2^ (Table S5), with the only exception of one trial on RYGB versus LSI, showing a significant weight loss in an Asian population [22]. Only one trial (orlistat vs. placebo) reported a subgroup analysis for patients with BMI < 30 kg/m^2^, with a weight loss of 2.2% at 1 year [23]. A larger amount of data was available for BMI 30–34.9 and 35–39.9 kg/m^2^, allowing for NMA at 26–52 and 53–104 weeks. Data on patients with BMI ≥ 40 kg/m^2^ were available for most surgical procedures, even for long‐term follow‐up, and for semaglutide up to 2 years. The results of NMA at different time points for each class of BMI are reported in Figures S15–S23 and summarized in Table 2. MBS appeared to be more effective in patients with a higher baseline BMI. BPD and SADI, extensively tested in patients with BMI ≥ 40 kg/m^2^, did not appear to produce a more significant weight loss than RYGB or OAGB in the BMI ≥ 40 kg/m^2^ class.

**TABLE 2:** Total body weight loss (TBWL%) for each obesity management strategy for different baseline BMI subgroups. [Color table can be viewed at wileyonlinelibrary.com]

BMI (kg/m^2^)	26–52 weeks				53–104 weeks				105–156 weeks				≥ 156 weeks			
	< 30	30–34.9	35–39.9	40+	< 30	30–34.9	35–39.9	40+	< 30	30–34.9	35–39.9	40+	< 30	30–34.9	35–39.9	40+
	***Obesity management medications***															
Orlistat	2.2^a^	2.4	3.2	—	—	—	3.2	—	—	—	—	—	—	—	—	—
Liraglutide	—	5.7	4.3	—	—	—	5.1	—	—	—	—	—	—	—	—	—
Semaglutide	—	9.4	10.8	10.4	—	10.7	11.7	10.5	—	—	—	—	—	—	—	—
Naltr/Bupr.	—	—	—	—	—	—	4.8	—	—	—	—	—	—	—	—	—
Phen/Topir.	—	—	—	9.3	—	—	8.6	—	—	—	—	—	—	—	—	—
Tirzepatide	—	19.5	14.7	—	—	—	14.4	—	—	—	—	—	—	—	—	—
	***Endoscopic bariatric procedures***															
IGB	—	6.4	4.3	—	—	—	—	—	—	—	—	—	—	—	—	—
POSE	—	—	3.8	—	—	—	—	—	—	—	—	—	—	—	—	—
AT	—	—	—	9.2	—	—	—	—	—	—	—	—	—	—	—	—
ESG	—	—	12.8	—	—	—	—	—	—	—	—	—	—	—	—	—
	***Metabolic bariatric surgery***															
SG	—	14.4	13.0	19.5	—	—	11.9	18.4	—	—	—	18.1	—	—	—	20.3
LVBG	—	—	—	19.0	—	—	—	17.9	—	—	—	18.2	—	—	—	19.0
LAGB	—	—	7.8	20.8	—	—	8.9	21.4	—	—	—	12.7	—	—	—	12.6
GCP	—	—	—	22.7	—	—	—	14.4	—	—	—	—	—	—	—	—
SADI	—	—	—	24.4	—	—	—	—	—	—	—	—	—	—	—	—
OAGB	—	17.1	23.4	25.1	—	—	—	20.2	—	—	—	21.0	—	—	—	22.4
BPD	—	—	—	27.0	—	—	—	25.0	—	—	—	26.4	—	—	—	28.2
RYGB	15.5^a^, ^b^	18.3	18.7	28.8	12.5	11.3	18.6	24.6	—	—	—	20.4	—	—	—	22.3

*Note*: TBWL (%): Dark green, TBWL < 15%; Light green, TBWL 10‐14.9%; Lighter green, TBWL 5‐9.9%; Neutral color, TBWL < 5%; Any SAE (OR): Green, no increased risk; Neutral color, no information; Red, significantly increased risk.

Abbreviations: AT, aspiration therapy; BPD, biliopancreatic diversion; ESG, endoscopic sleeve gastroplasty; GCP, greater curvature plication gastric; IGB, intragastric balloon; LAGB, laparoscopic adjustable gastric banding; LVGB, laparoscopic vertical banded gastroplasty; Naltr./Bupr., naltrexone/bupropion; OAGB, one‐anastomosis gastric bypass; Phen./Topir., phentermine/topiramate; POSE, primary obesity surgery endoluminal; RYGB, Roux‐en‐Y gastric bypass; SADI, single anastomosis duodenal switch; SG, sleeve gastrectomy.

^a^ Data derived from traditional meta‐analysis versus placebo.

^b^ Asian populations.

##### 3.4.1.3 For Diabetes Status

Some trials enrolled only patients with or without diabetes or provided subgroup analyses for individuals with or without diabetes. When data on people with diabetes were analyzed separately (Table S14 and Figures S24–S31), semaglutide and tirzepatide appeared to produce a lesser weight loss in those without diabetes. A similar phenomenon was observed for MBS, at least at 105–156 weeks.

##### 3.4.1.4 Risk of Bias (RoB) NMA Assessment

Figure S32A–D shows the RoB for each comparison (vs. LSI/placebo/none) at 26–52, 53–104, 105–156, and 157–520 weeks, respectively. Both panels reported, on average, lower risks for pharmacological comparisons versus surgical and endoscopic procedures. The overall NMA RoB was reported in Figure S33A–D, showing a confidence rating ranging from “moderate” to “low” and “low” to “very low” for pharmacological and non‐pharmacological treatments at each time point, respectively.

#### 3.4.2 Secondary Endpoints

##### 3.4.2.1 Serious Adverse Events (SAE)

Data on SAE (see definition in the Methods section) are reported in Figures S34–S39 and summarized in Table 1. Endoscopic and surgical procedures were generally associated with a greater risk of SAE than pharmacological treatments. In the long term, BPD appeared to produce a greater incidence of SAE than other types of MBS. Some treatments could not be assessed for this variable at some time points (i.e., 105–156 weeks) because of insufficient data for NMA (*n* = 9 RCTs). A sensitivity analysis was performed, estimating the proportion of patients with reported SAE in all available arms for each treatment. This analysis confirmed a higher risk of SAE with BPD, SADI, and LVBG and a lower risk with OMMs (Table S15).

##### 3.4.2.2 Mortality

The paucity of events for all‐cause mortality did not allow a formal NMA. Figures S40 and S41 report meta‐analysis results comparing interventions with placebo or active comparators. The number of recorded events for each comparison was smaller than 50, with the only exception of semaglutide, which was associated with a significant reduction of all‐cause mortality when compared to placebo (MH‐OR 0.81 [0.70–0.92]; *I* ^2^ 0%, *p* = 0.002; Figures S28 and S29). No other significant result was observed for any available comparisons between active comparators.

##### 3.4.2.3 Quality of Life (QoL)

Only a few trials reported data on QoL, using different scales (10, 10, 10, 5, and 5 trials with IWQOL‐Lite [17, 28, 31, 32, 33, 34, 35, 36, 37, 38], SF‐36 General Health [20, 21, 38, 39, 40, 41, 42, 43, 44, 45], SF‐36 Physical Role Functioning [31, 32, 35, 37, 41, 42, 43, 45, 46, 47], SF‐36 Physical and Mental Components [39, 40, 43, 48, 49], respectively). The paucity of data did not allow for NMA, only traditional meta‐analysis (Figure S42). NB, semaglutide, IGB, and POSE, but not liraglutide and tirzepatide, were associated with an improvement of IWQOL versus placebo (Figure S42A). Only one active‐controlled trial (i.e., liraglutide vs. orlistat) reported data on IWQOL, showing better scores for liraglutide [17]. RYGB and BPD reported better endpoint scores for SF‐36 General Health compared to LSI/no therapy, whereas no between‐group differences were observed for IGB, orlistat, and liraglutide. Some trials compared RYGB with BPD (one trial) and RYGB with SG (three trials), with no significant between‐group differences (Figure S42B). When analyzing the subscale “Physical role functioning,” RYGB and semaglutide were associated with significantly improving reported QoL versus placebo/LSI/no therapy. IGB, liraglutide, orlistat, and tirzepatide did not significantly change QoL scores at the endpoint (Figure S42C). Only five trials reported information on SF‐36 Physical and Mental Components subscales, showing no significant differences versus placebo/LSI/no therapy for semaglutide, IGB, and LAGB (one trial for each intervention). Two trials comparing RYGB versus SG did not report significant between‐group differences (Figure S42D).

## 4 Discussion

The current belief of many experts relies on the assumption that surgical treatment is more effective and associated with a higher risk of adverse events compared to OMMs. The comprehensive assessment of all available evidence coming from RCTs does not entirely support this assumption. New definitions of obesity, beyond BMI‐based measures, challenge professionals involved in obesity management, aiding clinical decision‐making and prioritizing the appropriateness of public health strategies [50]. Both OMMs and surgical procedures are widely heterogeneous in their efficacy and safety, with newer agents (i.e., semaglutide and tirzepatide) and some types of MBS (i.e., gastric bypass, SG, SADI, and BPD) being more effective than older OMMs and lower‐efficacy procedures, such as GCP and LAGB. This paper aims to provide health care professionals and policy makers with adequate information about the available evidence‐based treatment options for obesity, especially in light of the newly published definition and diagnostic criteria of clinical obesity. As stated by the Lancet Diabetes and Endocrinology Commission, clinical obesity is a condition of illness directly resulting from excess adiposity, which can widely affect any organ or tissue function. Nevertheless, the diagnosis of clinical obesity requires one or both of the specific criteria (i.e., evidence of impaired organ or tissue function related to obesity and *adiposopathy*, signs and symptoms or diagnostic tools showing obesity‐related organ dysfunction with limitation of daily activities).

Older agents (i.e., orlistat, NB, and liraglutide) produce a relatively small weight loss (less than 5% of initial body weight) among OMMs. The combination of phentermine and topiramate (PT) produces a weight loss of between 5% and 10% in the short term, with no available data in the longer term. This could be explained by the various pharmacological interactions, contraindications in patients affected by psychosocial disorders, and lack of relevant nutrient‐sensitive hormone pathways. Newer agents (semaglutide and tirzepatide) seem more effective in weight reduction. Unfortunately, there is only one direct comparison with a duration of at least 52 weeks comparing two different OMMs. Relative estimates of efficacy rely only on indirect comparisons calculated with NMA. The effect on body weight is maintained for at least 3 years, but longer‐term trials are available only for orlistat. The safety of OMMs is acceptable, with an odds ratio of experiencing SAE below 1.30 for all agents.

RCTs in metabolic surgery are not sufficiently powered to show mortality reduction, but reassuring evidence exists from large epidemiological studies. Specifically designed cardiovascular outcome trials are available only for semaglutide [51] and NB [52], while a trial on tirzepatide is currently ongoing [53]. Semaglutide, unlike NB, was reported to be associated with a significant reduction in the incidence of major cardiovascular events in people with obesity with previous cardiovascular events [51]. These findings align with those obtained by a higher number of cardiovascular outcome trials performed in patients with T2DM, suggesting that some classes of drugs (i.e., GLP‐1RA) could have beneficial cardiovascular effects even in patients affected by obesity [54]. However, these putative beneficial effects should be proven through specifically designed trials, and different OMMs could likely have different impacts on cardiovascular outcomes. OMMs approved from 1999 to 2014 (i.e., sibutramine) were shown to increase, rather than reduce, cardiovascular risk and related mortality [55]. However, the available cardiovascular trials on NB and sibutramine are heterogeneous in sample size, case mix, and treatment duration, preventing any definitive conclusion. Unfortunately, no specific cardiovascular outcome data are available for other OMMs, some of which (e.g., PT) could theoretically affect the cardiovascular system [56]. Moreover, phentermine is generally not prescribed to people taking selective serotonin reuptake inhibitors due to a theoretical risk of cardiovascular disease and heart failure [57]. In fact, despite the relevant burden of cardiovascular disease associated with obesity, regulatory authorities do not routinely require cardiovascular outcome data for new obesity drugs, as in other adjacent therapeutic areas [58].

Evidence on endoscopic bariatric procedures introduced more recently than most surgical treatments and most drugs is still limited, particularly in the longer term. The category of EBP is very heterogeneous, including both temporary and permanent interventions: IGB and AT are typically applied for a limited amount of time to induce relevant weight loss for specific aims (e.g., to reduce the risk for surgical intervention). Conversely, POSE and ESG are designed for longer‐term use. The amount of weight loss determined by ESG is similar to that of the most effective OMMs and some surgical procedures. In addition, ESG appears to be more effective than POSE. The number of reported adverse events is higher than that of OMMs and not very dissimilar from surgery.

The number of available trials for MBS is remarkable, although sample sizes are usually small. Unlike OMMs, for MBS, there is also a substantial body of evidence derived from direct head‐to‐head studies, which both allow traditional meta‐analyses for comparisons and strengthen the reliability of NMA. Notably, the results from traditional meta‐analyses on MBS differ from those obtained with NMA. The assumption that different surgical strategies have a more significant impact on weight loss is already well acknowledged [6, 12, 13, 59, 60]. As previously reported, in the present analysis, BPD and SADI produce a more significant weight loss than RYGB and OAGB, which, in turn, seem more effective than SG, LVBG, GCP, and LAGB. The therapeutic effects are substantially maintained in the long term, except for LAGB and GCP. Despite the rate of weight reduction during any obesity treatment tending to decline over time, reaching a weight plateau, this phenomenon seems to be less pronounced with MBS. This could be explained by the specific anatomical configuration and hypoabsorptive components of the procedures related to reduced gastrointestinal transit time and limited contact with brush border enzymes. On the contrary, little is known about the durability of OMMs and EBP. Long‐term data are available only for orlistat and, very recently, for tirzepatide (i.e., 3‐year follow‐up of SURMOUNT‐1 trial), preventing any reliable conclusions [61]. On the other hand, SAE are more frequent with those approaches that are associated with more significant weight loss, BPD and SADI. Due to the small size of the enrolled samples, mortality data are insufficient to draw any reliable conclusion.

BPD and SADI seem to have greater effects on body weight loss. However, it should be considered that the few RCTs on these interventions are all performed in patients with higher baseline BMI (i.e., BMI ≥ 40 kg/m^2^), who are more likely to benefit from any type of antiobesity strategy. Interestingly, when analyzing data based on BMI at entry, RYGB is not inferior to BPD and SADI. Moreover, these latter surgical interventions are at higher risk for SAE than other types of MBS. On the other hand, OMMs seem to perform with similar efficacy across different BMI classes. This should be taken into account when prescribing OMMs to patients affected by overweight or lower degrees of obesity. In addition, putative beneficial effects of treating patients at lower risk for incident obesity‐associated comorbid conditions are far from being proven, as shown by the present systematic review with very few data on overweight patients (one trial on lorcaserin reporting a modest, albeit significant, effect on TBWL% in comparison with placebo [23] and one trial on RYGB performed on an Asian sample [22]). Quite interestingly, the placebo‐controlled cardiovascular outcome trial on semaglutide [51] performed in patients affected by overweight/obesity and previous cardiovascular disease reported subgroup analyses for different classes of BMI, showing a significantly lower incidence of major cardiovascular events in favor of the treatment, even in patients with BMI ranging from 27 to 30 kg/m^2^.

Another interesting preplanned subgroup analysis of the present meta‐analysis is that performed for diabetes status. RCTs excluding patients with T2DM were more likely to achieve a greater efficacy in body weight loss than RCTs performed on patients with T2DM. This is not surprising and is expected mainly due to body weight recovery after amelioration of glucose control (i.e., fluid retention, increase of fat‐free mass, etc.).

Some limitations of the present NMA should be considered when interpreting the results. The quality of trials is not homogeneous, possibly introducing some biases. The open‐label design, which is inevitable in the case of comparisons between surgical and non‐surgical treatments, could produce a bias because of a possible placebo effect of surgery. Moreover, to compare different surgical and non‐surgical strategies for treating obesity, we were forced to choose a heterogeneous reference category (i.e., placebo, LSI, and no therapy). Most RCTs performed on OMMs are usually placebo‐controlled, whereas EBP and MBS are often compared to LSI or no therapy (i.e., uncontrolled studies). This decision could be a source of heterogeneity, and it could have introduced a bias against OMMs, underestimating the actual effects on the primary endpoint. Therefore, we performed several sensitivity analyses (i.e., analyzing TBWL% at different time points, for different baseline BMI categories, and diabetes status), which showed no relevant differences in results across follow‐up durations, baseline BMI, and diabetes status for different treatment strategies. In addition, to try to overcome the heterogeneity derived from the choice of a combined reference category, further post hoc sensitivity NMA has been performed considering placebo as the reference category. By comparing the results of this latter NMA at 26–52 weeks with those of the principal NMA (i.e., reference category: LSI/no therapy/placebo), no relevant differences were observed, except for a slightly lower efficacy of surgical procedures. Unfortunately, these sensitivity analyses could not be performed for other time points due to the relatively small number of longer‐term studies. Another possible source of heterogeneity when interpreting the results of this paper can be represented by the choice to consider together surgical and non‐surgical procedures, particularly for the safety profile. In fact, adverse events for OMMs are considerably different from those reported during and after a surgical intervention, making this comparison particularly problematic.

Nevertheless, other limitations of the present study should be highlighted. Firstly, data on long‐term adherence to treatments are lacking, which might be explained by the recent market placement of newer OMMs. Moreover, the study does not address adherence rates for different interventions, particularly for OMMs and LSIs; this is of paramount importance since many patients struggle daily with long‐term adherence to pharmacological treatments due to side effects, cost, or behavioral factors. On the other hand, surgical and endoscopic options may also have adherence‐related challenges, such as dietary restrictions or low follow‐up compliance. For these reasons, further studies should assess adherence rates and determine their impact on long‐term weight loss and health care outcomes. Moreover, the follow‐up and treatment duration significantly differ from RCTs performed on MBS and those performed on OMMs and EBP. This latter point led to several concerns in comparing different therapeutic strategies, which have been addressed by performing additional preplanned sensitivity analyses divided by different time points. In addition, although the EMA and FDA have approved several OMMs for long‐term use, their efficacy (durability), safety, and cost‐effectiveness are far from being fully explored in the long term. On the other hand, there are several RCTs on MBS reporting their effectiveness even after 10 years, as shown in the present NMA. Long‐term high‐quality RCTs on OMMs are therefore urgently required to perform more reliable comparisons across different antiobesity strategies.

Secondly, this NMA is based only on RCTs, which often recruit specific patient populations and might not entirely reflect real‐world effectiveness, where factors like advanced age, insurance coverage, ethnicity, burden of obesity related‐medical conditions (i.e., heart failure, respiratory disturbances including sleep apnea or reduced chest wall lung compliance, reproductive dysfunctions), socioeconomic status, and patient motivation play a role. Future analyses should include real‐world evidence from cohort studies, national registries, and electronic health records to improve generalizability and applicability. Moreover, several NMA studies showed wide ranges of CIs for some antiobesity strategies (particularly MBS). This is due to the relatively small sample sizes for some of the included outcomes, thus limiting the results' reliability. Nevertheless, the effects of any antiobesity strategy rely on aspects other than the impact on weight loss, such as controlling obesity‐associated medical conditions. Our meta‐analysis did not report data on other outcomes due to the high heterogeneity across different strategies and studies in defining obesity‐associated medical conditions.

Another relevant aspect should be underlined, concerning the lack of standardized definitions for weight loss success, since different studies may adopt variable thresholds for defining “clinically meaningful weight loss” (i.e., 5% vs. 10% TBWL). This inconsistency might impact the comparability of results across different interventions. Future research should be aware of this issue, adopt a standardized weight loss threshold (e.g., ≥ 10% TBWL), and consistently define metabolic outcomes.

Since transitivity concerns in adopting a heterogeneous reference category (i.e., LSI/none/placebo) could not be completely ruled out, we explored several possible modifiers across the three different types of control groups, finding a significantly higher mean age for pharmacological RCTs. This means that it was not possible to assume the populations in the three comparator groups (i.e., LSI, no intervention, and placebo) as homogeneous. To assess this possible bias, a sensitivity post hoc NMA was conducted, considering the placebo group as the reference category, which showed, on average, a lower efficacy of MBS and EBP compared to the results obtained using LSI/placebo/none as the reference category.

Due to considerably heterogeneous effectiveness across individual surgical (and pharmacological) interventions, no meta‐analysis could have been performed using categorical groups (i.e., comparisons between MBS, OMMs, and EBP).

Finally, a cost‐effectiveness analysis is not provided, thus hindering a proper assessment of the economic burden and cost‐effectiveness of different obesity‐management options. This represents a relevant factor for clinicians, policy makers, and insurance companies for an appropriate and precise definition of indication and coverage of various treatments. Not to mention, on one hand, newer obesity medications (i.e., semaglutide, tirzepatide) are expensive and accessibility may be limited; on the other hand, surgical and endoscopic procedures have high upfront costs but may lead to long‐term savings by reducing the economic and social burden related to organ dysfunction and individuals' ability to conduct average daily activities related to clinical obesity. A cost‐effectiveness analysis comparing various interventions should be included in future NMA studies to provide valuable insights for health care decision‐making and policy‐making.

## 5 Conclusion

The results obtained are of interest to clinicians involved in obesity management. For the first time, evaluating only RCTs with GRADE methodology, different approaches were analyzed in different categories of patients, giving a clearer overall picture of their effectiveness. MBS was associated with greater weight loss, which was superior to other treatments, especially in the long term. EBP, except for ESG, showed a lower efficacy in weight loss compared to newer OMMs (i.e., semaglutide and tirzepatide), which were competitive in the short term with some MBS procedures.

Some surgical interventions were less effective long term (LAGB and GCP) than others. BPD should be used in selected patients because it was only slightly superior to RYGB and OAGB, and it was associated with a higher risk of SAE. Nevertheless, the benefits of obesity management strategies go beyond BMI reduction, and a better weight loss outcome does not necessarily represent superiority of one treatment over another, without a thorough assessment of comorbidities and QoL. However, the lack of efficacy and safety in the longer term and concerns about OMMs' affordability should be carefully considered when making attempts to compare surgical and non‐surgical antiobesity strategies, which deserve further investigation.

## Author Contributions

M.D.L., R.V.C., A.B., and M.M. were involved in the study design, interpretation, writing the manuscript, and revising the manuscript. M.M., E.M., and B.R. were involved in the data collection and statistical analyses. N.D.L., G.N., T.B.Z.P., R.B., S.B., L.B., and P.S. were involved in writing and revising the manuscript.

## Conflicts of Interest

The authors declare no conflicts of interest.

## Supporting information


**Data S1:** oby70083‐sup‐0001‐Supinfo.pdf.

## Data Availability

The data that support the findings of this study are available from the corresponding author upon reasonable request. All references are linked to the dataset in online Supporting Information.
