# Supplementary material for: Efficacy and Safety of Pharmacological, Endoscopic, and Surgical Treatments for Obesity: A GRADE‐Based Network Meta‐Analysis
Source: Obesity (Silver Spring). 2026 Jan 15;34(2):279–93. doi: 10.1002/oby.70083 (PMC12850565; doi:10.1002/oby.70083)
Supplement: Supplementary file 1 — Data S1: oby70083‐sup‐0001‐Supinfo.pdf. [file OBY-34-279-s001.pdf]

Supplementary appendix

Supplement to:

Efficacy and Safety of Pharmacological, Endoscopic, and Surgical Treatments for Obesity. A Network Meta-Analysis of Randomized Controlled Trials.

Maurizio De Luca[1] MD, Ricardo V Cohen[2] MD, Amanda Belluzzi[1] MD, Luigi Angrisani[3]MD, Giuseppe Navarra[4] MD, Nicola Di Lorenzo[5] MD, Tarissa BZ Petry[2] MD, Paolo Sbraccia[6] MD, Luca Busetto[7] MD, Silvio Buscemi[8] MD, Rocco Barazzoni[9] MD, Benedetta Ragghianti[10] MD, Edoardo Mannucci[10] MD, Matteo Monami[10] MD.

1- Department of General Surgery, Rovigo Hospital, Italy
2- Center for Obesity and Diabetes, Oswaldo Cruz German Hospital, São Paulo, Brazil
3- University Federico II of Naples, Italy
4- University of Messina, Italy
5- University La Sapienza, Rome, Italy
6- University Tor Vergata, Rome, Italy
7- University of Padua, Padua, Italy
8- University Hospital Policlinico "P. Giaccone", Palermo, Italy
9- University Hospital, Trieste, Italy
10- Department of Biomedical, Experimental and Clinical Sciences, University of Florence

Corresponding author:
Edoardo Mannucci
Department of Biomedical, Experimental and Clinical Sciences, University of Florence, Florence, Italy.
edoardo.mannucci@unifi.it.

Table of contents

Figure 1S – Trial flow summary 4

Figure 2S – Risk of bias graph: review authors' judgements about each risk of bias item presented as percentages across all included studies. 5

Figure 3S – Risk of bias summary: review authors' judgements about each risk of bias item for each included study........ 6

Figure 4S – Effects of individual anti-obesity strategy on TBWL% at endpoint in placebo- (Panel A) and active- (Panel B) controlled trials. 7

Figure 5S – Network meta-analysis for different anti-obesity strategies. Network plot for TBWL% at 26-52 weeks (RCTs with a mean BMI at entry: Panel A: 30-34.9 kg/m2; B 35-39.9 kg/m2; C: > 39.9 kg/m2). 10

Figure 6S – Network meta-analysis for different anti-obesity strategies. Forest plot for TBWL% at 26-52 weeks (RCTs with a mean BMI at entry: Panel A: 30-34.9 kg/m2; B 35-39.9 kg/m2; C: > 39.9 kg/m2). 12

Figure 7S – Effects of different anti-obesity strategies on TBWL% at 53-104 weeks in RCTs with a mean BMI at entry 30-34.9 kg/m2. Panel A: Interventions versus Placebo/LSI; Panel B: Active comparisons). SG: Sleeve Gastrectomy; OAGB: One-anastomosis gastric bypass; RYGB: Roux-en-Y Gastric By-Pass........ 14

Figure 8S – Network meta-analysis for different anti-obesity strategies. Network plot for TBWL% at 53-104 weeks (RCTs with a mean BMI at entry: Panel A: 35-39.9 kg/m2; B: > 39.9 kg/m2). 15

Figure 9S – Network meta-analysis for different anti-obesity strategies. Forest plot for TBWL% at 53-104 weeks (RCTs with a mean BMI at entry: Panel A: 35-39.9 kg/m2; B: > 39.9 kg/m2). 16

Figure 10S – Network meta-analysis for different anti-obesity strategies. Network plot for TBWL% at 105-156 weeks (RCTs with a mean BMI at entry > 39.9 kg/m2)........ 17

Figure 11S – Network meta-analysis for different anti-obesity strategies. Forest plot for TBWL% at 105-156 weeks (RCTs with a mean BMI at entry > 39.9 kg/m2)........ 18

Figure 21S – Network meta-analysis for different anti-obesity strategies. Forest plot for TBWL% at 157-520 weeks. RCTs enrolling (panel A) and non-enrolling (Panel B) subjects with diabetes mellitus. 28

Secondary endpoints........ 29

Figure 22S – Network meta-analysis for different anti-obesity strategies. Network plot for risk of SAE at 26-52 weeks. 29

Figure 23S – Network meta-analysis for different anti-obesity strategies. Forest plot for risk of SAE at 26-52 weeks. 30

Figure 24S – Network meta-analysis for different anti-obesity strategies. Network plot for risk of SAE at 53-104 weeks. 31

Figure 25S – Network meta-analysis for different anti-obesity strategies. Forest plot for risk of SAE at 53-104 weeks. 32

Figure 26S – Network meta-analysis for different anti-obesity strategies. Network plot for risk of SAE at 157-520 weeks. 33

Figure 27S – Network meta-analysis for different anti-obesity strategies. Forest plot for risk of SAE at 157-520 weeks. 34

Figure 28S – Effects of different anti-obesity strategies on all-cause mortality at the endpoint. Panel A: Interventions versus Placebo/LSI/no therapy; Panel B: Active comparisons). OAGB: One-anastomosis gastric bypass; RYGB: Roux-en-Y Gastric By-Pass. 35

Figure 29 – Effects of each anti-obesity strategy on all-cause mortality at endpoint in placebo- and active-controlled trials. 39

Figure 29 – Effects of each anti-obesity strategy on quality of life at endpoint in placebo/LSI/no therapy- and active-controlled trials. 40
Tables .... **Error! Bookmark not defined.**
Table 1S – PRISMA checklist. 45
Table 2S – Detailed information on search strategy 49
Table 3S: Information collected for each trial 52
Table 4S. Excluded trials and reasons for the exclusion. 53
Table 5S. Principal baseline characteristics of the included studies 54
Table 6S – Direct and estimates of effects of different anti-obesity strategies on 26-52 weeks TBWL%. 60
Table 7S – Direct and estimates of effects of different anti-obesity strategies on 53-104 weeks TBWL%. 62
Table 8S – Direct and estimates of effects of different anti-obesity strategies on 105-156 weeks TBWL% 64
Table 9S – Direct and estimates of effects of different anti-obesity strategies on 105-156 weeks TBWL% 65
Table 9S – TBWL% of each anti-obesity strategy for diabetes status. 67
Table 10S – Sensitivity analysis assessing the overall incidence of SAE at endpoint for each intervention. Point estimates express the proportion of patients experiencing at least one SAE during each type of treatment. 68
References 69

Figure 1S – Trial flow summary

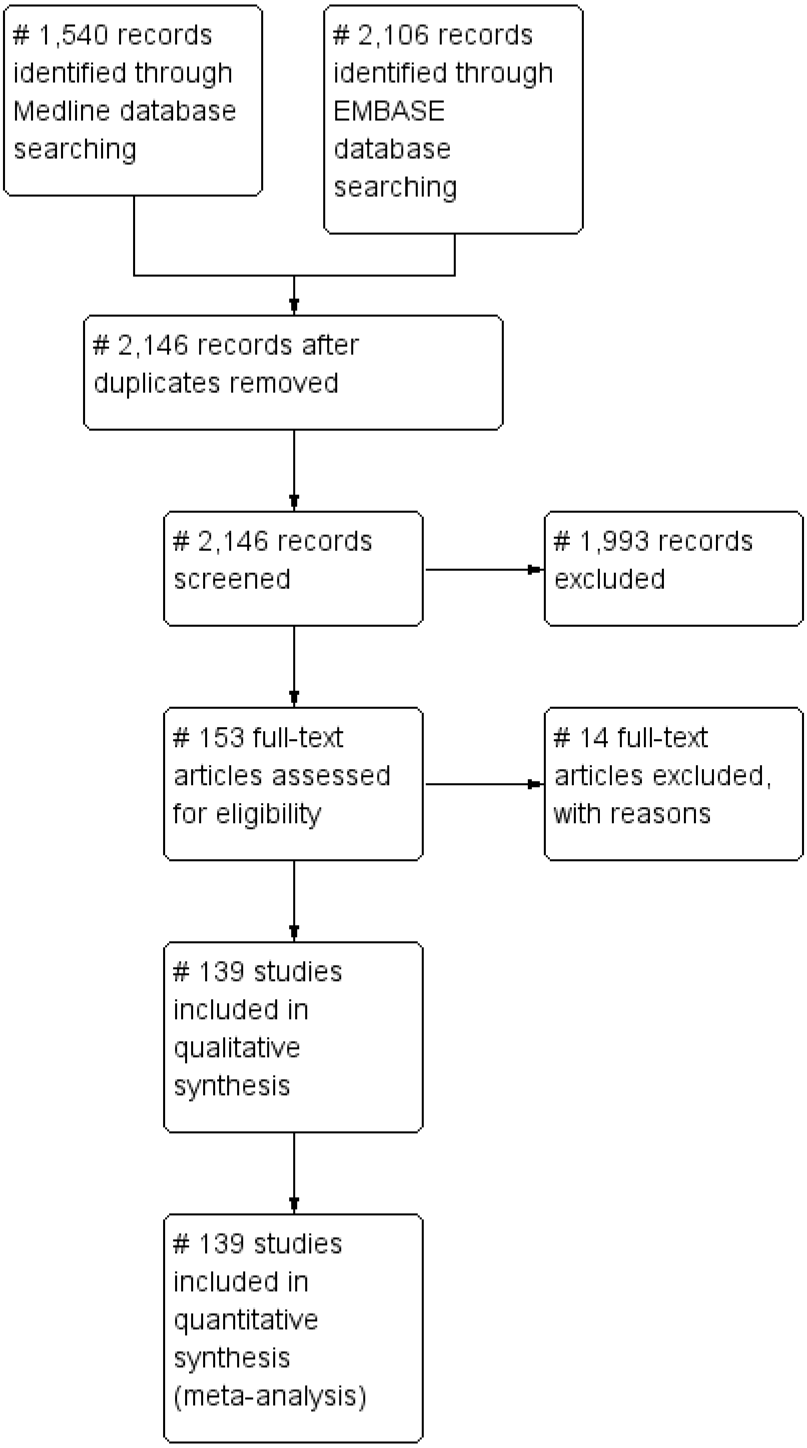

Figure 2S – Risk of bias graph: review authors' judgements about each risk of bias item presented as percentages across all included studies.

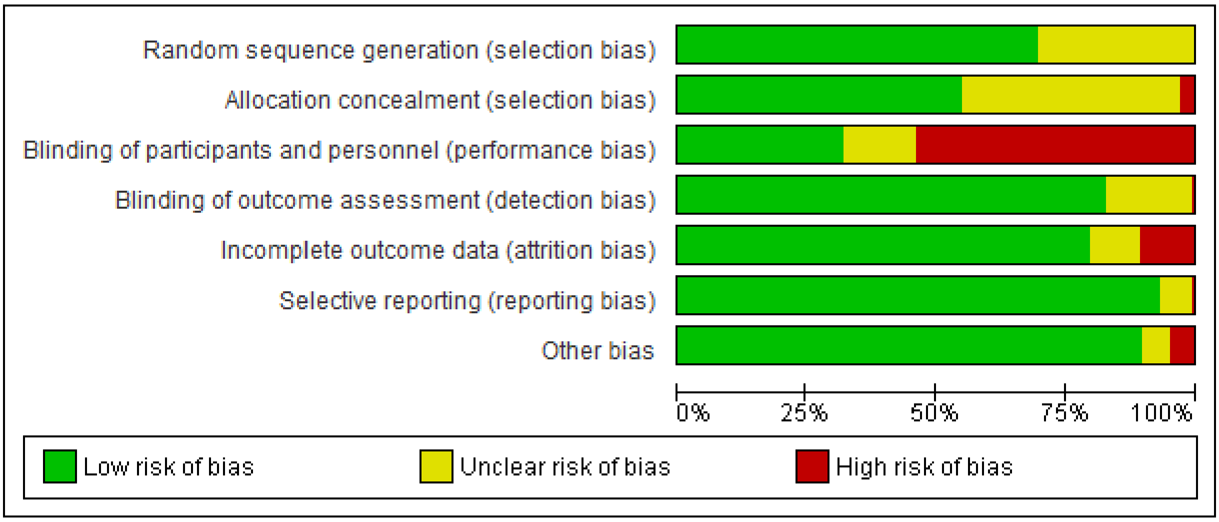

Figure 3S – Risk of bias summary: review authors' judgements about each risk of bias item for each included study.

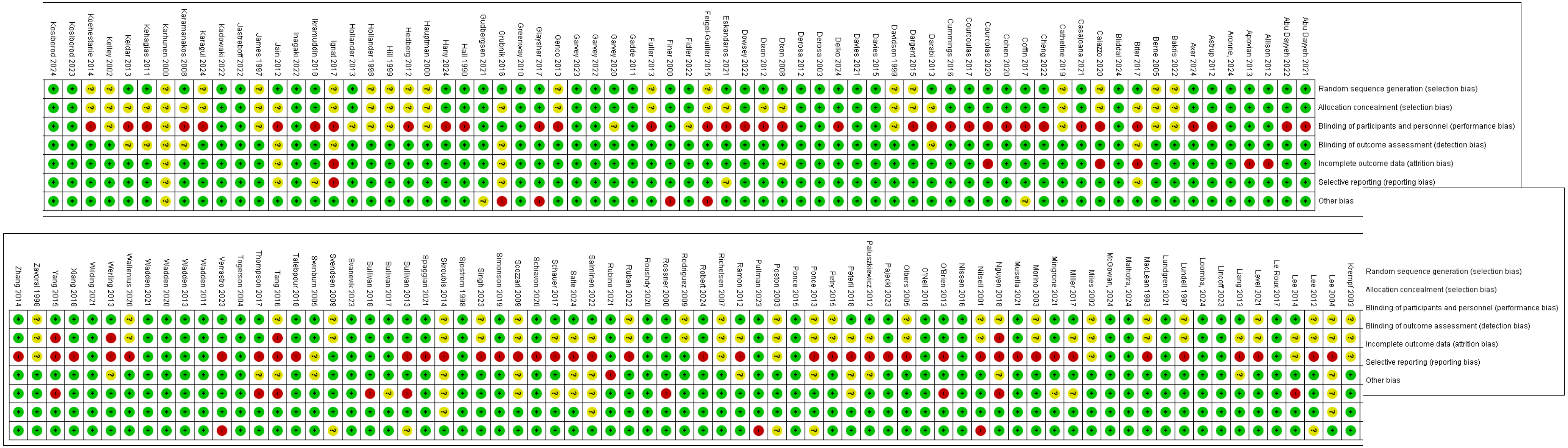

Figure 4S – Effects of individual anti-obesity strategy on TBWL% at endpoint in placebo- (Panel A) and active- (Panel B) controlled trials. Heterogeneity was assessed using I2 statistics and a value≥50% means a high grade of heterogeneity.

A

Study or Subgroup	Experimental Mean	Experimental SD	Experimental Total	Control Mean	Control SD	Control Total	Weight	Mean Difference IV, Random, 95% CI
**5.1.1 IGB**								
Ponce 2015	7.6	5.5	187	3.6	6.3	139	2.0%	4.00 [2.69, 5.31]
Sullivan 2018	6.6	5.1	198	3.4	5	189	2.0%	3.20 [2.19, 4.21]
**Subtotal (95% CI)**			**385**			**328**	**3.9%**	**3.50 [2.70, 4.29]**
Heterogeneity: Tau² = 0.00; Chi² = 0.90, df = 1 (P = 0.34); I² = 0%								
Test for overall effect: Z = 8.59 (P < 0.00001)								
**5.1.2 Liraglutide**								
Astrup 2012	7.5	10.6	93	1.7	10.9	98	1.8%	5.80 [2.75, 8.85]
Davies 2015	6	4	423	2	4	212	2.0%	4.00 [3.34, 4.66]
Garvey 2020	5.8	12.6	195	1.5	12.6	197	1.9%	4.30 [1.81, 6.79]
Le Roux 2017	6.1	7.3	1505	1.9	6.3	749	2.0%	4.20 [3.62, 4.78]
Lundgren 2021	14.5	7.1	98	8.8	7.1	97	1.9%	5.70 [3.71, 7.69]
O'Neil 2018	8.3	10.1	103	2.3	11.7	136	1.8%	6.00 [3.23, 8.77]
Wadden 2013	6.2	7.3	212	0.2	7	210	2.0%	6.00 [4.64, 7.36]
Wadden 2020	7.4	11.9	142	4	11.8	140	1.8%	3.40 [0.63, 6.17]
**Subtotal (95% CI)**			**2771**			**1839**	**15.2%**	**4.64 [4.00, 5.28]**
Heterogeneity: Tau² = 0.26; Chi² = 11.37, df = 7 (P = 0.12); I² = 38%								
Test for overall effect: Z = 14.25 (P < 0.00001)								
**5.1.3 Naltrexone/Bupropione**								
Apovian 2013	6.4	6.3	992	1.2	3.2	492	2.0%	5.20 [4.72, 5.68]
Greenway 2010	6.1	6.5	583	1.3	6.9	581	2.0%	4.80 [4.03, 5.57]
Hollander 2013	5	5.5	335	1.8	5.2	170	2.0%	3.20 [2.22, 4.18]
Wadden 2011	9.3	9.7	591	5.1	8.5	202	2.0%	4.20 [2.79, 5.61]
**Subtotal (95% CI)**			**2501**			**1445**	**7.9%**	**4.43 [3.54, 5.32]**
Heterogeneity: Tau² = 0.61; Chi² = 13.56, df = 3 (P = 0.004); I² = 78%								
Test for overall effect: Z = 9.76 (P < 0.00001)								
**5.1.4 Orlistat**								
Bakris 2022	5.4	6.4	267	2.7	6.4	265	2.0%	2.70 [1.61, 3.79]
Berne 2005	5	4.2	111	1.8	4.2	109	2.0%	3.20 [2.09, 4.31]
Davidson 1999	8.8	10.3	657	5.8	10.5	223	1.9%	3.00 [1.41, 4.59]
Derosa 2003	10.5	4	53	8.2	4	47	1.9%	2.30 [0.73, 3.87]
Finer 2000	8.8	4	110	5.5	4	108	2.0%	3.30 [2.24, 4.36]
Hauptman 2000	4.5	8.2	210	1.6	8.2	212	1.9%	2.90 [1.34, 4.46]
Hill 1999	8.2	5.3	179	6.4	7.7	184	2.0%	1.80 [0.44, 3.16]
Hollander 1998	6.2	5.7	162	4.3	6	159	2.0%	1.90 [0.62, 3.18]
James 1997	8.8	12.5	23	2	12.5	23	1.2%	6.80 [-0.42, 14.02]
Kelley 2002	3.8	5	266	1.2	5	269	2.0%	2.60 [1.75, 3.45]
Krempf 2003	7.4	13	346	4.8	11.2	350	1.9%	2.60 [0.80, 4.40]
Miles 2002	4.6	15.9	250	1.7	17	254	1.8%	2.90 [0.03, 5.77]
Poston 2003	8	12	37	0	12	35	1.5%	8.00 [2.45, 13.55]
Rossner 2000	9.7	6.3	244	7.6	4.8	243	2.0%	2.10 [1.11, 3.09]
Sjostrom 1998	10.3	4	343	6.1	1.7	340	2.0%	4.20 [3.74, 4.66]
Svendsen 2009	-2	7	23	-3	7	21	1.7%	1.00 [-3.14, 5.14]
Swinburn 2005	4.4	7.7	170	0.5	4.2	169	2.0%	3.90 [2.58, 5.22]
Togerson 2004	7	6	1640	4	6	1637	2.0%	3.00 [2.59, 3.41]
Zavoral 1998	9.2	6	1561	5.8	6	1119	2.0%	3.40 [2.94, 3.86]
**Subtotal (95% CI)**			**6652**			**5767**	**35.7%**	**2.99 [2.59, 3.38]**
Heterogeneity: Tau² = 0.33; Chi² = 43.19, df = 18 (P = 0.0008); I² = 58%								
Test for overall effect: Z = 14.92 (P < 0.00001)								
**5.1.5 Phentermine/Topiramate**								
Allison 2012	10.9	8.5	512	1.6	8.5	514	2.0%	9.30 [8.26, 10.34]
Gadde 2011	9.8	8.9	995	1.2	8.9	994	2.0%	8.60 [7.82, 9.38]
**Subtotal (95% CI)**			**1507**			**1508**	**4.0%**	**8.86 [8.20, 9.53]**
Heterogeneity: Tau² = 0.02; Chi² = 1.11, df = 1 (P = 0.29); I² = 10%								
Test for overall effect: Z = 26.15 (P < 0.00001)								
**5.1.6 POSE**								
Sullivan 2017	4.9	7	221	1.4	5.6	111	2.0%	3.50 [2.11, 4.89]
**Subtotal (95% CI)**			**221**			**111**	**2.0%**	**3.50 [2.11, 4.89]**
Heterogeneity: Not applicable								
Test for overall effect: Z = 4.93 (P < 0.00001)								
**5.1.7 Semaglutide**								
Bliddal 2024	13.7	3.3	271	3.2	3.3	136	2.0%	10.50 [9.82, 11.18]
Davies 2021	9.6	8	404	3.4	8	403	2.0%	6.20 [5.10, 7.30]
Garvey 2022	15.2	11.1	152	2.6	13.9	152	1.8%	12.60 [9.77, 15.43]
Kadowaki 2022	13.4	8.6	199	2.1	8	101	1.9%	11.30 [9.33, 13.27]
Kosiborod 2023	13.3	8.8	263	2.6	8.8	266	1.9%	10.70 [9.20, 12.20]
Kosiborod 2024	9.8	2	310	3.4	2	306	2.0%	6.40 [6.08, 6.72]
Lincoff 2023	10	9.6	8803	0.9	9.6	8801	2.0%	9.10 [8.82, 9.38]
McGowan, 2024	13.9	7.9	138	2.7	4.3	69	1.9%	11.20 [9.54, 12.86]
Rubino 2021	7.9	8.3	535	-6.9	8.8	268	2.0%	14.80 [13.53, 16.07]
Wadden 2021	17.6	2	407	5	3	204	2.0%	12.60 [12.14, 13.06]
Wilding 2021	14.9	3.6	1306	2.4	2.6	655	2.0%	12.50 [12.22, 12.78]
**Subtotal (95% CI)**			**12788**			**11361**	**21.5%**	**10.67 [8.95, 12.39]**
Heterogeneity: Tau² = 7.99; Chi² = 1082.18, df = 10 (P < 0.00001); I² = 99%								
Test for overall effect: Z = 12.18 (P < 0.00001)								
**5.1.8 Tirzepatide**								
Aronne, 2024	5.5	12.1	335	-14	11.2	335	1.9%	19.50 [17.73, 21.27]
Garvey 2023	13.8	8.8	623	3.3	8.8	315	2.0%	10.50 [9.31, 11.69]
Jastreboff 2022	20	4	1266	3.1	3	643	2.0%	16.90 [16.58, 17.22]
Loomba, 2024	14	4	95	-0.8	4	48	2.0%	14.80 [13.41, 16.19]
Malhotra, 2024	18.7	10.9	234	2	10.9	235	1.9%	16.70 [14.73, 18.67]
**Subtotal (95% CI)**			**2553**			**1576**	**9.8%**	**15.64 [12.89, 18.40]**
Heterogeneity: Tau² = 9.33; Chi² = 120.15, df = 4 (P < 0.00001); I² = 97%								
Test for overall effect: Z = 11.14 (P < 0.00001)								
**Total (95% CI)**			**29378**			**23935**	**100.0%**	**6.61 [5.29, 7.92]**
Heterogeneity: Tau² = 22.56; Chi² = 8039.37, df = 51 (P < 0.00001); I² = 99%								
Test for overall effect: Z = 9.84 (P < 0.00001)								
Test for subgroup differences: Chi² = 342.93, df = 7 (P < 0.00001), I² = 98.0%								

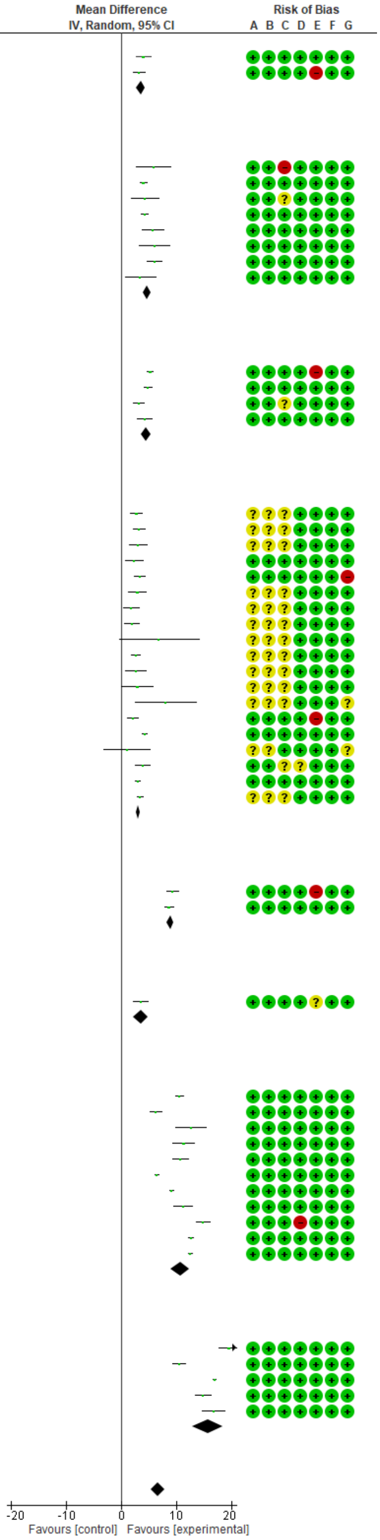

Risk of bias legend
(A) Random sequence generation (selection bias)
(B) Allocation concealment (selection bias)
(C) Blinding of participants and personnel (performance bias)
(D) Blinding of outcome assessment (detection bias)
(E) Incomplete outcome data (attrition bias)
(F) Selective reporting (reporting bias)
(G) Other bias

B

1) Liraglutide versus orlistat

Study or Subgroup	Liraglutide Mean	Liraglutide SD	Liraglutide Total	Orlistat Mean	Orlistat SD	Orlistat Total	Weight	Mean Difference IV, Random, 95% CI
Astrup 2012	7.5	10.6	93	3.7	10.9	95	100.0%	3.80 [0.73, 6.87]
**Total (95% CI)**			**93**			**95**	**100.0%**	**3.80 [0.73, 6.87]**

Heterogeneity: Not applicable

Test for overall effect: Z = 2.42 (P = 0.02)

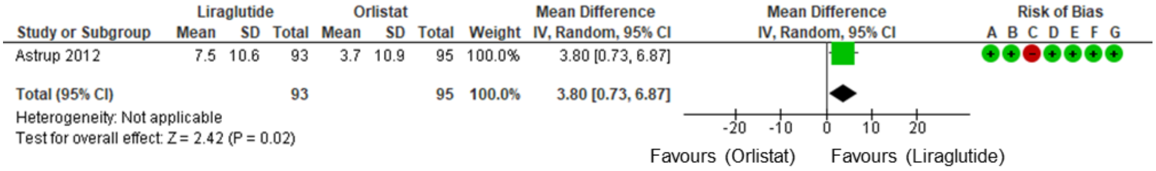

Risk of bias legend
(A) Random sequence generation (selection bias)
(B) Allocation concealment (selection bias)
(C) Blinding of participants and personnel (performance bias)
(D) Blinding of outcome assessment (detection bias)
(E) Incomplete outcome data (attrition bias)
(F) Selective reporting (reporting bias)
(G) Other bias

2) RYGB versus other metabolic bariatric surgery

Study or Subgroup	RYGB Mean	RYGB SD	RYGB Total	Other MBS Mean	Other MBS SD	Other MBS Total	Weight	Mean Difference IV, Random, 95% CI
**3.2.1 SG**								
Casajoana 2021	27.3	7.8	15	18	10.6	15	5.0%	9.30 [2.64, 15.96]
Catheline 2019	34.3	10.7	91	31.6	10.3	186	7.6%	2.70 [0.05, 5.35]
Karamanakos 2008	31.9	6.5	16	35.7	9.7	16	5.6%	-3.80 [-9.52, 1.92]
Keidar 2013	25.9	5.4	19	28.4	5.9	18	7.0%	-2.50 [-6.15, 1.15]
Pajecki 2023	30.3	7.9	18	17.2	8.8	18	5.8%	13.10 [7.64, 18.56]
Paluszkiewicz 2012	30.4	8.3	36	29.2	7.1	36	7.1%	1.20 [-2.37, 4.77]
Peterli 2018	28	7.5	110	26	7.5	107	8.0%	2.00 [0.00, 4.00]
Pullman 2023	26.2	12	56	13.4	12	58	6.5%	12.80 [8.39, 17.21]
Salminen 2022	26.9	10	49	23.4	10	52	6.9%	3.50 [-0.40, 7.40]
Schauer 2017	18.5	7.5	50	5	10.3	50	7.1%	13.50 [9.97, 17.03]
Svanevik 2023	30	6	54	23.7	4	55	8.0%	6.30 [4.38, 8.22]
Tang 2016	19	14.3	40	19	18.5	40	4.7%	0.00 [-7.25, 7.25]
Verrastro 2023	31.5	7.9	77	23.9	11.5	79	7.4%	7.60 [4.51, 10.69]
Wallenius 2020	26.9	8.9	25	20.4	9.1	24	6.1%	6.50 [1.46, 11.54]
Yang, 2015	31	7.1	32	27.1	7.1	32	7.1%	3.90 [0.42, 7.38]
**Subtotal (95% CI)**			**688**			**786**	**100.0%**	**5.06 [2.71, 7.42]**
Heterogeneity: Tau² = 17.14; Chi² = 92.81, df = 14 (P < 0.00001); I² = 85%								
Test for overall effect: Z = 4.21 (P < 0.0001)								
**3.2.2 OAGB**								
Delko 2024	32	6	40	37	6	40	28.9%	-5.00 [-7.63, -2.37]
Karagul 2024	36.6	10.2	20	35.3	5.3	22	22.0%	1.30 [-3.69, 6.29]
Robert 2024	37.1	10.3	117	35.4	8.1	117	29.6%	1.70 [-0.67, 4.07]
Singh 2023	26.6	10	24	24.9	10.9	25	19.5%	1.70 [-4.15, 7.55]
**Subtotal (95% CI)**			**201**			**204**	**100.0%**	**-0.33 [-4.37, 3.71]**
Heterogeneity: Tau² = 12.89; Chi² = 15.44, df = 3 (P = 0.001); I² = 81%								
Test for overall effect: Z = 0.16 (P = 0.87)								
**3.2.3 GCP**								
Casajoana 2021	27.3	7.8	15	14.8	7.8	15	100.0%	12.50 [6.92, 18.08]
**Subtotal (95% CI)**			**15**			**15**	**100.0%**	**12.50 [6.92, 18.08]**
Heterogeneity: Not applicable								
Test for overall effect: Z = 4.39 (P < 0.0001)								
**3.2.4 BPD**								
Hedberg 2012	16.2	4.9	23	23.2	6.9	24	34.1%	-7.00 [-10.41, -3.59]
Mingrone 2021	28.8	7.4	20	30.6	9.3	20	30.5%	-1.80 [-7.01, 3.41]
Salte 2024	20	5	31	33.9	5	29	35.4%	-13.90 [-16.43, -11.37]
**Subtotal (95% CI)**			**74**			**73**	**100.0%**	**-7.86 [-14.61, -1.11]**
Heterogeneity: Tau² = 31.82; Chi² = 21.58, df = 2 (P < 0.0001); I² = 91%								
Test for overall effect: Z = 2.28 (P = 0.02)								
**3.2.5 LAGB**								
Courcolas 2020	25	91	20	12.7	8.5	21	0.8%	12.30 [-27.75, 52.35]
Nguyen 2018	32.7	13.6	111	21.2	11.9	86	99.2%	11.50 [7.93, 15.07]
**Subtotal (95% CI)**			**131**			**107**	**100.0%**	**11.51 [7.95, 15.06]**
Heterogeneity: Tau² = 0.00; Chi² = 0.00, df = 1 (P = 0.97); I² = 0%								
Test for overall effect: Z = 6.35 (P < 0.00001)								
**3.2.6 LVGB**								
Werling 2013	25.8	8	37	20.7	8	46	100.0%	5.10 [1.64, 8.56]
**Subtotal (95% CI)**			**37**			**46**	**100.0%**	**5.10 [1.64, 8.56]**
Heterogeneity: Not applicable								
Test for overall effect: Z = 2.89 (P = 0.004)								

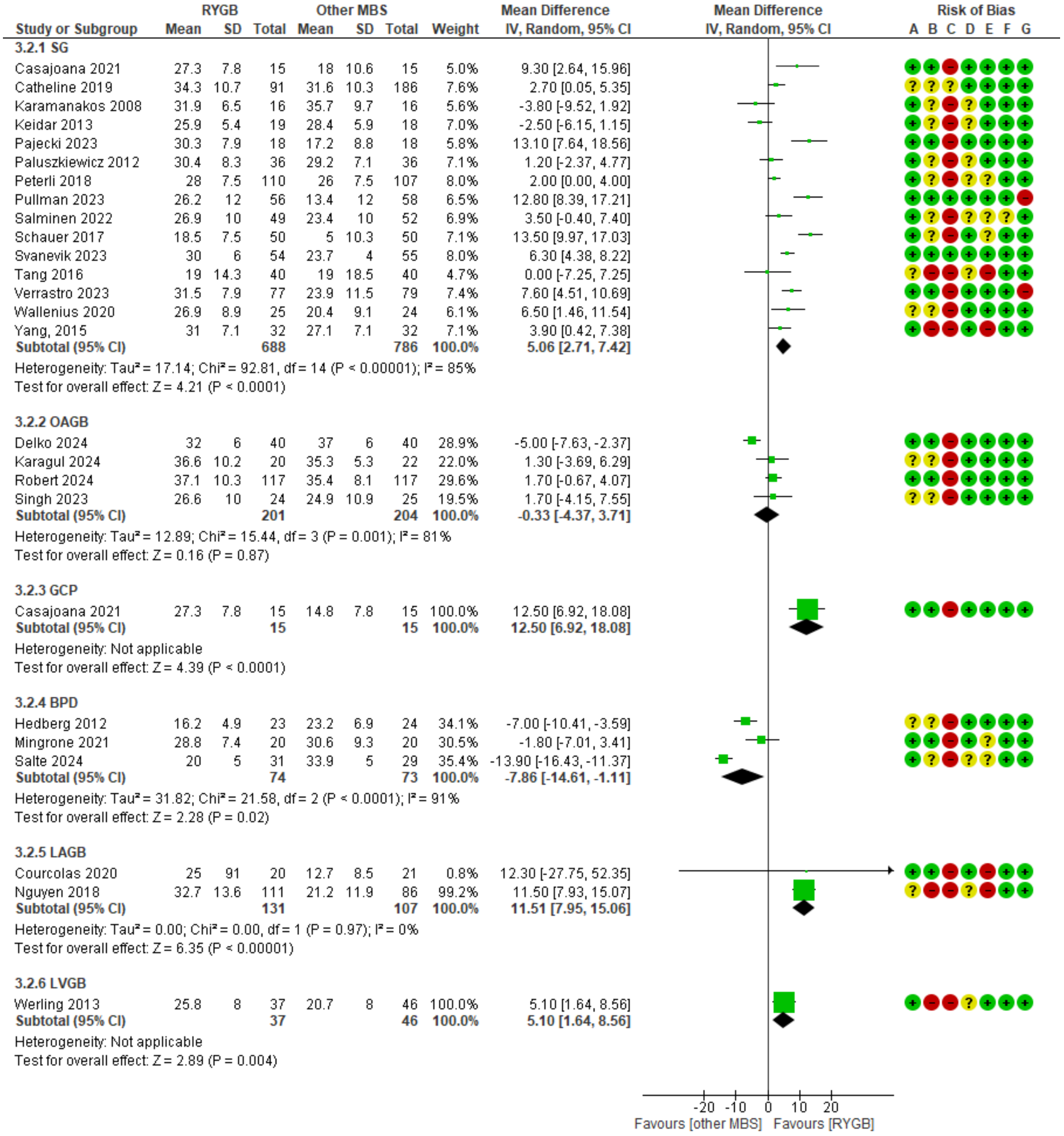

Risk of bias legend
(A) Random sequence generation (selection bias)
(B) Allocation concealment (selection bias)
(C) Blinding of participants and personnel (performance bias)
(D) Blinding of outcome assessment (detection bias)
(E) Incomplete outcome data (attrition bias)
(F) Selective reporting (reporting bias)
(G) Other bias

3) OAGB versus other metabolic bariatric surgery

Study or Subgroup	OAGB Mean	OAGB SD	OAGB Total	other MBS Mean	other MBS SD	other MBS Total	Weight	Mean Difference IV, Random, 95% CI	A	B	C	D	E	F	G
**3.4.1 SG**															
Hany 2024	40.8	14.7	150	29.2	25	150	29.6%	11.60 [6.96, 16.24]							
Lee 2014	22.8	5.9	30	20.1	5.3	30	36.2%	2.70 [-0.14, 5.54]	+	?	?	+	−	+	+
Roushdy 2020	47.1	5.1	21	42.9	6.1	21	34.2%	4.20 [0.80, 7.60]							
**Subtotal (95% CI)**			**201**			**201**	**100.0%**	**5.85 [1.15, 10.55]**							
Heterogeneity: Tau² = 13.81; Chi² = 10.47, df = 2 (P = 0.005); I² = 81%															
Test for overall effect: Z = 2.44 (P = 0.01)															
**3.4.2 GCP**															
Darabi 2013	32.4	9	20	30	8.2	20	100.0%	2.40 [-2.94, 7.74]							
**Subtotal (95% CI)**			**20**			**20**	**100.0%**	**2.40 [-2.94, 7.74]**							
Heterogeneity: Not applicable															
Test for overall effect: Z = 0.88 (P = 0.38)															

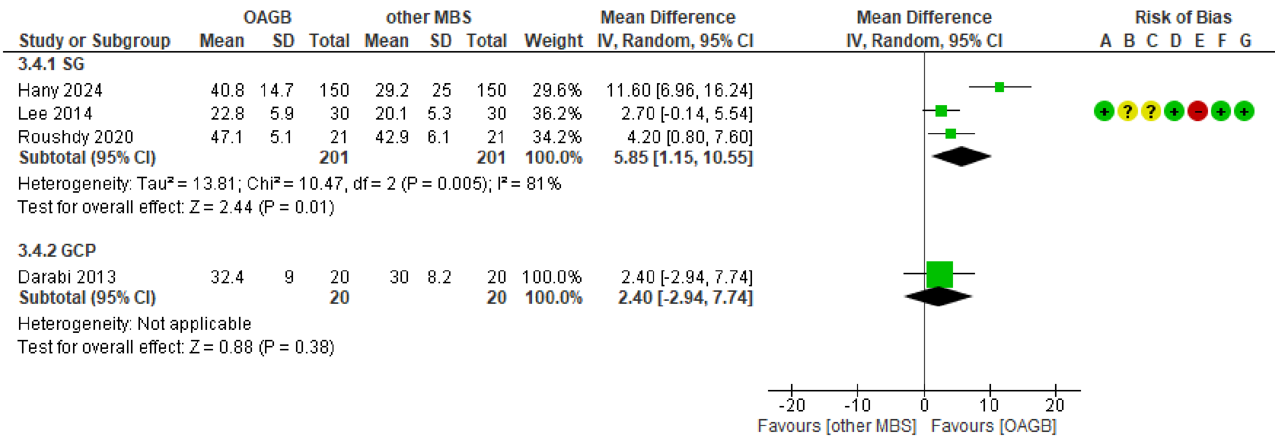

Risk of bias legend
(A) Random sequence generation (selection bias)
(B) Allocation concealment (selection bias)
(C) Blinding of participants and personnel (performance bias)
(D) Blinding of outcome assessment (detection bias)
(E) Incomplete outcome data (attrition bias)
(F) Selective reporting (reporting bias)
(G) Other bias

4) SG versus other metabolic bariatric surgery

Study or Subgroup	SG Mean	SG SD	SG Total	GCP Mean	GCP SD	GCP Total	Weight	Mean Difference IV, Random, 95% CI	A	B	C	D	E	F	G
Casajoana 2021	18	10.6	15	14.8	7.8	15	17.6%	3.20 [-3.46, 9.86]	+	+	−	+	+	+	+
Talebpour 2018	38	7	35	34	6.1	35	82.4%	4.00 [0.92, 7.08]	+	+	−	+	+	+	+
**Total (95% CI)**			**50**			**50**	**100.0%**	**3.86 [1.07, 6.65]**							
Heterogeneity: Tau² = 0.00; Chi² = 0.05, df = 1 (P = 0.83); I² = 0%															
Test for overall effect: Z = 2.71 (P = 0.007)															

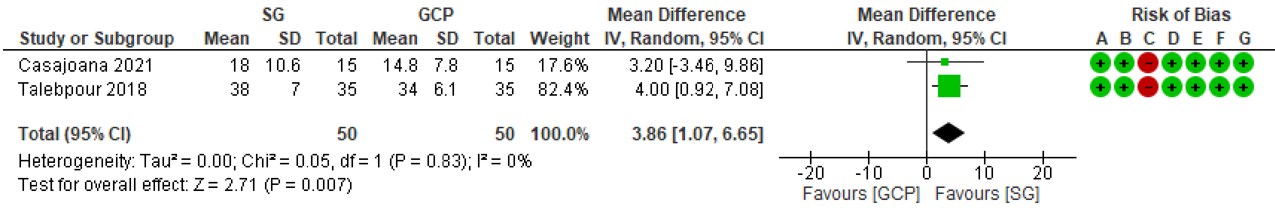

Risk of bias legend
(A) Random sequence generation (selection bias)
(B) Allocation concealment (selection bias)
(C) Blinding of participants and personnel (performance bias)
(D) Blinding of outcome assessment (detection bias)
(E) Incomplete outcome data (attrition bias)
(F) Selective reporting (reporting bias)
(G) Other bias

5) SADI versus other metabolic bariatric surgery

Study or Subgroup	SADI Mean	SADI SD	SADI Total	BPD Mean	BPD SD	BPD Total	Weight	Mean Difference IV, Random, 95% CI	A	B	C	D	E	F	G
Axer 2024	40.1	5.9	30	41.6	6.4	30	100.0%	-1.50 [-4.61, 1.61]							
**Total (95% CI)**			**30**			**30**	**100.0%**	**-1.50 [-4.61, 1.61]**							
Heterogeneity: Not applicable															
Test for overall effect: Z = 0.94 (P = 0.35)															

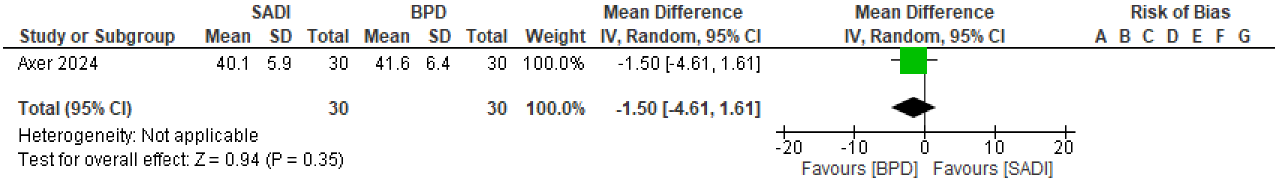

Risk of bias legend
(A) Random sequence generation (selection bias)
(B) Allocation concealment (selection bias)
(C) Blinding of participants and personnel (performance bias)
(D) Blinding of outcome assessment (detection bias)
(E) Incomplete outcome data (attrition bias)
(F) Selective reporting (reporting bias)
(G) Other bias

LAGB: Laparoscopic Adjustable Gastric Banding; **GCP**: Greater Curvature Plication Gastric; **LVGB**: Laparoscopic Vertical Banded Gastroplasty; **SG**: Sleeve Gastrectomy; **OAGB**: One-anastomosis gastric bypass; **RYGB**: Roux-en-Y Gastric By-Pass; **SADI**: Single Anastomosis Duodenal Switch; **BPD**: Bilio-Pancreatic Diversion

Figure 5S – Funnel plots for TBWL% at 26-52 (Panel A), 53-104 (Panel B), 105-156 (Panel C), >156 (Panel D) weeks.

A

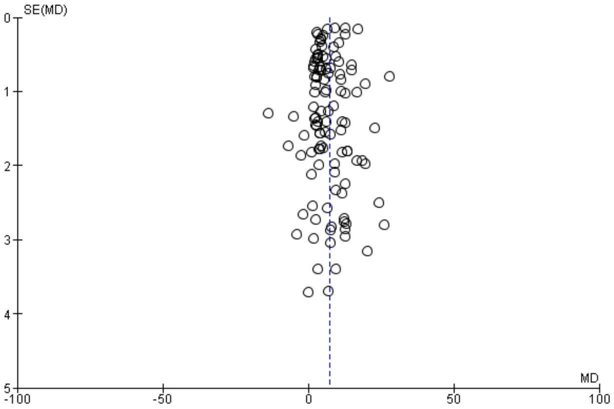

B

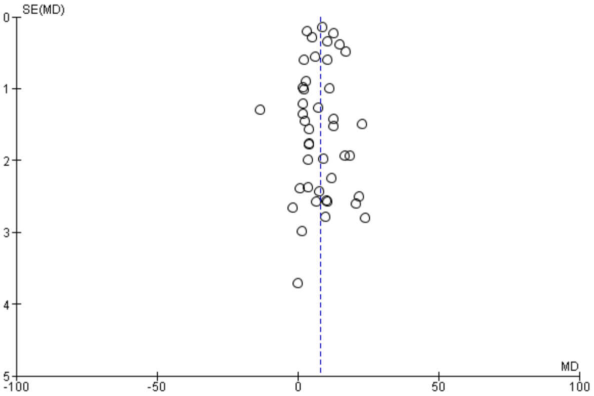

C

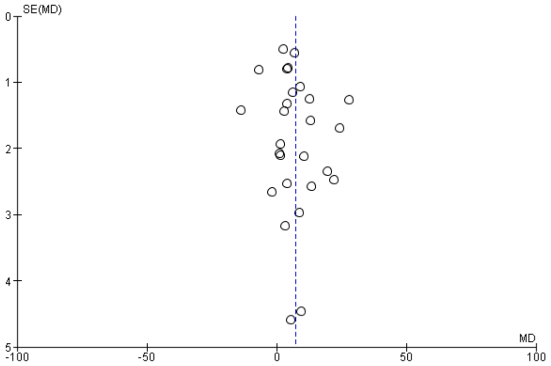

D

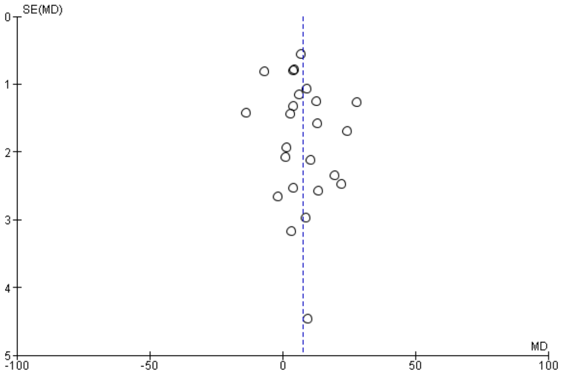

Figure 6S – Network meta-analysis for different anti-obesity strategies. Network plot for TBWL% at 26-52 weeks (RCTs with a mean BMI at entry: Panel A: 30-34.9 Kg/m^2; B 35-39.9 kg/m^2; C: > 39.9 kg/m^2). For all analyses H values were <1.1 meaning no relevant inconsistency (H<3 indicates no relevant inconsistency of treatment effects).

A

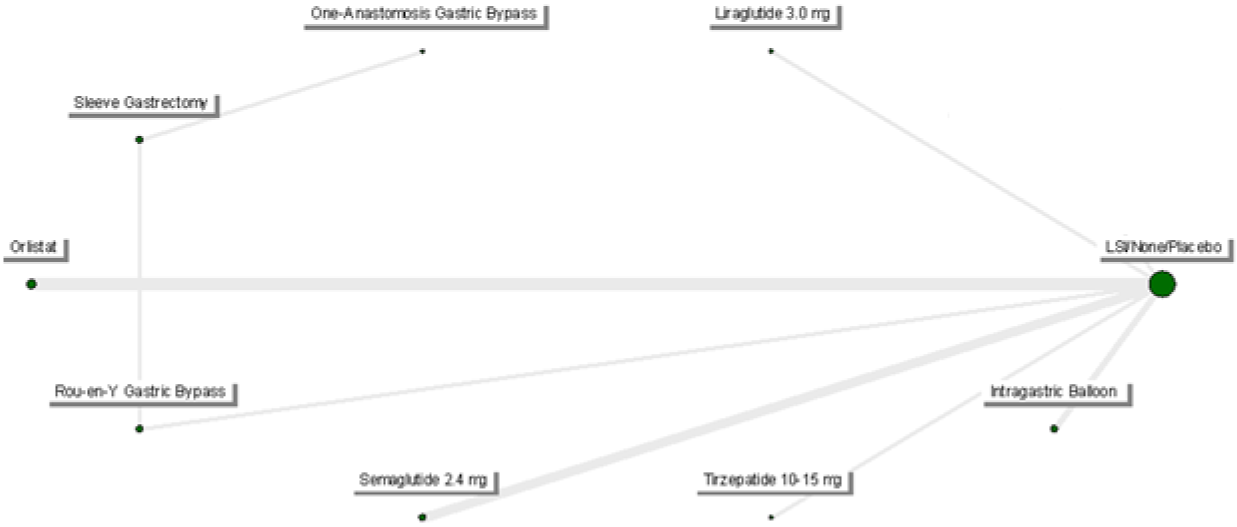

B

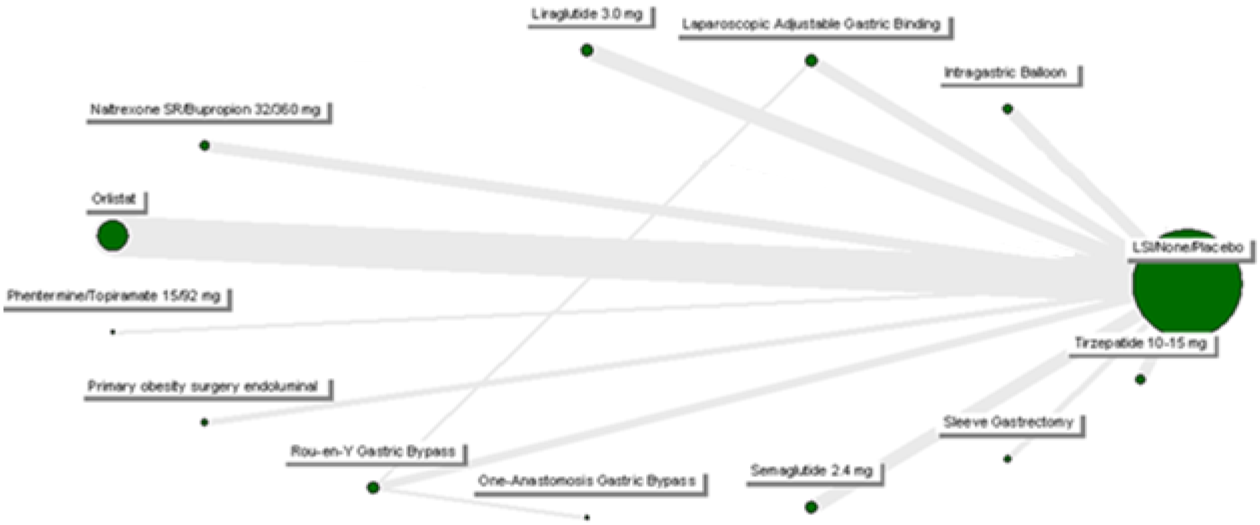

C

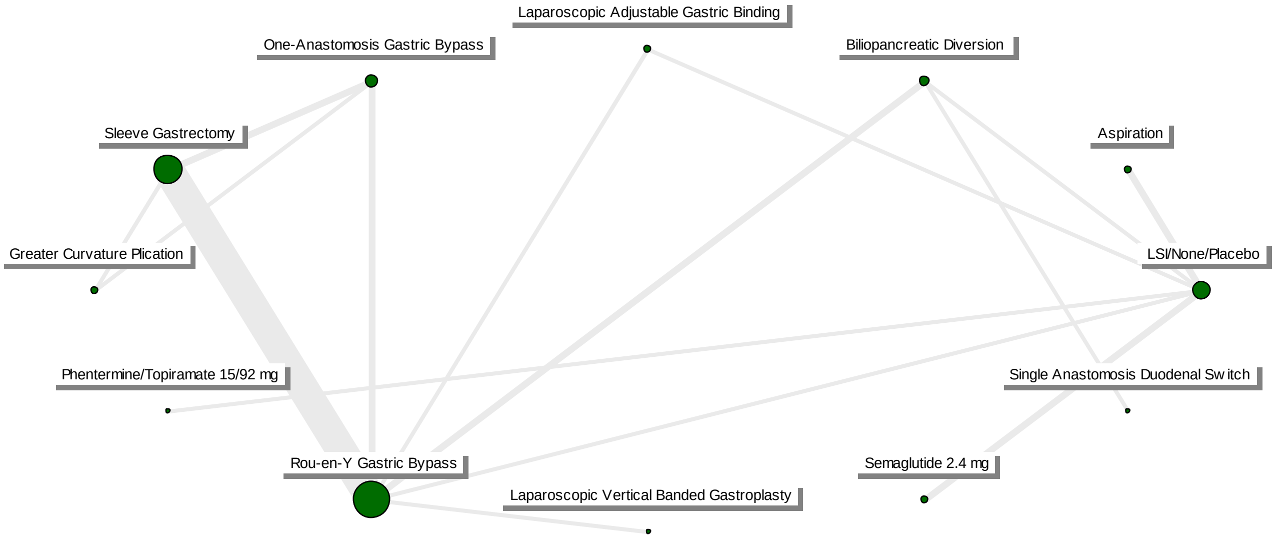

Figure 7S – Network meta-analysis for different anti-obesity strategies. Forest plot for TBWL% at 26-52 weeks (RCTs with a mean BMI at entry: Panel A: 30-34.9 kg/m^2; B 35-39.9 kg/m^2; C: > 39.9 kg/m^2). For all analyses H values were <1.1 meaning no relevant inconsistency (H<3 indicates no relevant inconsistency of treatment effects).

A

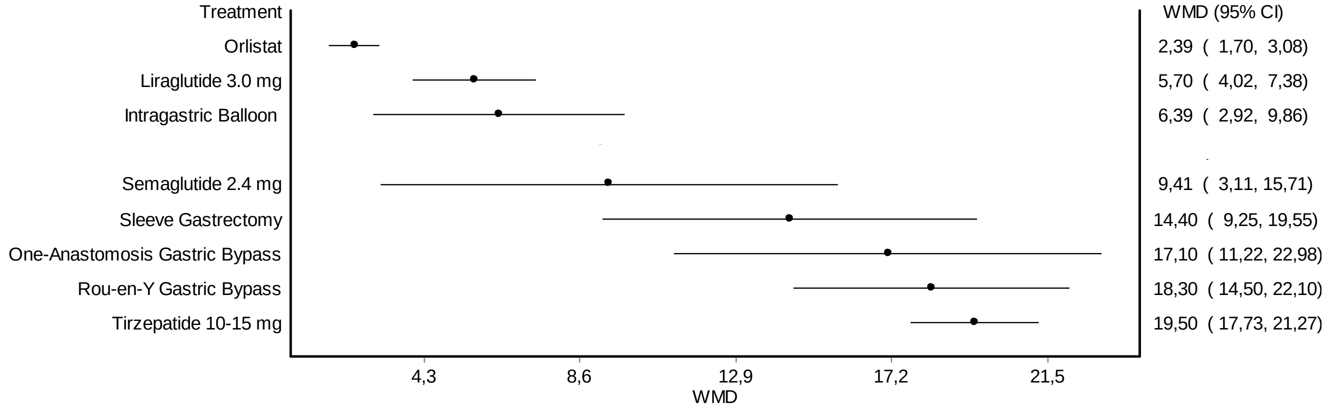

B

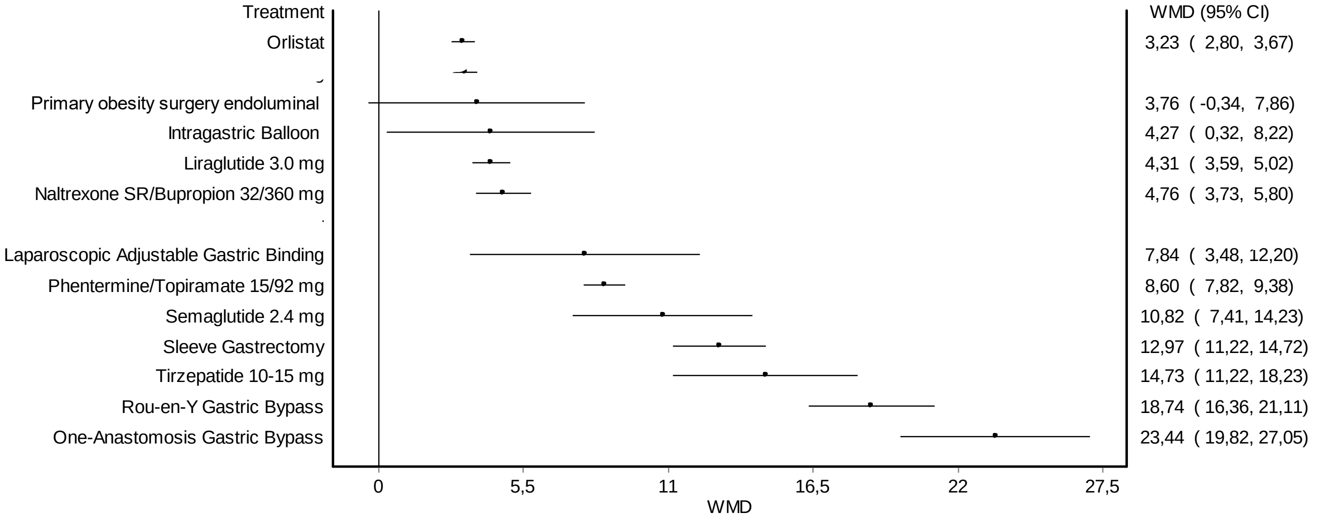

C

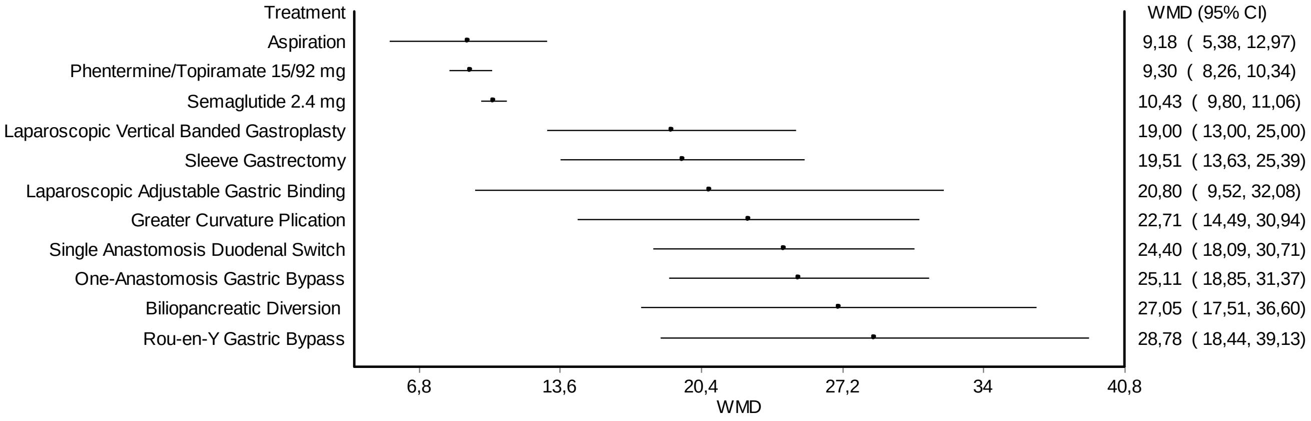
Treatment
Aspiration
Phentermine/Topiramate 15/92 mg
Semaglutide 2.4 mg
Laparoscopic Vertical Banded Gastroplasty
Sleeve Gastrectomy
Laparoscopic Adjustable Gastric Binding
Greater Curvature Plication
Single Anastomosis Duodenal Switch
One-Anastomosis Gastric Bypass
Biliopancreatic Diversion
Rou-en-Y Gastric Bypass
WMD (95% CI)
9,18 (5,38, 12,97)
9,30 (8,26, 10,34)
10,43 (9,80, 11,06)
19,00 (13,00, 25,00)
19,51 (13,63, 25,39)
20,80 (9,52, 32,08)
22,71 (14,49, 30,94)
24,40 (18,09, 30,71)
25,11 (18,85, 31,37)
27,05 (17,51, 36,60)
28,78 (18,44, 39,13)
6,8
13,6
20,4
27,2
34
40,8
WMD

Figure 8S – Effects of different anti-obesity strategies on TBWL% at 53-104 weeks in RCTs with a mean BMI at entry 30-34.9 kg/m^2. Panel A: Interventions versus Placebo/LSI; Panel B: Active comparisons). SG: Sleeve Gastrectomy; OAGB: One-anastomosis gastric bypass; RYGB: Roux-en-Y Gastric By-Pass. Heterogeneity was assessed using I2 statistics and a value≥50% means a high grade of heterogeneity.

A

Study or Subgroup	Intervention Mean	Intervention SD	Intervention Total	Placebo/LSI Mean	Placebo/LSI SD	Placebo/LSI Total	Weight	Mean Difference IV, Random, 95% CI
**1.3.1 Semaglutide**								
Lincoff, 2023	9	9.6	8803	3	9.6	8801	33.7%	6.00 [5.72, 6.28]
Kadowaki, 2022	13.4	8.6	199	2.1	8	101	32.8%	11.30 [9.33, 13.27]
Rubino, 2021	7.9	8.3	535	-6.9	2.2	268	33.6%	14.80 [14.05, 15.55]
**Subtotal (95% CI)**			**9537**			**9170**	**100.0%**	**10.69 [3.97, 17.41]**
Heterogeneity: Tau² = 34.90; Chi² = 478.84, df = 2 (P < 0.00001); I² = 100%								
Test for overall effect: Z = 3.12 (P = 0.002)								
**1.3.2 RYGB**								
Ikramuddin, 2018	21.1	13.9	57	9.8	14.9	56	100.0%	11.30 [5.98, 16.62]
**Subtotal (95% CI)**			**57**			**56**	**100.0%**	**11.30 [5.98, 16.62]**
Heterogeneity: Not applicable								
Test for overall effect: Z = 4.17 (P < 0.0001)								

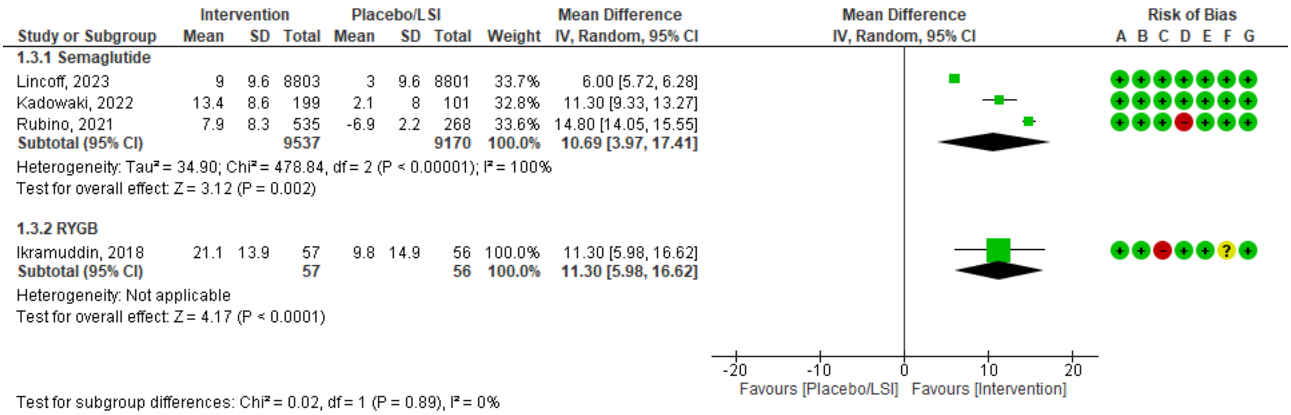

Test for subgroup differences: Chi² = 0.02, df = 1 (P = 0.89), I² = 0%
Risk of bias legend
(A) Random sequence generation (selection bias)
(B) Allocation concealment (selection bias)
(C) Blinding of participants and personnel (performance bias)
(D) Blinding of outcome assessment (detection bias)
(E) Incomplete outcome data (attrition bias)
(F) Selective reporting (reporting bias)
(G) Other bias

B

Study or Subgroup	Intervention Mean	Intervention SD	Intervention Total	Sleeve Gastrectomyl Mean	Sleeve Gastrectomyl SD	Sleeve Gastrectomyl Total	Weight	Mean Difference IV, Random, 95% CI
**1.4.1 RYGB**								
Yang, 2015	31.8	7.1	32	27.7	7.1	32	100.0%	4.10 [0.62, 7.58]
**Subtotal (95% CI)**			**32**			**32**	**100.0%**	**4.10 [0.62, 7.58]**
Heterogeneity: Not applicable								
Test for overall effect: Z = 2.31 (P = 0.02)								
**1.4.2 OAGB**								
Lee, 2014	22.7	5.9	30	20.3	5.3	30	100.0%	2.40 [-0.44, 5.24]
**Subtotal (95% CI)**			**30**			**30**	**100.0%**	**2.40 [-0.44, 5.24]**
Heterogeneity: Not applicable								
Test for overall effect: Z = 1.66 (P = 0.10)								

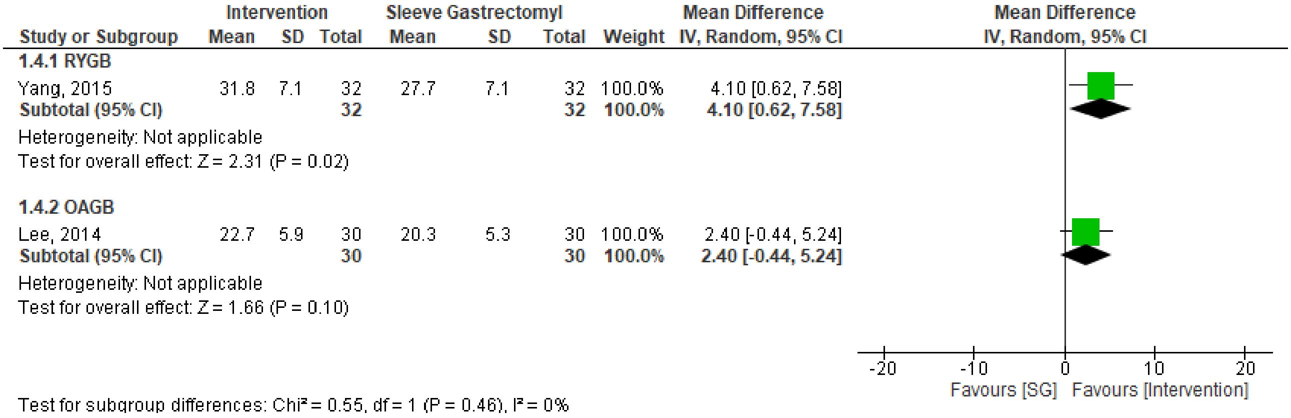

Test for subgroup differences: Chi² = 0.55, df = 1 (P = 0.46), I² = 0%

Figure 9S – Network meta-analysis for different anti-obesity strategies. Network plot for TBWL% at 53-104 weeks (RCTs with a mean BMI at entry: Panel A: 35-39.9 kg/m^2; B: > 39.9 kg/m^2). For all analyses H values were <1.1 meaning no relevant inconsistency (H<3 indicates no relevant inconsistency of treatment effects).

A

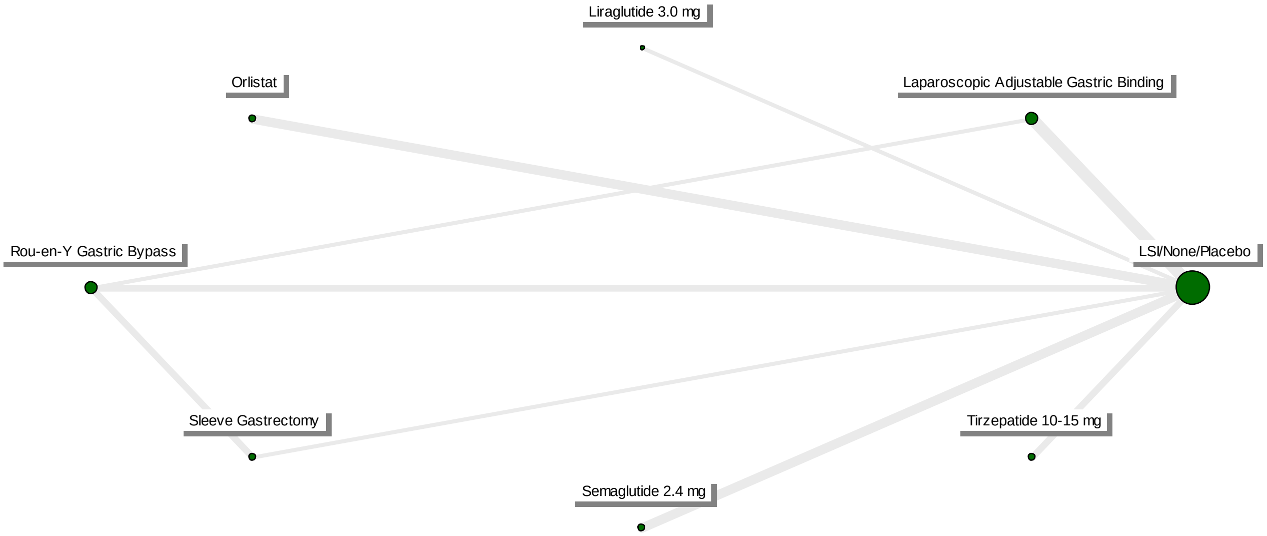

B

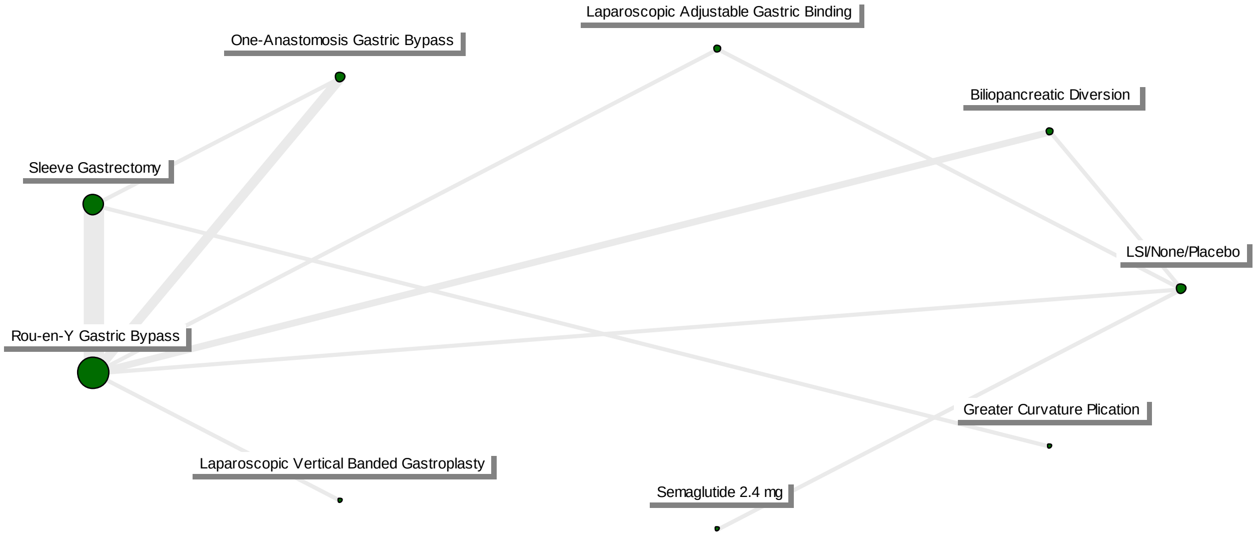

Figure 10S – Network meta-analysis for different anti-obesity strategies. Forest plot for TBWL% at 53-104 weeks (RCTs with a mean BMI at entry: Panel A: 35-39.9 kg/m^2; B: > 39.9 kg/m^2).

A

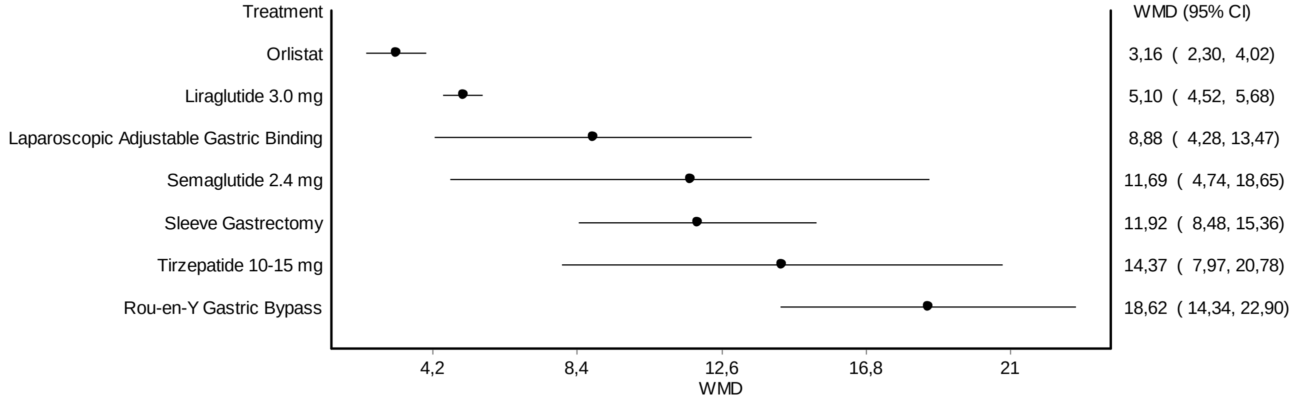

B

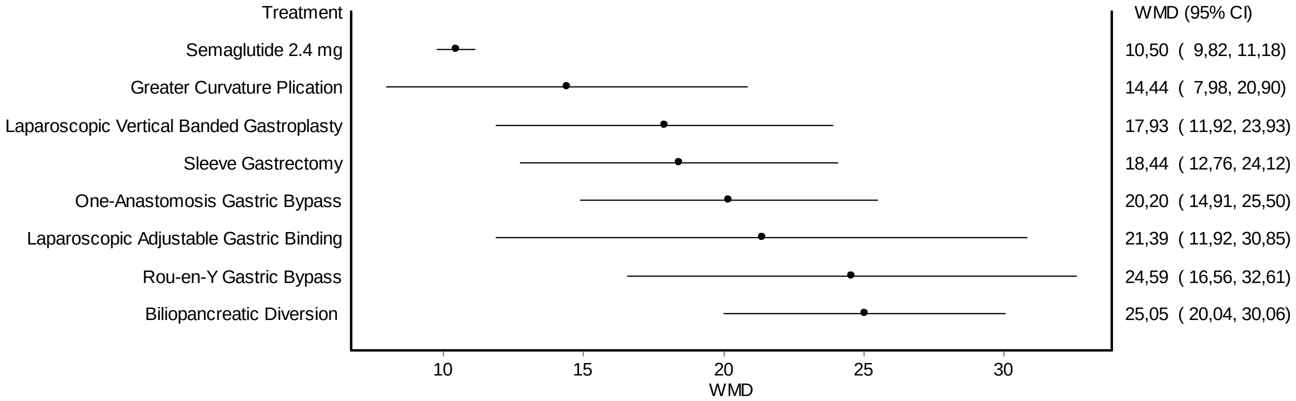

Figure 11S – Network meta-analysis for different anti-obesity strategies. Network plot for TBWL% at 105-156 weeks (RCTs with a mean BMI at entry > 39.9 kg/m^2). For all analyses H values were <1.1 meaning no relevant inconsistency (H<3 indicates no relevant inconsistency of treatment effects).

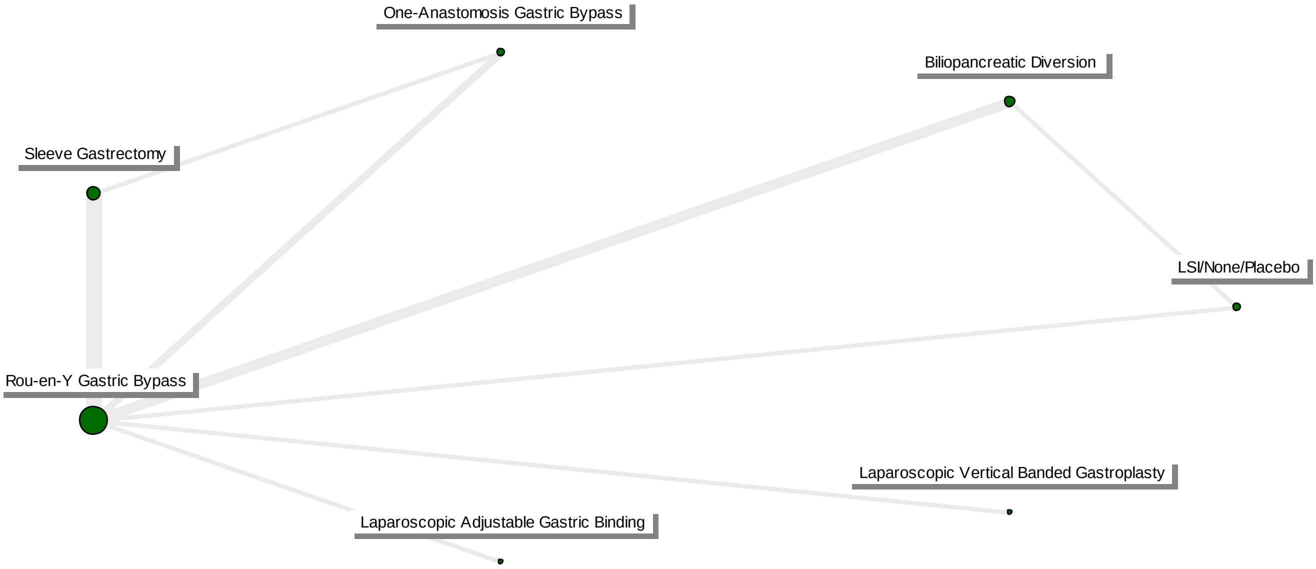

Figure 12S – Network meta-analysis for different anti-obesity strategies. Forest plot for TBWL% at 105-156 weeks (RCTs with a mean BMI at entry > 39.9 kg/m^2).

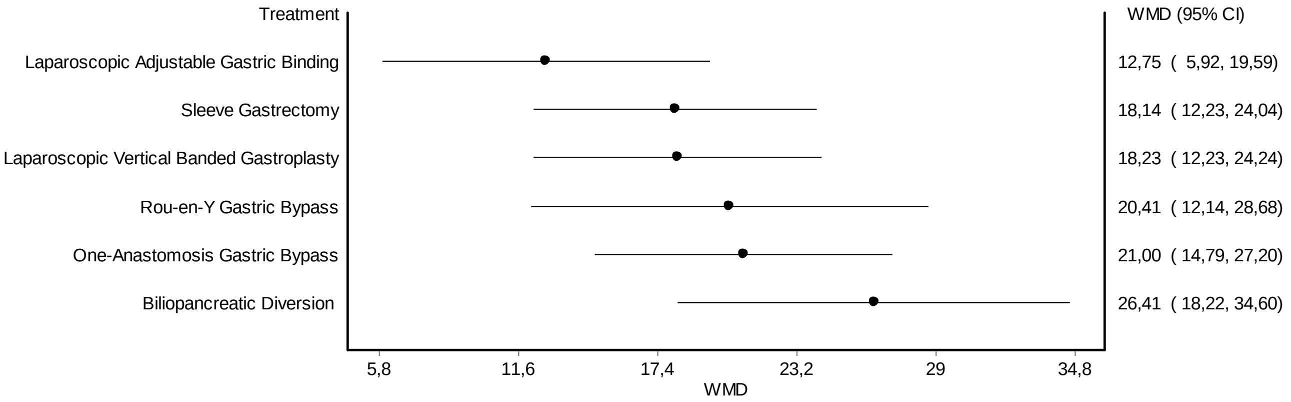

Figure 13S – Network meta-analysis for different anti-obesity strategies. Network plot for TBWL% at 157-520 weeks (RCTs with a mean BMI at entry > 39.9 kg/m^2). For all analyses H values were <1.1 meaning no relevant inconsistency (H<3 indicates no relevant inconsistency of treatment effects).

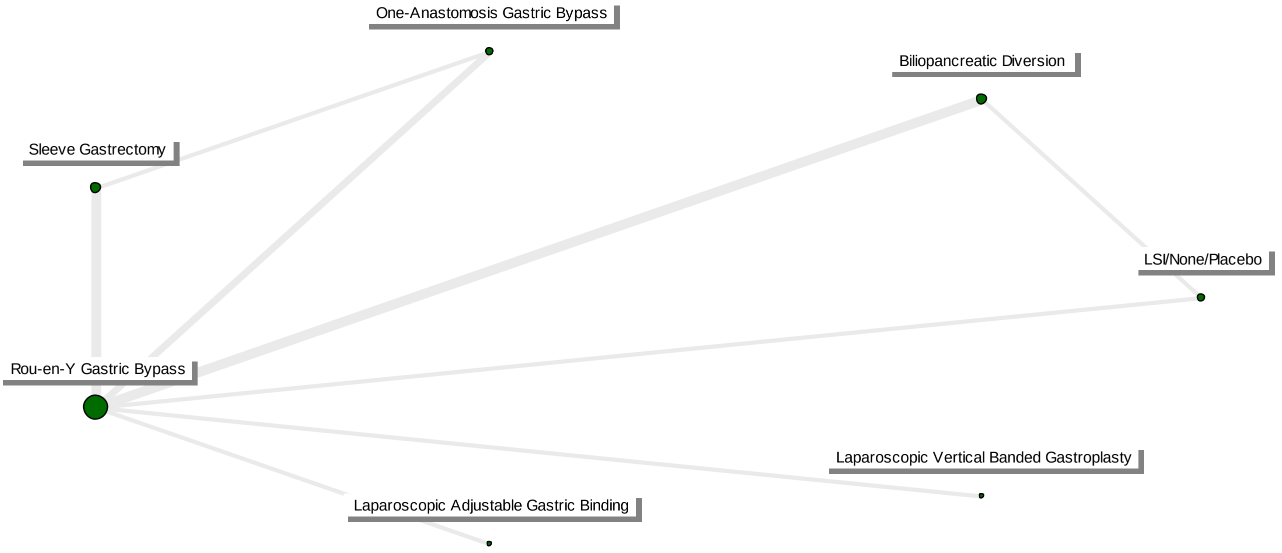

Figure 14S – Network meta-analysis for different anti-obesity strategies. Forest plot for TBWL% at 157-520 weeks (RCTs with a mean BMI at entry > 39.9 kg/m^2).

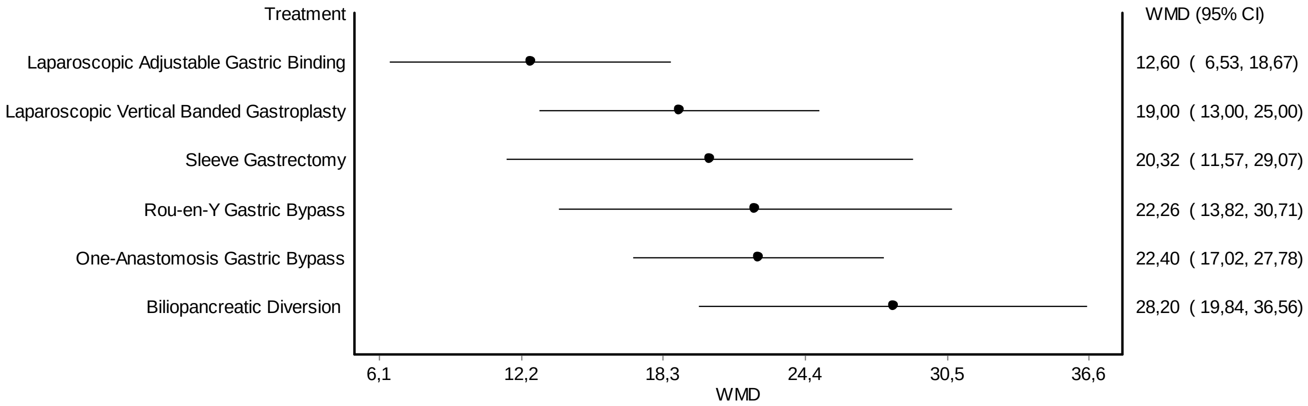

Figure 15S – Network meta-analysis for different anti-obesity strategies. Network plot for TBWL% at 26-52 weeks. RCTs enrolling (panel A) and non-enrolling (Panel B) subjects with diabetes mellitus. For all analyses H values were <1.1 meaning no relevant inconsistency (H<3 indicates no relevant inconsistency of treatment effects).

A

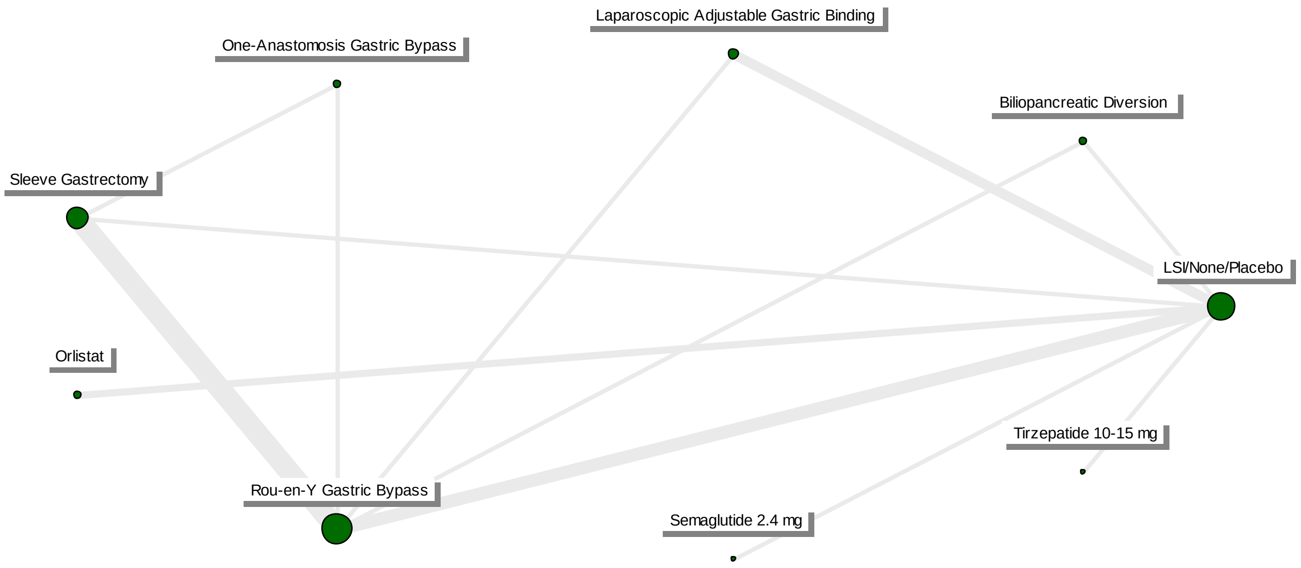

B

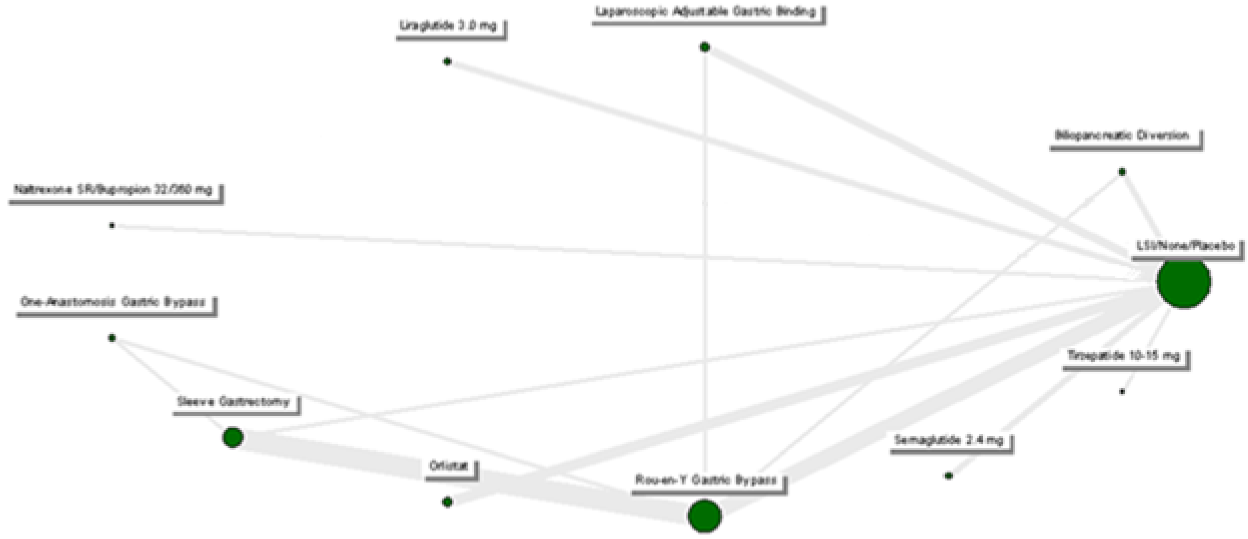

Figure 16S – Network meta-analysis for different anti-obesity strategies. Forest plot for TBWL% at 26-52 weeks. RCTs enrolling (panel A) and non-enrolling (Panel B) subjects with diabetes mellitus.

A

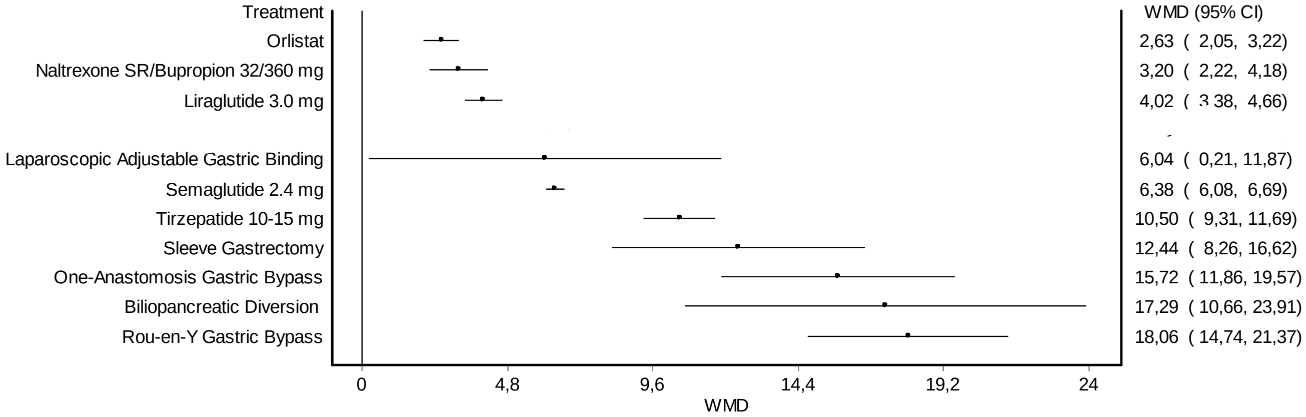

B

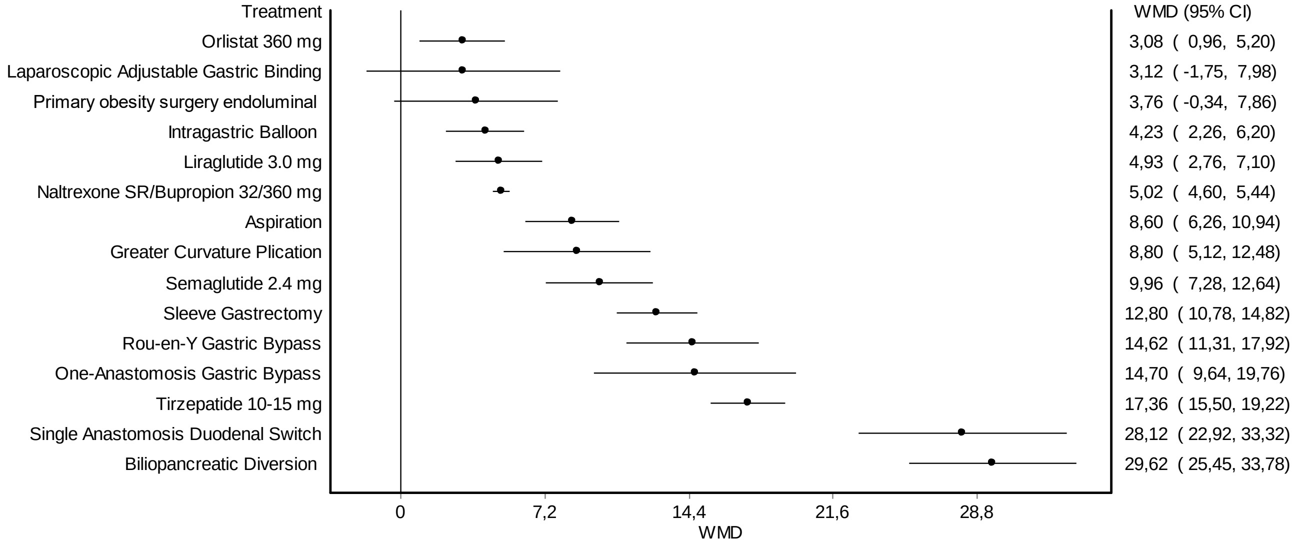

Figure 17S – Network meta-analysis for different anti-obesity strategies. Network plot for TBWL% at 53-104 weeks. RCTs enrolling (panel A) and non-enrolling (Panel B) subjects with diabetes mellitus. For all analyses H values were <1.1 meaning no relevant inconsistency (H<3 indicates no relevant inconsistency of treatment effects).

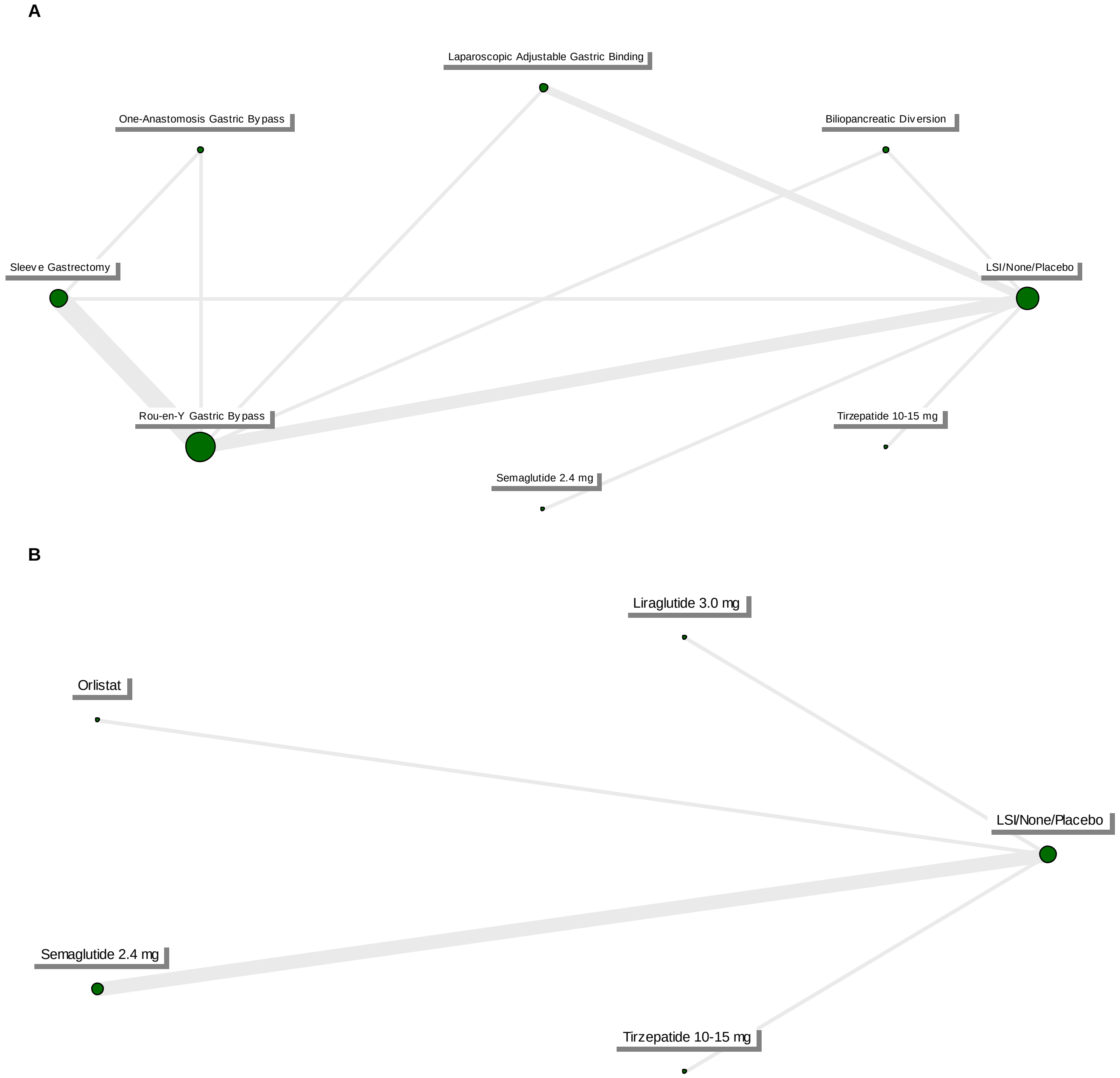

Figure 18S – Network meta-analysis for different anti-obesity strategies. Forest plot for TBWL% at 53-104 weeks. RCTs enrolling (panel A) and non-enrolling (Panel B) subjects with diabetes mellitus.

A

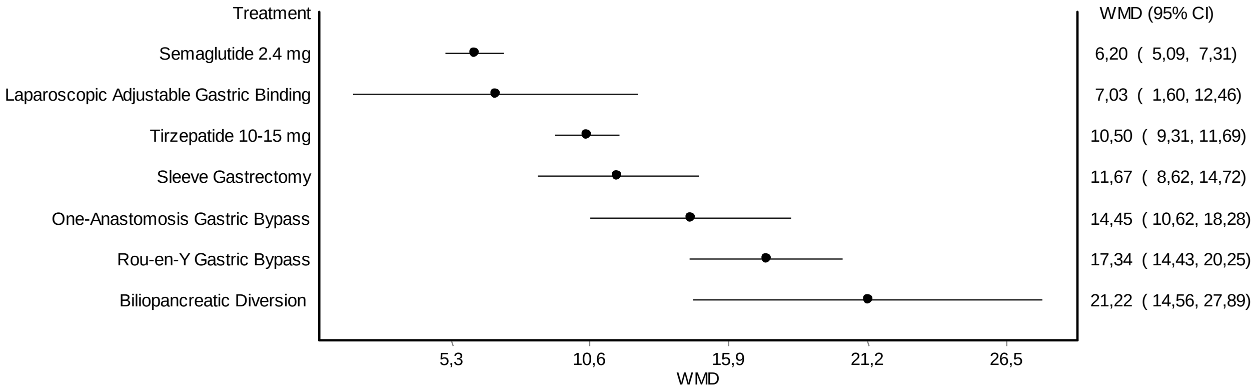

B

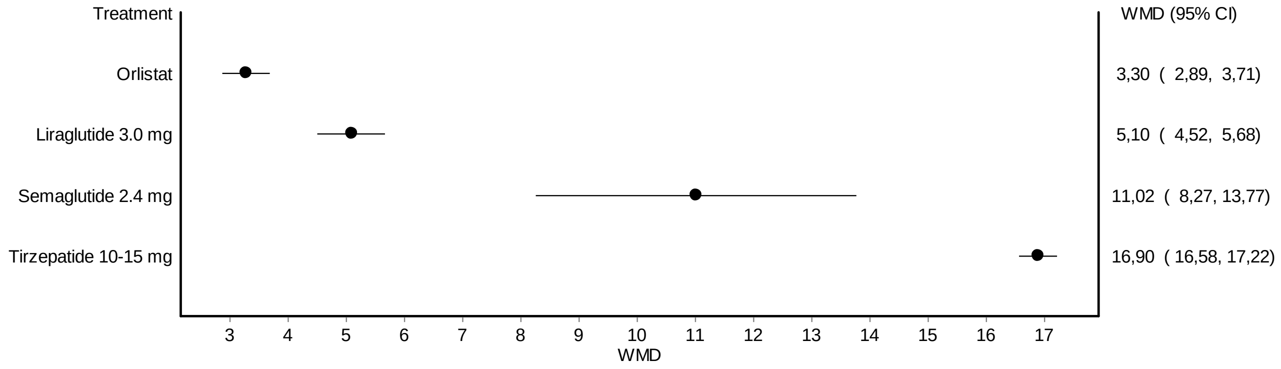

Figure 19S – Network meta-analysis for different anti-obesity strategies. Network plot for TBWL% at 105-156 weeks. RCTs enrolling (panel A) and non-enrolling (Panel B) subjects with diabetes mellitus. For all analyses H values were <1.1 meaning no relevant inconsistency (H<3 indicates no relevant inconsistency of treatment effects).

A

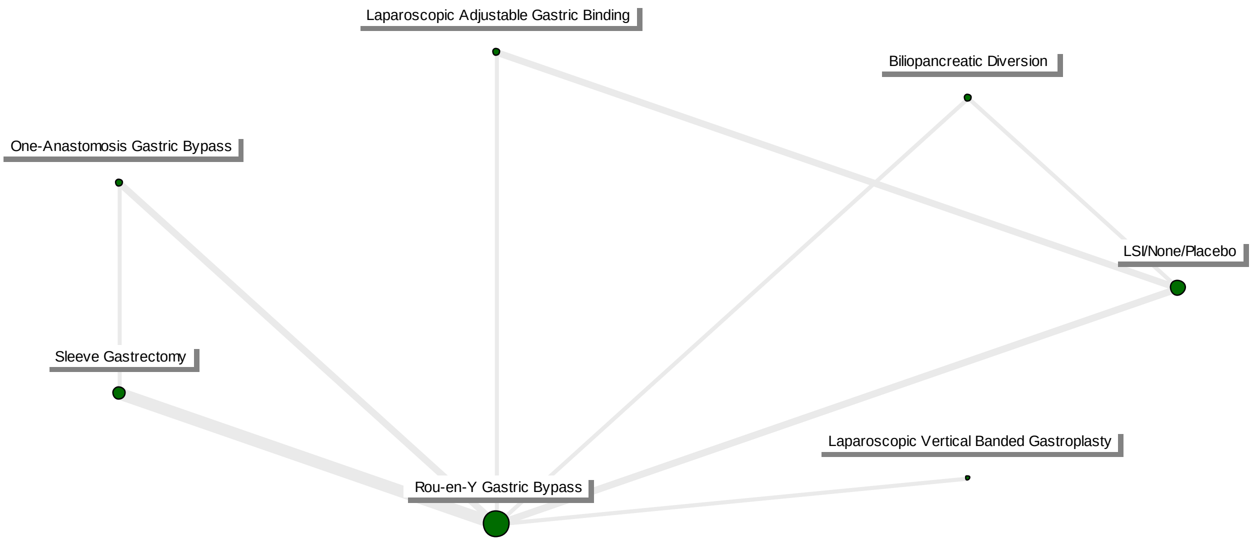

B

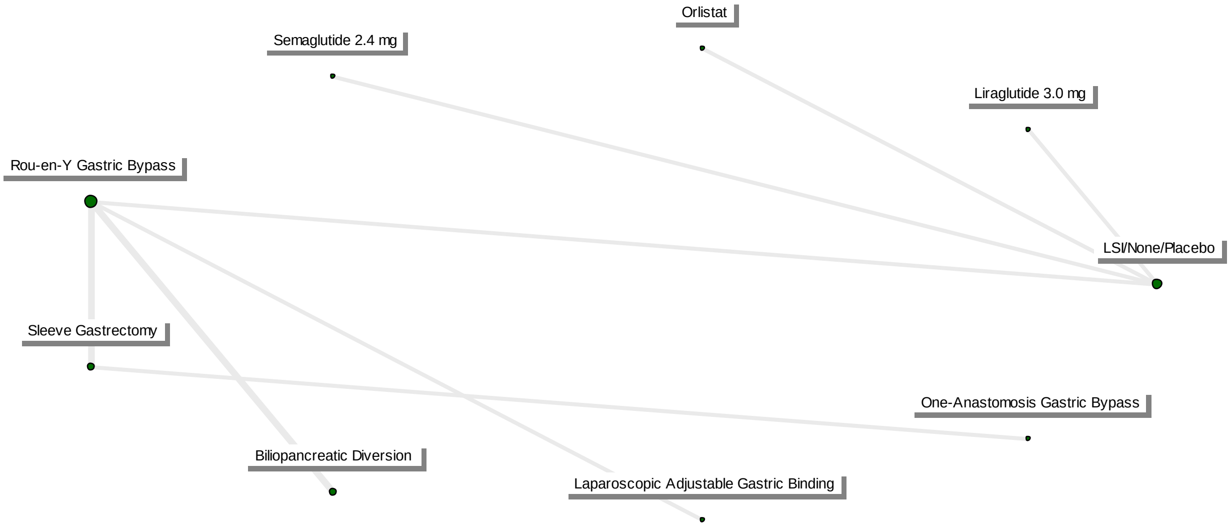

Figure 20S – Network meta-analysis for different anti-obesity strategies. Forest plot for TBWL% at 105-156 weeks. RCTs enrolling (panel A) and non-enrolling (Panel B) subjects with diabetes mellitus.

A

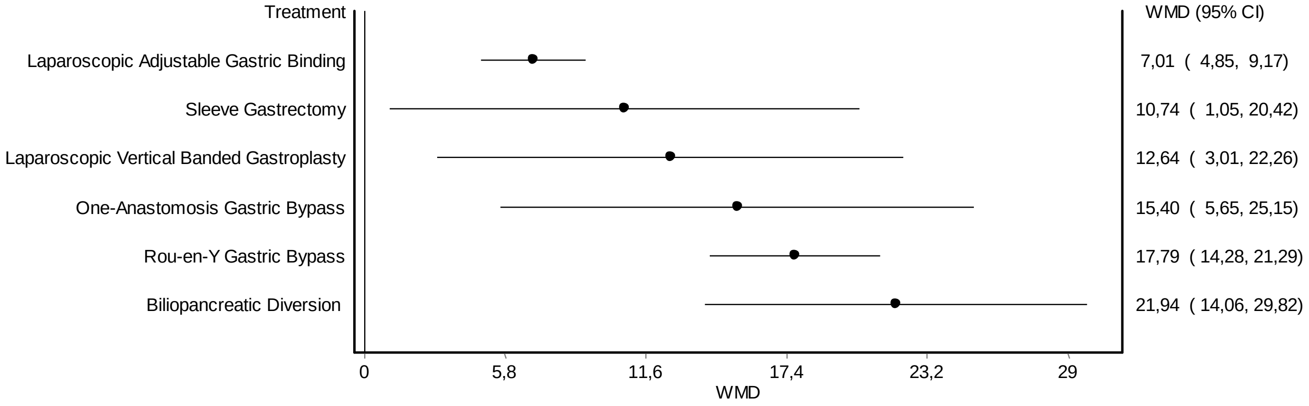

B

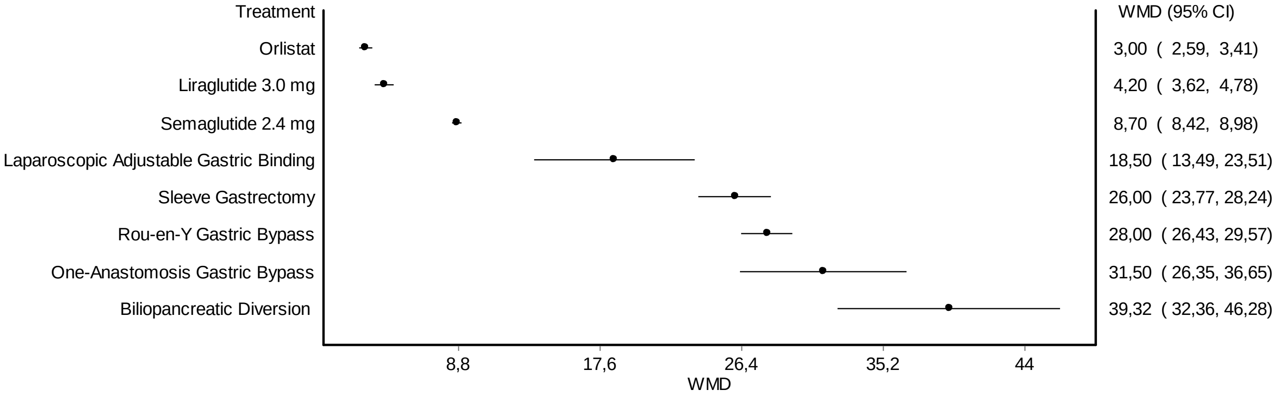

Figure 21S – Network meta-analysis for different anti-obesity strategies. Network plot for TBWL% at 157-520 weeks. RCTs non-enrolling subjects with diabetes mellitus. RCT enrolling diabetes are insufficient (n=7) to perform a formal NMA. For all analyses H values were <1.1 meaning no relevant inconsistency (H<3 indicates no relevant inconsistency of treatment effects).

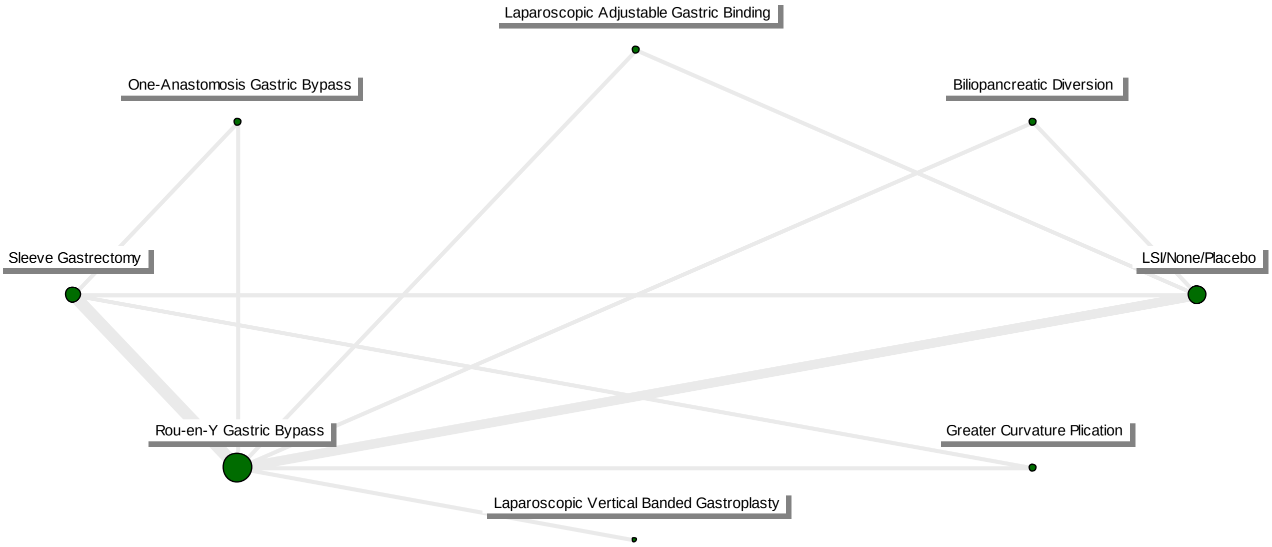

Figure 22S – Network meta-analysis for different anti-obesity strategies. Forest plot for TBWL% at 157-520 weeks. RCTs enrolling (panel A) and non-enrolling (Panel B) subjects with diabetes mellitus.

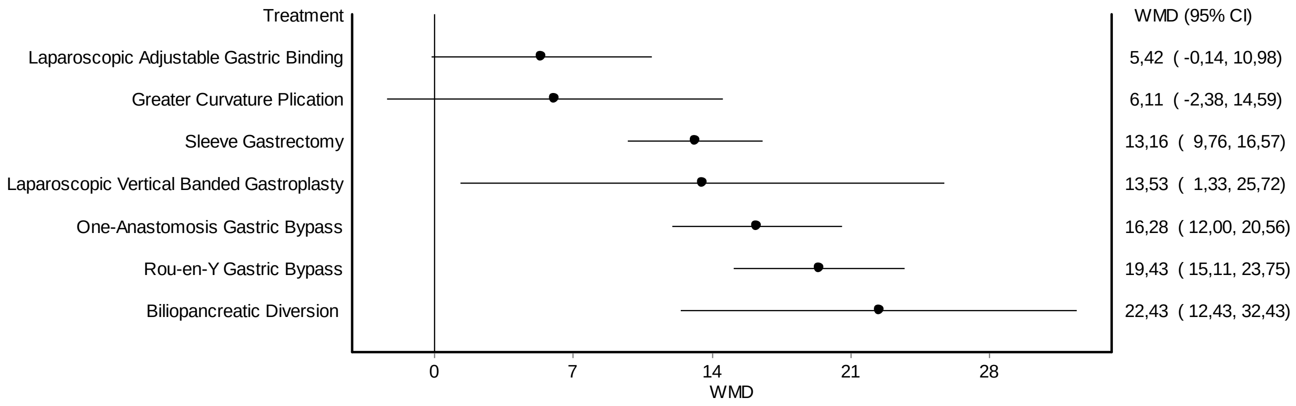

Secondary endpoints

Figure 23S – Network meta-analysis for different anti-obesity strategies. Network plot for risk of SAE at 26-52 weeks. For all analyses H values were <1.1 meaning no relevant inconsistency (H<3 indicates no relevant inconsistency of treatment effects).

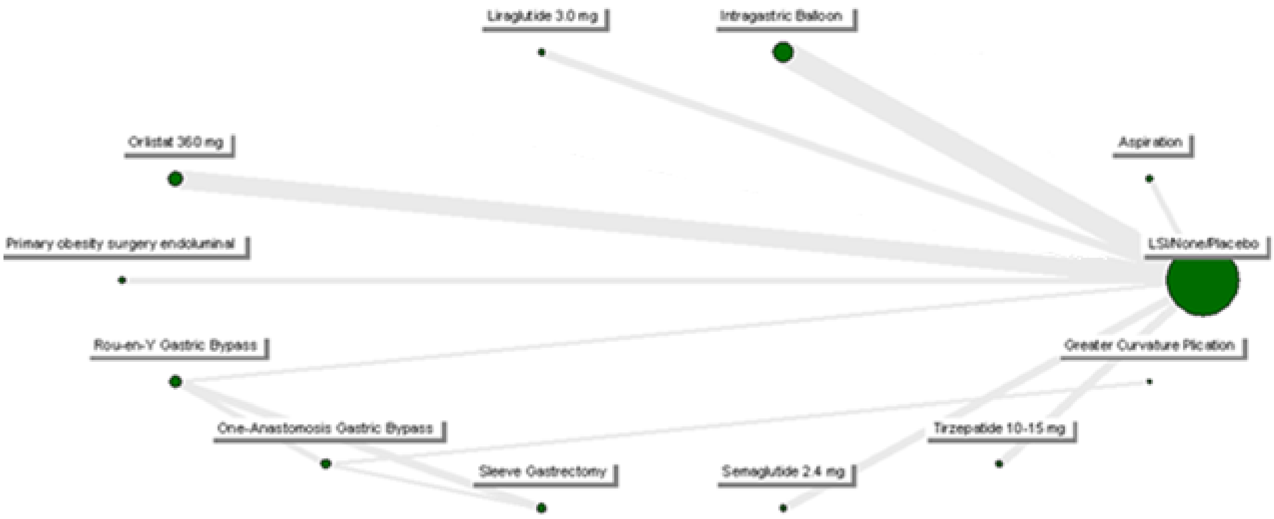

Figure 24S – Network meta-analysis for different anti-obesity strategies. Forest plot for risk of SAE at 26-52 weeks.

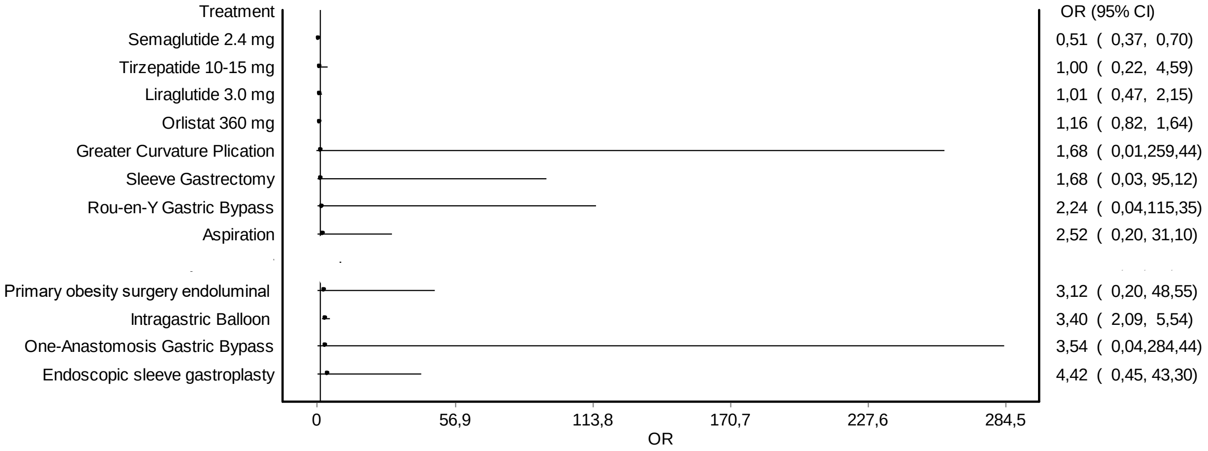

Figure 25S – Network meta-analysis for different anti-obesity strategies. Network plot for risk of SAE at 53-104 weeks. For all analyses H values were <1.1 meaning no relevant inconsistency (H<3 indicates no relevant inconsistency of treatment effects).

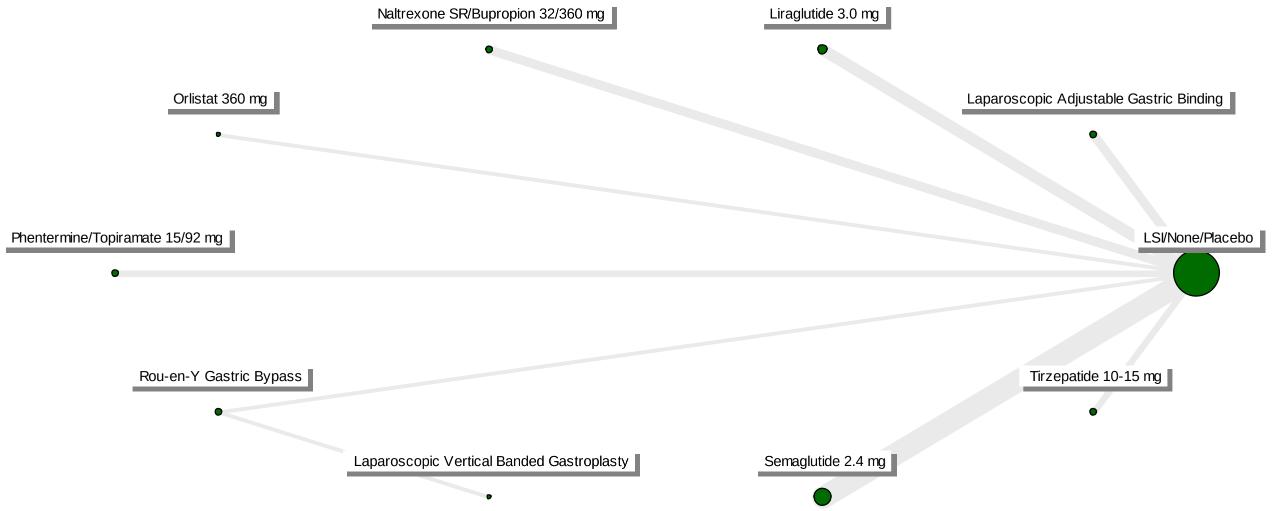

Figure 26S – Network meta-analysis for different anti-obesity strategies. Forest plot for risk of SAE at 53-104 weeks.

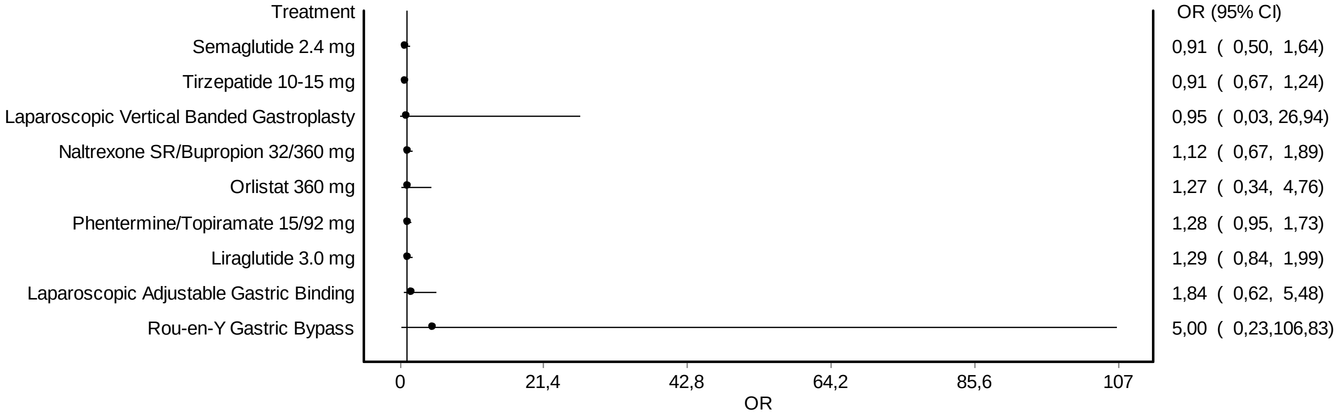

Figure 27S – Network meta-analysis for different anti-obesity strategies. Network plot for risk of SAE at 157-520 weeks. For all analyses H values were <1.1 meaning no relevant inconsistency (H<3 indicates no relevant inconsistency of treatment effects).

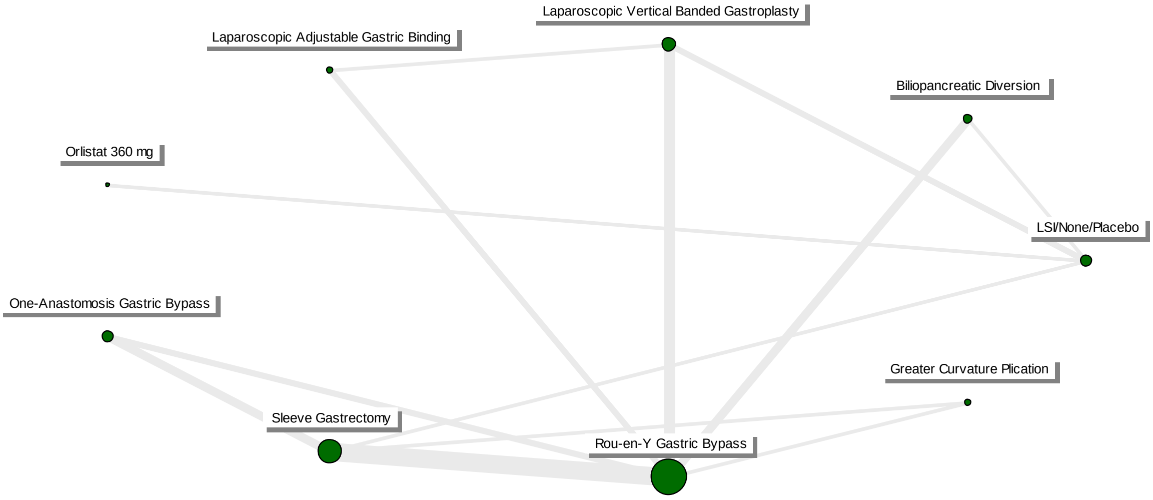

Figure 28S – Network meta-analysis for different anti-obesity strategies. Forest plot for risk of SAE at 157-520 weeks.

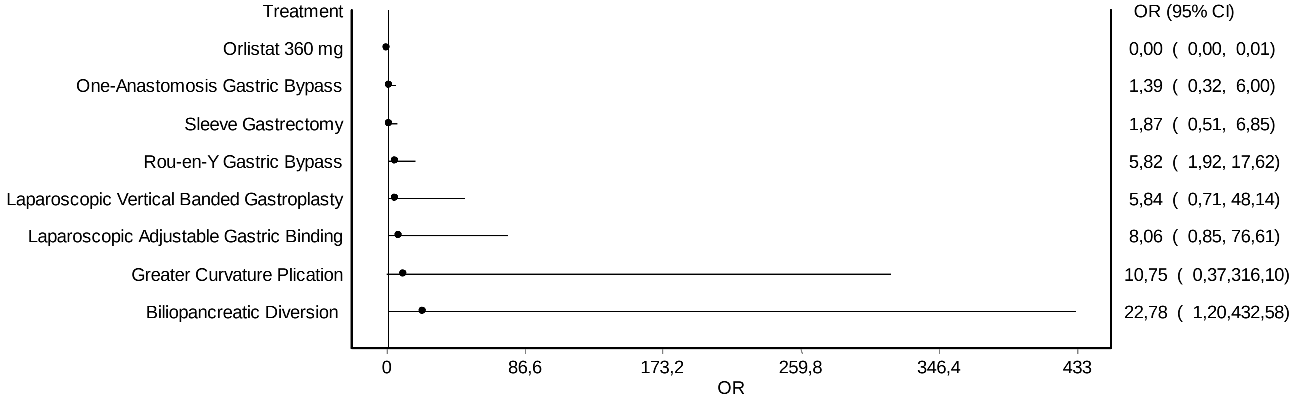

Figure 29S – Effects of different anti-obesity strategies on all-cause mortality at the endpoint. Panel A: Interventions versus Placebo/LSI/no therapy; Panel B: Active comparisons). OAGB: One-anastomosis gastric bypass; RYGB: Roux-en-Y Gastric By-Pass. Heterogeneity was assessed using I2 statistics and a value≥50% means a high grade of heterogeneity.

A

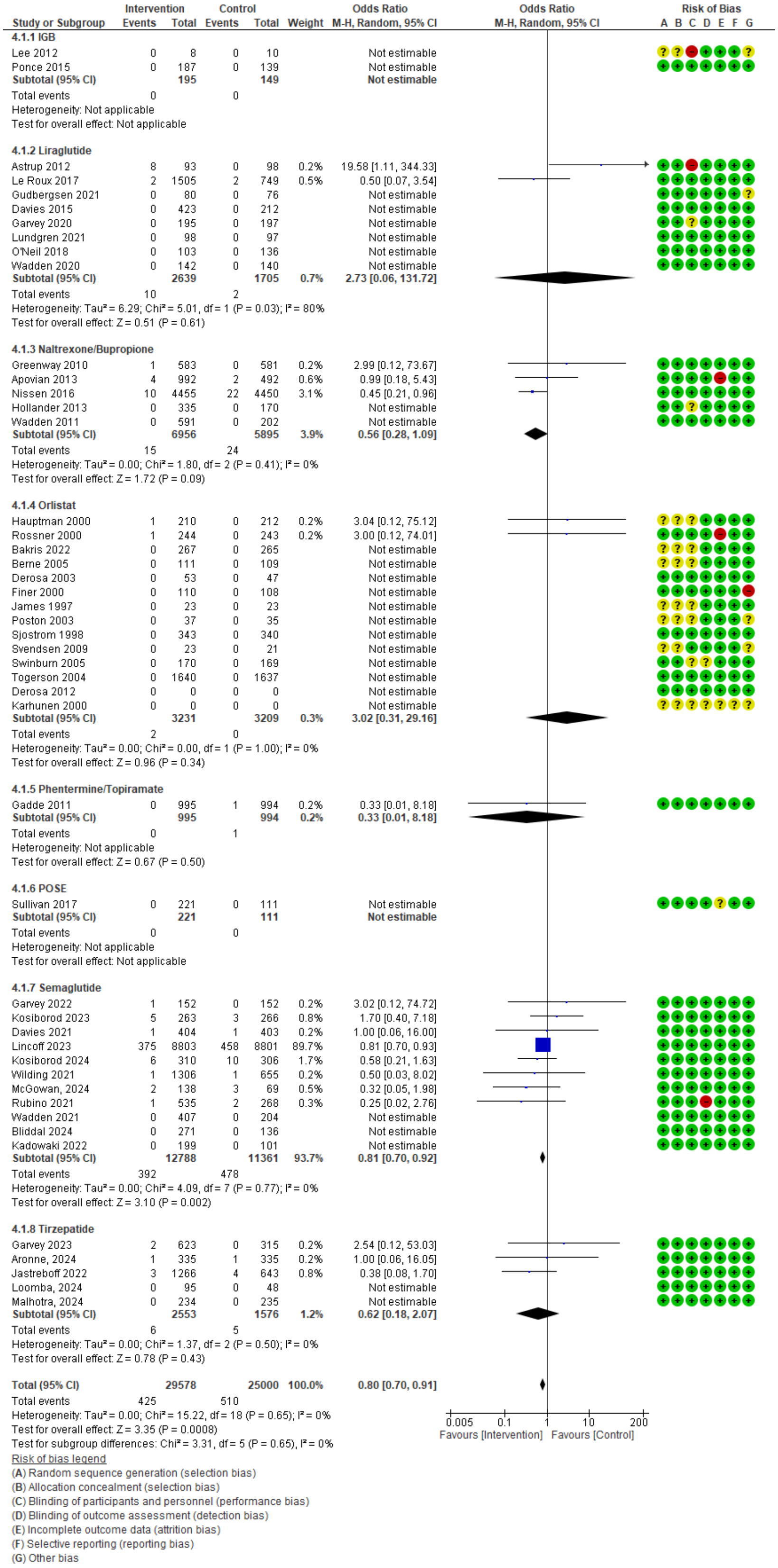

B

1) RYGB versus other metabolic bariatric surgery

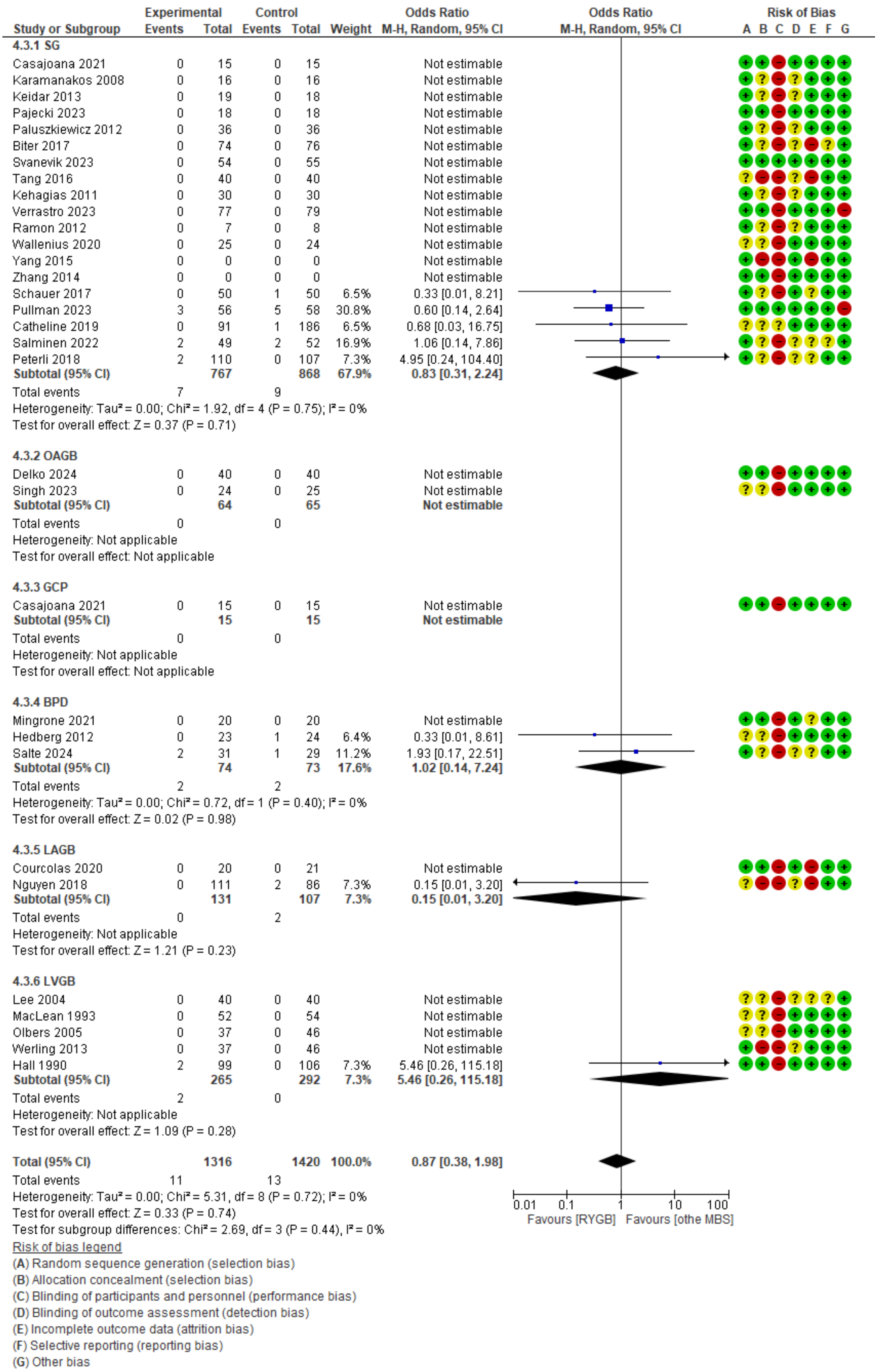

2) OAGB versus other metabolic bariatric surgery

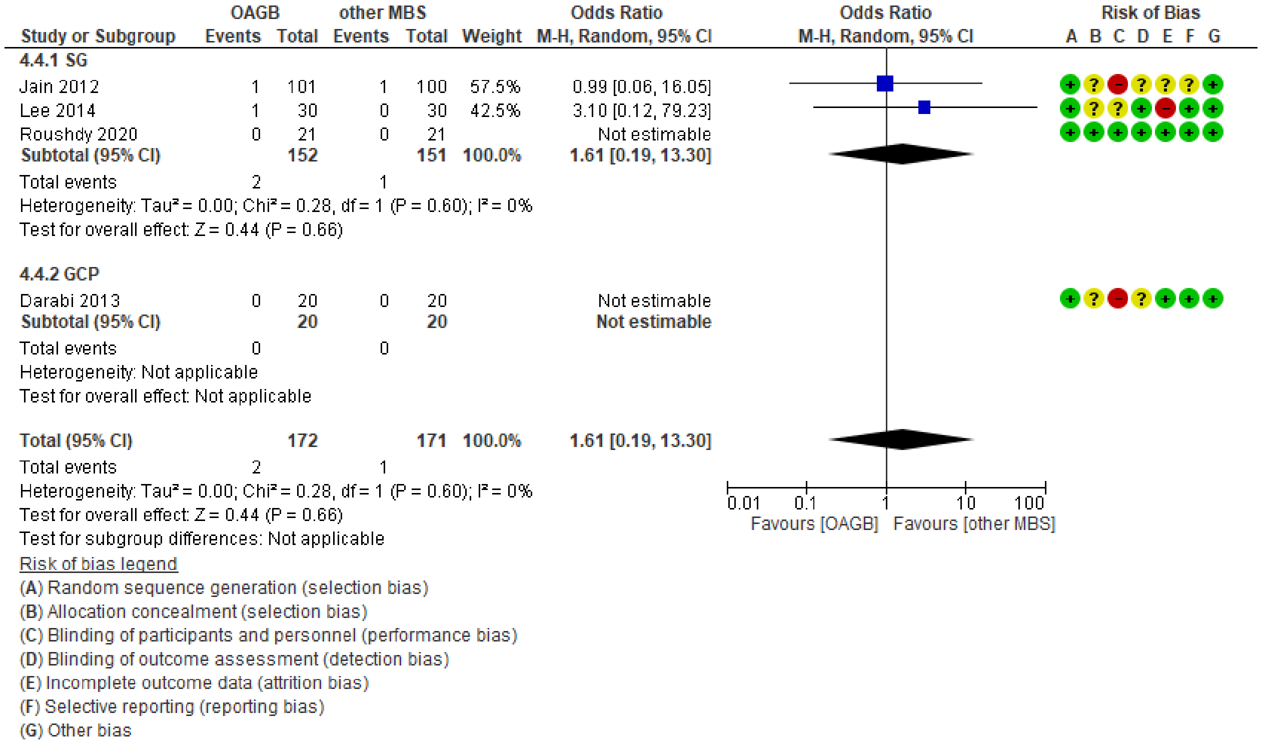

3) SG versus other metabolic bariatric surgery

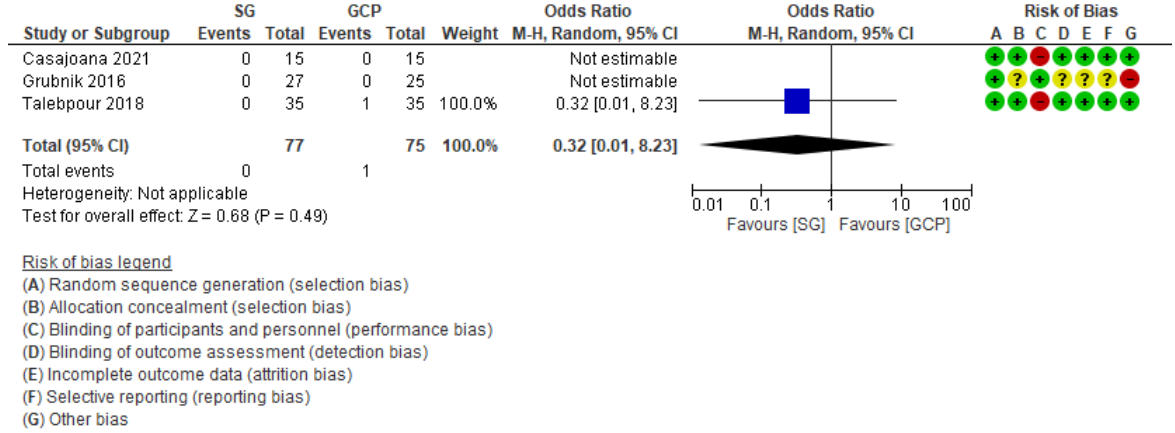

4) LAGB versus other metabolic bariatric surgery

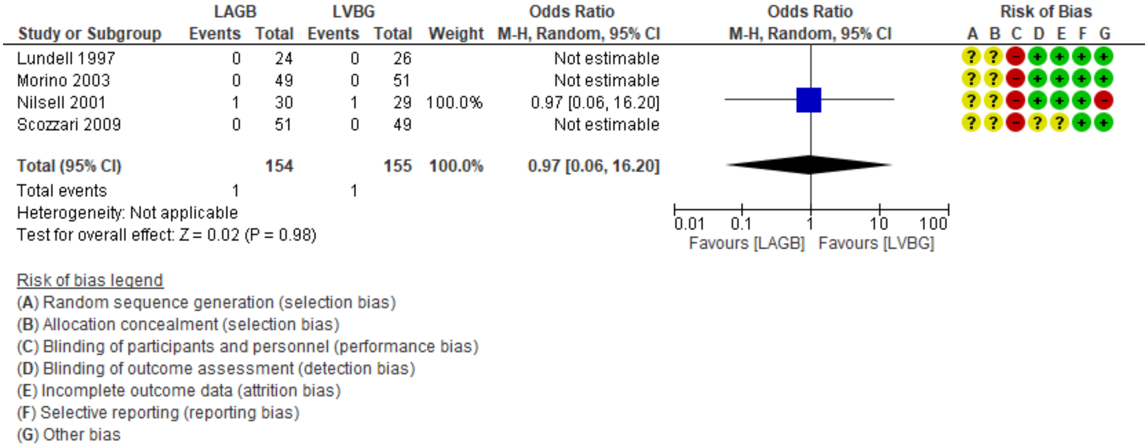

LAGB: Laparoscopic Adjustable Gastric Banding; **GCP**: Greater Curvature Plication Gastric; **LVGB**: Laparoscopic Vertical Banded Gastroplasty; **SG**: Sleeve Gastrectomy; **OAGB**: One-anastomosis gastric bypass; **RYGB**: Roux-en-Y Gastric By-Pass; **SADI**: Single Anastomosis Duodenal Switch; **BPD**: Bilio-Pancreatic Diversion.

Figure 30S – Effects of each anti-obesity strategy on all-cause mortality at endpoint in placebo- and active-controlled trials.

Intervention																			
Orlis	3.02	NA																	
NB	0.56	-	NA																
Liragl	2.73	ND	-	NA															
PT	0.33	-	-	-	NA														
Semag	**0.81**	-	-	-	-	NA													
Tirzep	0.62	-	-	-	-	-	NA												
IGB	ND	-	-	-	-	-	-	NA											
POSE	-	-	-	-	-	-	-	-	NA										
AT	-	-	-	-	-	-	-	-	-	NA									
ESG	-	-	-	-	-	-	-	-	-	-	NA								
GCP	-	-	-	-	-	-	-	-	-	-	-	NA							
LAGB	-	-	-	-	-	-	-	-	-	-	-	-	NA						
LVBG	-	-	-	-	-	-	-	-	-	-	-	-	1.03	NA					
SG	-	-	-	-	-	-	-	-	-	-	-	0.32	-	-	NA				
RYGB	-	-	-	-	-	-	-	-	-	-	-	ND	0.15	5.46	0.83	NA			
OAGB	-	-	-	-	-	-	-	-	-	-	-	ND	-	-	1.61	ND	NA		
SADI	-	-	-	-	-	-	-	-	-	-	-	-	-	-	-	-	-	NA	
BPD	-	-	-	-	-	-	-	-	-	-	-	3.9	-	-	-	1.02	-	ND	NA
**vs.**	*Pbo*	*Orlis*	*NB*	*Lirag.*	*PT*	*Semag*	*Tirzep*	*IGB*	*POSE*	*AT*	*ESG*	*GCP*	*LAGB*	*LVBG*	*SG*	*RYGB*	*OAGB*	*SADI*	*BPD*

-: no available data; ND: no deaths; Odds Ratio are in favor of (<1) or against (>1) the intervention in the first column. Significant results are reported in bold character. NA: Not applicable. **Naltr./Bupr.**: Naltrexone/Bupropion; **Phen./Topir.**: Phentermine/Topiramate; **POSE**: Primary Obesity Surgery Endoluminal; **IGB**: Intra-Gastric Balloon; **AT**: Aspiration Therapy; **ESG**: Endoscopic Sleeve Gastroplasty; **LAGB**: Laparoscopic Adjustable Gastric Banding; **GCP**: Greater Curvature Plication Gastric; **LVGB**: Laparoscopic Vertical Banded Gastroplasty; **SG**: Sleeve Gastrectomy; **OAGB**: One-anastomosis gastric bypass; **RYGB**: Roux-en-Y Gastric By-Pass; **SADI**: Single Anastomosis Duodenal Switch; **BPD**: Bilio-Pancreatic Diversion. For details please see Figure 27S.

Figure 31S – Effects of each anti-obesity strategy on quality of life at endpoint in placebo/LSI/no therapy- and active-controlled trials. Heterogeneity was assessed using I2 statistics and a value≥50% means a high grade of heterogeneity.

A The Impact of Weight on Quality of Life-Lite

LSI/no therapy/placebo-controlled RCT

Study or Subgroup	Experimental Mean	Experimental SD	Experimental Total	Placebo Mean	Placebo SD	Placebo Total	Weight	Mean Difference IV, Random, 95% CI
**6.1.1 Liraglutide**								
Astrup 2012	82	20	93	75.7	23	98	6.7%	6.30 [0.20, 12.40]
Davies 2015	84.3	20.4	423	83.3	18	212	10.2%	1.00 [-2.11, 4.11]
**Subtotal (95% CI)**			**516**			**310**	**16.9%**	**2.97 [-2.05, 7.99]**
Heterogeneity: Tau² = 7.94; Chi² = 2.30, df = 1 (P = 0.13); I² = 57%								
Test for overall effect: Z = 1.16 (P = 0.25)								
**6.1.2 Naltrexone/Bupriopone**								
Greenway 2010	83	16.5	583	80.4	17.2	581	11.4%	2.60 [0.66, 4.54]
Wadden 2011	85.6	14	591	83.7	14.8	202	11.0%	1.90 [-0.43, 4.23]
**Subtotal (95% CI)**			**1174**			**783**	**22.5%**	**2.31 [0.82, 3.80]**
Heterogeneity: Tau² = 0.00; Chi² = 0.20, df = 1 (P = 0.65); I² = 0%								
Test for overall effect: Z = 3.04 (P = 0.002)								
**6.1.3 POSE**								
Sullivan 2017	78.2	15	221	73.7	15	111	9.8%	4.50 [1.08, 7.92]
**Subtotal (95% CI)**			**221**			**111**	**9.8%**	**4.50 [1.08, 7.92]**
Heterogeneity: Not applicable								
Test for overall effect: Z = 2.58 (P = 0.010)								
**6.1.4 Semaglutide**								
Davies 2021	79	23.3	404	74.8	24.6	403	9.9%	4.20 [0.89, 7.51]
Kadowaki 2022	80	19.7	199	77.7	17.4	101	8.6%	2.30 [-2.06, 6.66]
Wilding 2021	78.3	21.2	1306	68.5	20.9	655	11.4%	9.80 [7.83, 11.77]
**Subtotal (95% CI)**			**1909**			**1159**	**30.0%**	**5.70 [0.84, 10.56]**
Heterogeneity: Tau² = 15.61; Chi² = 14.52, df = 2 (P = 0.0007); I² = 86%								
Test for overall effect: Z = 2.30 (P = 0.02)								
**6.1.5 Tirzepatide**								
Aronne, 2024	80.7	17.2	335	81.6	17.8	335	10.7%	-0.90 [-3.55, 1.75]
**Subtotal (95% CI)**			**335**			**335**	**10.7%**	**-0.90 [-3.55, 1.75]**
Heterogeneity: Not applicable								
Test for overall effect: Z = 0.67 (P = 0.51)								
**6.1.6 IGB**								
Courcoulas 2017	83	13	135	76.6	13	136	10.2%	6.40 [3.30, 9.50]
**Subtotal (95% CI)**			**135**			**136**	**10.2%**	**6.40 [3.30, 9.50]**
Heterogeneity: Not applicable								
Test for overall effect: Z = 4.05 (P < 0.0001)								
**Total (95% CI)**			**4290**			**2834**	**100.0%**	**3.76 [1.43, 6.09]**
Heterogeneity: Tau² = 11.35; Chi² = 58.64, df = 9 (P < 0.00001); I² = 85%								
Test for overall effect: Z = 3.17 (P = 0.002)								
Test for subgroup differences: Chi² = 15.37, df = 5 (P = 0.009), I² = 67.5%								

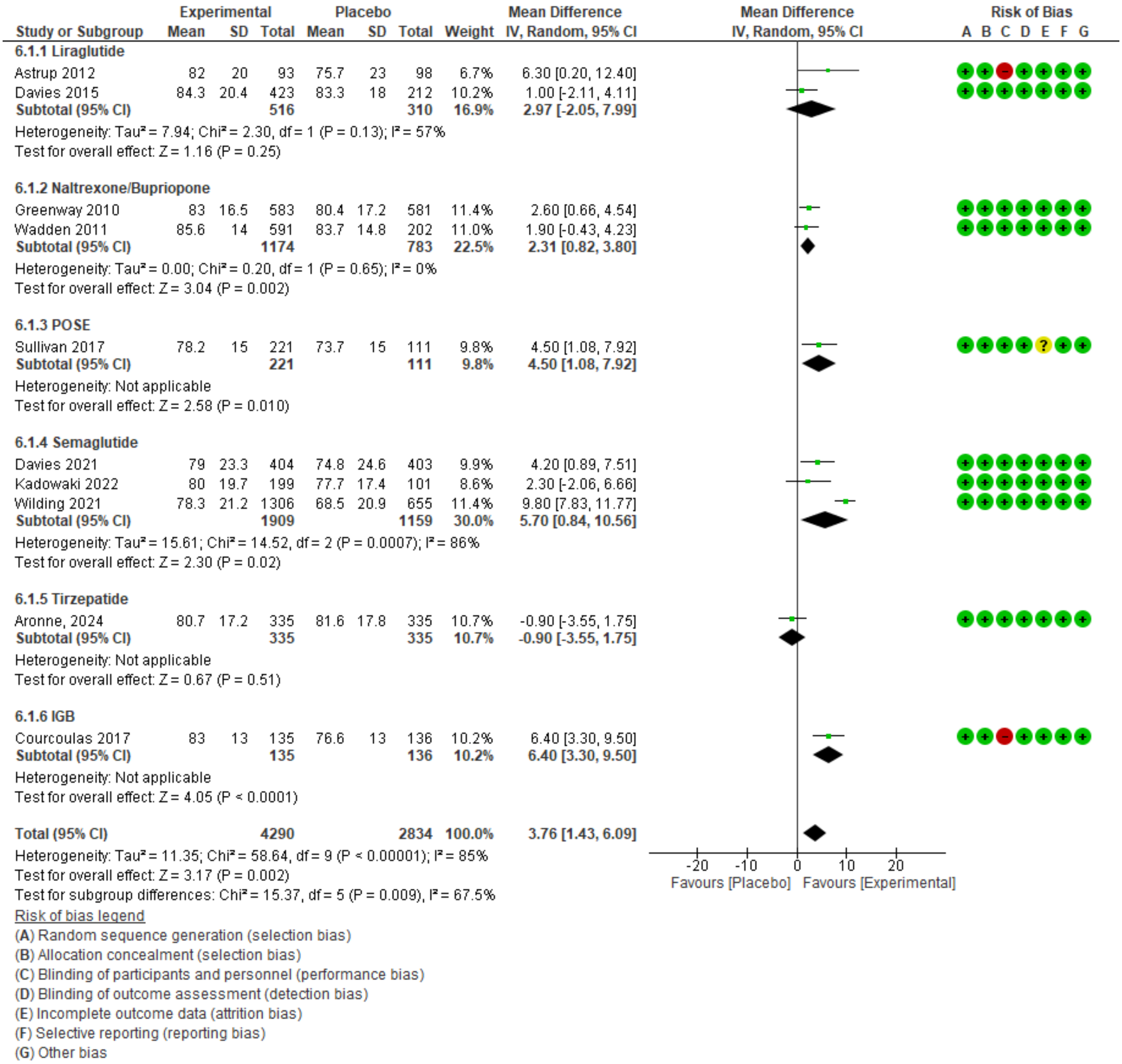

Risk of bias legend
(A) Random sequence generation (selection bias)
(B) Allocation concealment (selection bias)
(C) Blinding of participants and personnel (performance bias)
(D) Blinding of outcome assessment (detection bias)
(E) Incomplete outcome data (attrition bias)
(F) Selective reporting (reporting bias)
(G) Other bias

Active-controlled RCT

Study or Subgroup	Liraglutide Mean	Liraglutide SD	Liraglutide Total	Orlistat Mean	Orlistat SD	Orlistat Total	Weight	Mean Difference IV, Random, 95% CI
Astrup 2012	82	20	93	74	22	98	100.0%	8.00 [2.04, 13.96]
**Total (95% CI)**			**93**			**98**	**100.0%**	**8.00 [2.04, 13.96]**

Heterogeneity: Not applicable
Test for overall effect: Z = 2.63 (P = 0.008)

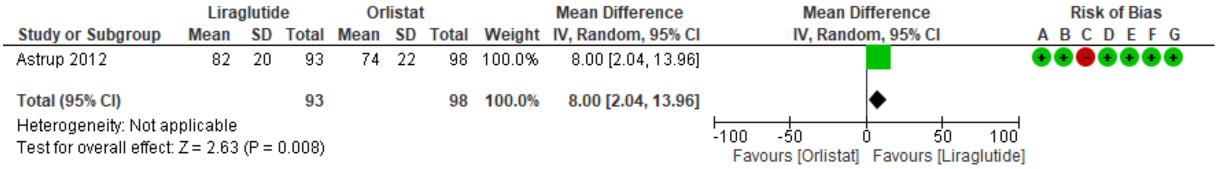

Risk of bias legend
(A) Random sequence generation (selection bias)
(B) Allocation concealment (selection bias)
(C) Blinding of participants and personnel (performance bias)
(D) Blinding of outcome assessment (detection bias)
(E) Incomplete outcome data (attrition bias)
(F) Selective reporting (reporting bias)
(G) Other bias

B Short-Form General Health

LSI/no therapy/placebo-controlled RCT

Study or Subgroup	Experimental Mean	Experimental SD	Experimental Total	LSI/Placebo/No therapy Mean	LSI/Placebo/No therapy SD	LSI/Placebo/No therapy Total	Weight	Mean Difference IV, Random, 95% CI
**6.2.1 BPD**								
Mingrone 2021	77.5	19	20	42.1	20.9	20	10.9%	35.40 [23.02, 47.78]
**Subtotal (95% CI)**			**20**			**20**	**10.9%**	**35.40 [23.02, 47.78]**
Heterogeneity: Not applicable								
Test for overall effect: Z = 5.60 (P < 0.00001)								
**6.2.2 IGB**								
Ponce 2013	81.4	13	21	86.1	14	9	11.5%	-4.70 [-15.40, 6.00]
Courcoulas 2017	75	15	135	65	13	136	13.5%	10.00 [6.66, 13.34]
**Subtotal (95% CI)**			**156**			**145**	**25.1%**	**3.57 [-10.73, 17.86]**
Heterogeneity: Tau^2 = 91.68; Chi^2 = 6.60, df = 1 (P = 0.01); I^2 = 85%								
Test for overall effect: Z = 0.49 (P = 0.62)								
**6.2.3 Liraglutide**								
Lundgren 2021	73.4	14	98	77	14	97	13.4%	-3.60 [-7.53, 0.33]
**Subtotal (95% CI)**			**98**			**97**	**13.4%**	**-3.60 [-7.53, 0.33]**
Heterogeneity: Not applicable								
Test for overall effect: Z = 1.80 (P = 0.07)								
**6.2.4 Orlistat**								
Swinburn 2005	72.4	19.6	170	70.2	18.4	169	13.4%	2.20 [-1.85, 6.25]
**Subtotal (95% CI)**			**170**			**169**	**13.4%**	**2.20 [-1.85, 6.25]**
Heterogeneity: Not applicable								
Test for overall effect: Z = 1.07 (P = 0.29)								
**6.2.5 RYGB**								
Schauer 2017	55.1	15.8	50	54.7	19.8	50	12.7%	0.40 [-6.62, 7.42]
Cohen 2020	78.1	12	51	54.8	10	49	13.4%	23.30 [18.98, 27.62]
Mingrone 2021	85.6	17.4	20	42.1	20.9	20	11.1%	43.50 [31.58, 55.42]
**Subtotal (95% CI)**			**121**			**119**	**37.2%**	**21.89 [1.89, 41.89]**
Heterogeneity: Tau^2 = 294.74; Chi^2 = 46.96, df = 2 (P < 0.00001); I^2 = 96%								
Test for overall effect: Z = 2.14 (P = 0.03)								
**6.2.6 SG**								
Schauer 2017	0	0	0	0	0	0		Not estimable
**Subtotal (95% CI)**			**0**			**0**		**Not estimable**
Heterogeneity: Not applicable								
Test for overall effect: Not applicable								
**Total (95% CI)**			**565**			**550**	**100.0%**	**12.48 [3.48, 21.49]**

Heterogeneity: Tau^2 = 153.19; Chi^2 = 153.63, df = 7 (P < 0.00001); I^2 = 95%
Test for overall effect: Z = 2.72 (P = 0.007)
Test for subgroup differences: Chi^2 = 39.46, df = 4 (P < 0.00001), I^2 = 89.9%

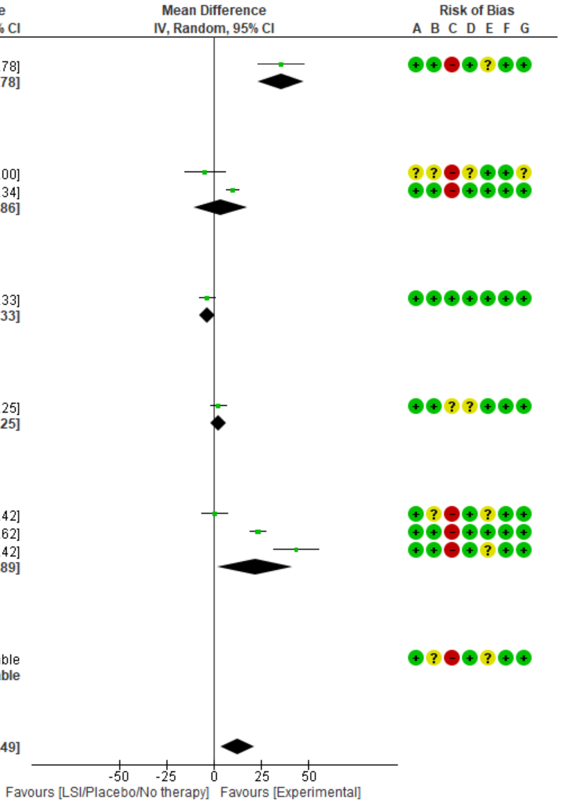

Risk of bias legend
(A) Random sequence generation (selection bias)
(B) Allocation concealment (selection bias)
(C) Blinding of participants and personnel (performance bias)
(D) Blinding of outcome assessment (detection bias)
(E) Incomplete outcome data (attrition bias)
(F) Selective reporting (reporting bias)
(G) Other bias

Active-controlled RCT

Study or Subgroup	RYGB Mean	RYGB SD	RYGB Total	other MBS Mean	other MBS SD	other MBS Total	Weight	Mean Difference IV, Random, 95% CI	Risk of Bias A	B	C	D	E	F	G
**6.3.1 BPD**															
Salte 2024	58	10	31	60	10	29	100.0%	-2.00 [-7.06, 3.06]	+	?	-	?	?	+	+
**Subtotal (95% CI)**			**31**			**29**	**100.0%**	**-2.00 [-7.06, 3.06]**							
Heterogeneity: Not applicable															
Test for overall effect: Z = 0.77 (P = 0.44)															
**6.3.2 SG**															
Biter 2017	90.2	20.2	74	93.5	14.6	76	29.2%	-3.30 [-8.95, 2.35]	+	?	-	?	-	?	+
Catheline 2019	96	17.3	91	98	15.8	186	52.4%	-2.00 [-6.22, 2.22]	?	?	?	+	+	+	+
Schauer 2017	55.1	15.8	50	52.3	20.3	50	18.4%	2.80 [-4.33, 9.93]	+	?	-	+	?	+	+
**Subtotal (95% CI)**			**215**			**312**	**100.0%**	**-1.50 [-4.55, 1.56]**							
Heterogeneity: Tau² = 0.00; Chi² = 1.84, df = 2 (P = 0.40); I² = 0%															
Test for overall effect: Z = 0.96 (P = 0.34)															

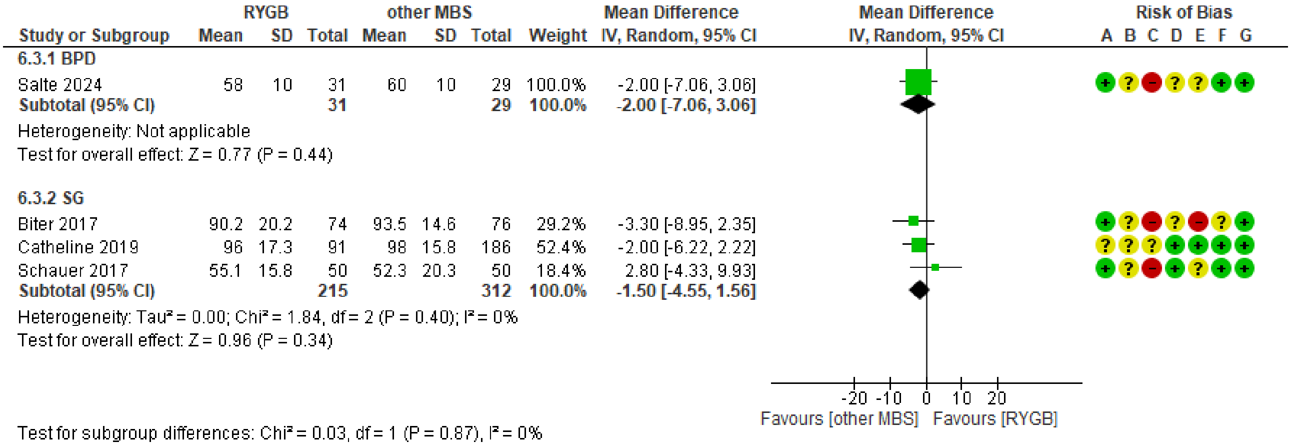

Test for subgroup differences: Chi² = 0.03, df = 1 (P = 0.87), I² = 0%

Risk of bias legend
(A) Random sequence generation (selection bias)
(B) Allocation concealment (selection bias)
(C) Blinding of participants and personnel (performance bias)
(D) Blinding of outcome assessment (detection bias)
(E) Incomplete outcome data (attrition bias)
(F) Selective reporting (reporting bias)
(G) Other bias

C Short-Form-36 Physical Functioning

LSI/no therapy/placebo-controlled RCT

Study or Subgroup	Experimental Mean	Experimental SD	Experimental Total	Control Mean	Control SD	Control Total	Weight	Mean Difference IV, Random, 95% CI	Risk of Bias A B C D E F G
**6.4.1 IGB**									
Ponce 2013	92	13.3	21	87.1	11.9	9	2.4%	4.90 [-4.73, 14.53]	? ? − ? + + ?
**Subtotal (95% CI)**			**21**			**9**	**2.4%**	**4.90 [-4.73, 14.53]**	
Heterogeneity: Not applicable									
Test for overall effect: Z = 1.00 (P = 0.32)									
**6.4.2 Liraglutide**									
Lundgren 2021	78.2	14	98	81	14	97	8.2%	-2.80 [-6.73, 1.13]	+ + + + + + +
**Subtotal (95% CI)**			**98**			**97**	**8.2%**	**-2.80 [-6.73, 1.13]**	
Heterogeneity: Not applicable									
Test for overall effect: Z = 1.40 (P = 0.16)									
**6.4.3 Orlistat**									
Swinburn 2005	78.7	19.6	170	77	19.5	169	7.7%	1.70 [-2.46, 5.86]	+ + ? ? + + +
**Subtotal (95% CI)**			**170**			**169**	**7.7%**	**1.70 [-2.46, 5.86]**	
Heterogeneity: Not applicable									
Test for overall effect: Z = 0.80 (P = 0.42)									
**6.4.4 Semaglutide**									
Davies 2021	52.1	7.9	404	50.5	9	403	14.4%	1.60 [0.43, 2.77]	+ + + + + + +
Rubino 2021	54.8	5.7	535	53.1	5	268	15.0%	1.70 [0.93, 2.47]	+ + + − + + +
Wilding 2021	53.2	6.9	1306	51.6	7.9	655	15.1%	1.60 [0.89, 2.31]	+ + + + + + +
**Subtotal (95% CI)**			**2245**			**1326**	**44.4%**	**1.64 [1.16, 2.12]**	
Heterogeneity: Tau² = 0.00; Chi² = 0.04, df = 2 (P = 0.98); I² = 0%									
Test for overall effect: Z = 6.73 (P < 0.00001)									
**6.4.5 Tirzepatide**									
Aronne, 2024	53.4	5.8	335	53.2	6.5	335	14.8%	0.20 [-0.73, 1.13]	+ + + + + + +
Jastreboff 2022	53.6	7.6	1266	51.6	7.7	643	15.0%	2.00 [1.27, 2.73]	+ + + + + + +
**Subtotal (95% CI)**			**1601**			**978**	**29.8%**	**1.12 [-0.64, 2.89]**	
Heterogeneity: Tau² = 1.44; Chi² = 8.89, df = 1 (P = 0.003); I² = 89%									
Test for overall effect: Z = 1.25 (P = 0.21)									
**6.4.6 RYGB**									
Cohen 2020	78.1	12	51	54.8	10	49	7.4%	23.30 [18.98, 27.62]	+ + − + + + +
**Subtotal (95% CI)**			**51**			**49**	**7.4%**	**23.30 [18.98, 27.62]**	
Heterogeneity: Not applicable									
Test for overall effect: Z = 10.56 (P < 0.00001)									
**Total (95% CI)**			**4186**			**2628**	**100.0%**	**2.81 [1.18, 4.45]**	

Heterogeneity: Tau² = 4.49; Chi² = 111.92, df = 8 (P < 0.00001); I² = 93%
Test for overall effect: Z = 3.37 (P = 0.0008)
Test for subgroup differences: Chi² = 101.69, df = 5 (P < 0.00001), I² = 95.1%

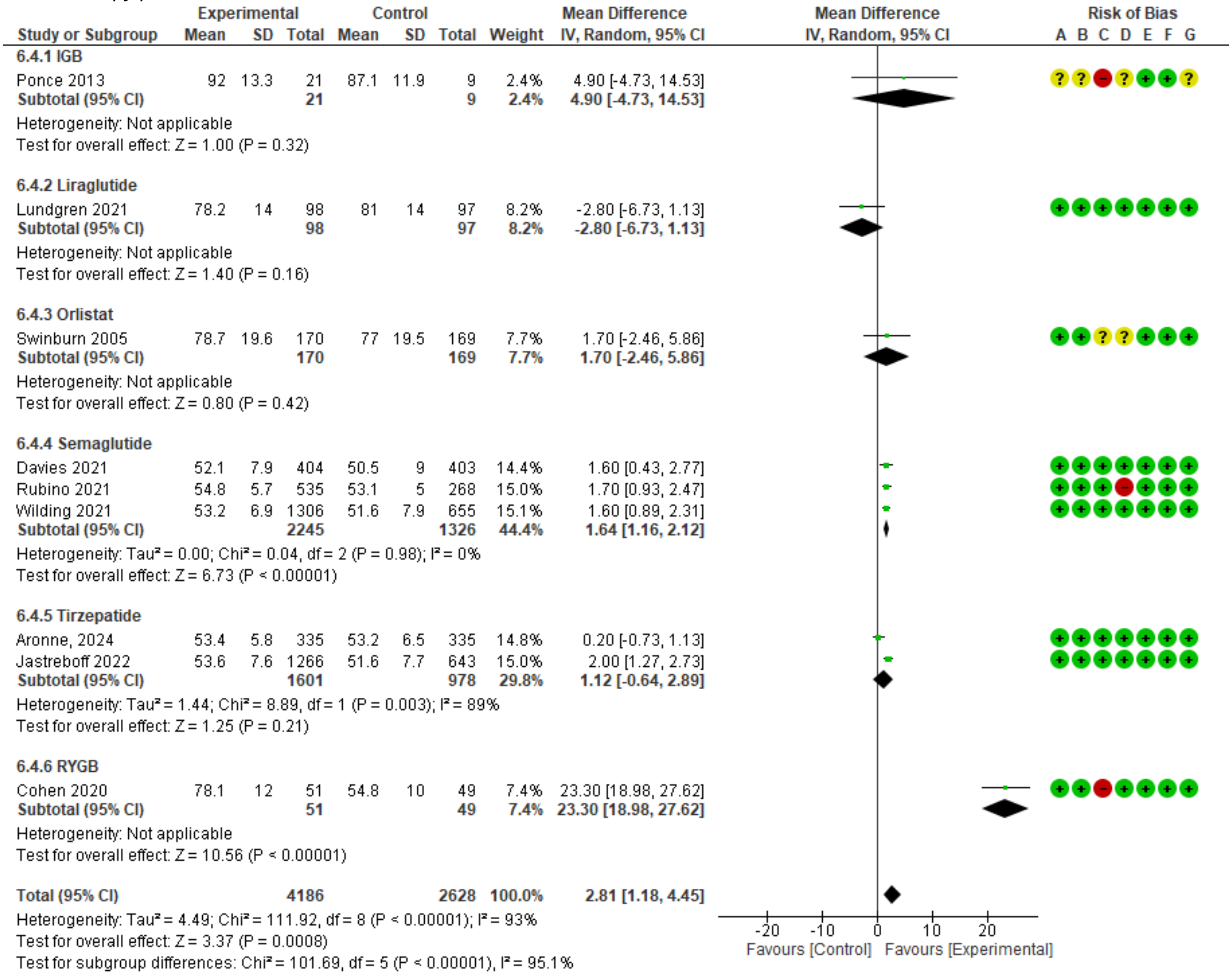

Risk of bias legend
(A) Random sequence generation (selection bias)
(B) Allocation concealment (selection bias)
(C) Blinding of participants and personnel (performance bias)
(D) Blinding of outcome assessment (detection bias)
(E) Incomplete outcome data (attrition bias)
(F) Selective reporting (reporting bias)
(G) Other bias

D Short-Form-36 Physical Component

LSI/no therapy/placebo-controlled RCT

Study or Subgroup	Experimental Mean	Experimental SD	Experimental Total	Control Mean	Control SD	Control Total	Weight	Mean Difference IV, Random, 95% CI
**6.6.1 LAGB**								
O'Brien 2013	48	10.5	40	49.3	5.6	10	6.0%	-1.30 [-6.06, 3.46]
**Subtotal (95% CI)**			**40**			**10**	**6.0%**	**-1.30 [-6.06, 3.46]**
Heterogeneity: Not applicable								
Test for overall effect: Z = 0.54 (P = 0.59)								
**6.6.2 IGB**								
Ponce 2013	53.2	15	21	52.1	16	9	0.9%	1.10 [-11.16, 13.36]
**Subtotal (95% CI)**			**21**			**9**	**0.9%**	**1.10 [-11.16, 13.36]**
Heterogeneity: Not applicable								
Test for overall effect: Z = 0.18 (P = 0.86)								
**6.6.3 Semaglutide**								
Wadden 2021	54.2	6.9	407	53.7	7.3	204	93.1%	0.50 [-0.71, 1.71]
**Subtotal (95% CI)**			**407**			**204**	**93.1%**	**0.50 [-0.71, 1.71]**
Heterogeneity: Not applicable								
Test for overall effect: Z = 0.81 (P = 0.42)								
**Total (95% CI)**			**468**			**223**	**100.0%**	**0.40 [-0.77, 1.56]**

Heterogeneity: Tau² = 0.00; Chi² = 0.53, df = 2 (P = 0.77); I² = 0%
Test for overall effect: Z = 0.67 (P = 0.50)
Test for subgroup differences: Chi² = 0.53, df = 2 (P = 0.77), I² = 0%

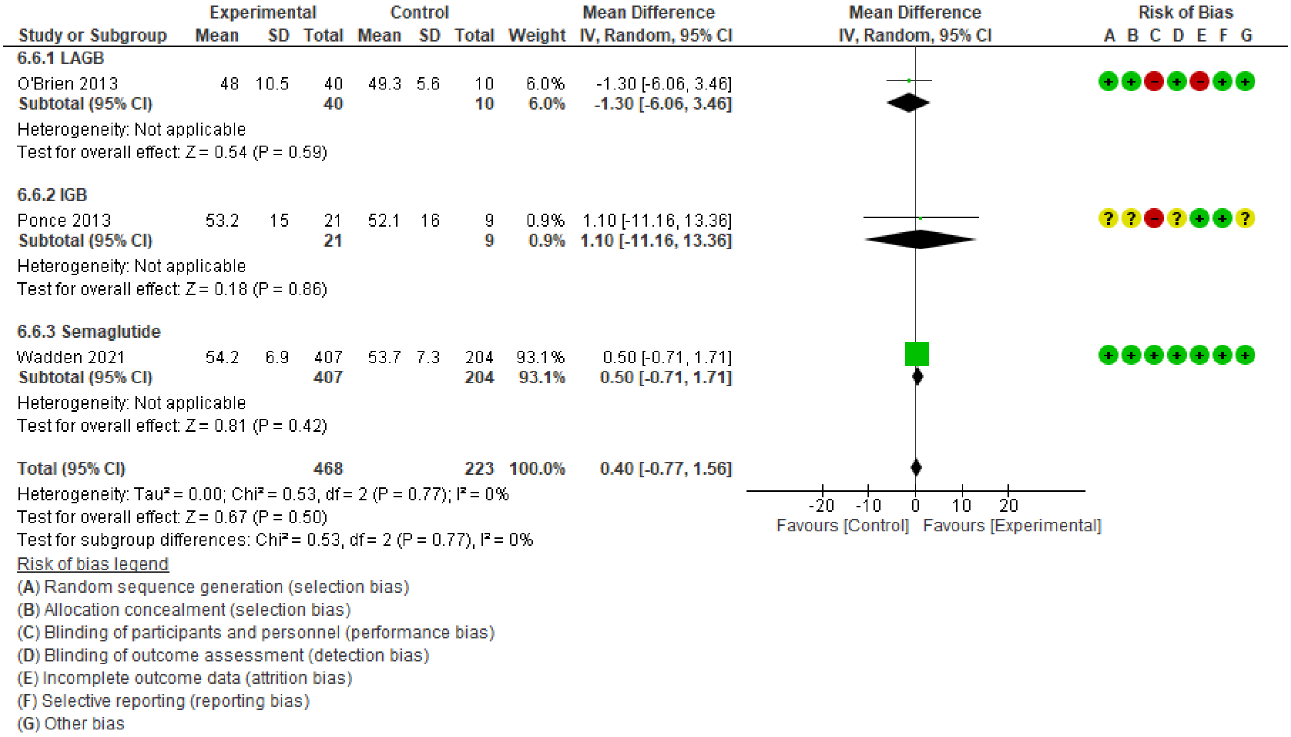

Risk of bias legend
(A) Random sequence generation (selection bias)
(B) Allocation concealment (selection bias)
(C) Blinding of participants and personnel (performance bias)
(D) Blinding of outcome assessment (detection bias)
(E) Incomplete outcome data (attrition bias)
(F) Selective reporting (reporting bias)
(G) Other bias

Active-controlled RCT

Study or Subgroup	RYGB Mean	RYGB SD	RYGB Total	SG Mean	SG SD	SG Total	Weight	Mean Difference IV, Random, 95% CI
Biter 2017	79.3	13.1	74	80	13.4	76	26.6%	-0.70 [-4.94, 3.54]
Catheline 2019	49.6	10.3	91	50.1	9.9	186	73.4%	-0.50 [-3.05, 2.05]
**Total (95% CI)**			**165**			**262**	**100.0%**	**-0.55 [-2.74, 1.63]**

Heterogeneity: Tau² = 0.00; Chi² = 0.01, df = 1 (P = 0.94); I² = 0%
Test for overall effect: Z = 0.50 (P = 0.62)

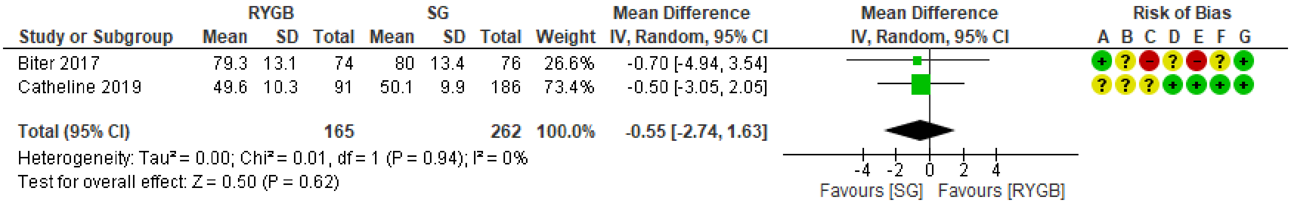

Risk of bias legend
(A) Random sequence generation (selection bias)
(B) Allocation concealment (selection bias)
(C) Blinding of participants and personnel (performance bias)
(D) Blinding of outcome assessment (detection bias)
(E) Incomplete outcome data (attrition bias)
(F) Selective reporting (reporting bias)
(G) Other bias

TABLES

Table 1S – PRISMA checklist.

Section/topic	#	Checklist item	Reported on page #
**TITLE**			
Title	1	Obesity and overweight in adults: efficacy and safety of 2024-EMA- and FDA-approved surgical, endoscopic, and pharmacological treatments. A comprehensive systematic review and network meta-analysis of randomized controlled trials.	-
**ABSTRACT**			
Structured summary	2	Background: To compare different anti-obesity strategies (Obesity Management Medications – OMM, Endoscopic Bariatric Procedures – EBP, and Metabolic Bariatric Surgery – MBS) with Lifestyle Intervention/Placebo/No therapy (LSI/Pbo/NT) for the treatment of overweight/obesity. Methods: This systematic review and Network Meta-Analysis (NMA) included randomized clinical trials (RCTs) comparing OMM, EBP, and MBS versus either LSI/Pbo/NT or active comparators in subjects affected by obesity/overweight. A Medline and Embase search was performed up to 1st December 2024 for RCTs on approved weight-lowering interventions in adults with overweight or obesity. The primary endpoint was Total Body Weight Loss (%; TBWL%) analyzed at different time points: 26-52, 53-104, 105-156, and >156 weeks. Secondary endpoints included all-cause mortality, quality of life, and serious adverse events (SAE). Weighted Mean Difference and 95% Confidence Intervals (WMD, 95% CI) for continuous variables, and Mantel-Haenzel Odds Ratio (MH-OR, 95% CI) for categorical variables were calculated, using random effect models. The study was registered with PROSPERO, CRD42024625338.<br>Findings: Out of 139 trials, 54, 21, and 64 trials were performed on MBS, EP, and OMMs, enrolling 61,961, 2,934, and 5,991, respectively. At 26-52 weeks (N=104 comparisons, with no evidence of inconsistency; H=1.07), all treatments showed a significant effect versus reference (LSI/None/Placebo), except Primary Obesity Surgery, Endoluminal (POSE) and LAGB (Laparoscopic Adjustable Gastric Banded); the estimated TBWL was greater than 10% for most surgical procedures and tirzepatide. Only a limited number of comparisons was available at 53-104 weeks (N=42), 105-156 weeks (N=28), and 156-520 weeks (N=26). No long-term data was available for EBP and for most OMM (except orlistat). For the majority of the assessed treatments, the estimated longer-term efficacy was similar to that at 26-52 weeks, with the notable exception of LAGB and GCP, the effects of which seem to fade after 104 weeks. EBP and MBS were generally associated with a greater risk of SAE than OMM. In the long term, Bilio-Pancreatic Diversion) appeared to produce a greater incidence of SAE than other types of MBS.	1

		Interpretation: the results obtained are in our opinion of interest for clinicians involved in the management of overweight and obesity. For the first time, different approaches have been compared in different categories of patients, giving clearer overall picture of their effectiveness. EBP, with exception of ESG, appear to be manifestly inferior to the most commonly used OMM (i.e., semaglutide and tirzepatide), which seem to be competitive even in candidates for MBS. Some surgical interventions seem to be outdated (i.e. LAGB, and GCP) or falsely considered more effective (BPD) than others and therefore they should be abandoned.	
**INTRODUCTION**			
Rationale	3	The development of pharmacological, endoscopic, and surgical research has led to the discovery of novel treatments in recent years, providing clinicians with a relatively wide range of therapeutic options. However, the availability of randomized control trials (RCTs) directly comparing different OMMs, other than LSIs, is still limited. In particular, there are very few studies comparing OMMs with MBS. A Network Meta-Analysis (NMA), providing indirect comparisons of efficacy and safety, can therefore be of help in guiding physicians' choices.	3
Objectives	4	The present network meta-analysis is aimed the assessment of the effect of OMM, EBP, and MBS in subjects affected by overweight/obesity with respect of body weight loss, mortality, SAE, and quality of life.	3
**METHODS**			
Protocol and registration	5	The study has been uploaded on PROSPERO website (#CRD42024625338).	4
Eligibility criteria	6	All studies were included if they satisfied the following criteria: (i) randomized trials. (ii) duration ≥52 weeks for OMM and MBS and ≥26 for EBP. (iii) on obesity/overweight and (iv) comparing antiobesity strategies versus LSI/Pbo/NT, or comparing two different strategies.	4
Information sources	7	A MEDLINE and embase database search were performed to identify all clinical trials (English only) up to December 1st, 2024.	4
Search	8	See Table 2S.	4 and SM
Study selection	9	The identification of relevant abstracts. the selection of studies. and extraction were performed independently by two of the authors (B.R. and A.B.) and conflicts resolved by a fourth investigator (M.M.).	5
Data collection process	10	The principal characteristics of the included trials included were reported in Table 5S.	6 and SM
Data items	11	Information on the baseline characteristics of the samples enrolled (age, gender, proportion of people with diabetes, baseline Body Mass Index – BMI), Total Body Weight Loss (TBWL%), Quality of Life (QoL), Serious Adverse Events (SAE), and all-cause mortality were extracted from	6 and SM

		the principal publication, when available (Table 3S of Supplementary Materials reports a detailed list)	
Risk of bias in individual studies	12	The quality of trials was assessed using the parameters proposed by the Cochrane Collaboration.	6
Summary measures	13	Mantel-Haenszel odds ratio (MH-OR) and Weighted Mean Difference (WMD) with 95% Confidence Interval (95%, CI) were calculated for all outcomes defined above on an intention-to-treat basis.	5-6
Synthesis of results	14	Heterogeneity was assessed by using I^2 statistics. Random-effects model was applied in the primary analysis. We performed a Network Meta-Analysis (NMA) for all the outcomes listed above, in order to verify differences across individual anti-obesity strategies concerning their effects on primary and secondary endpoints.	6
**Section/topic**	**#**	**Checklist item**	**Reported on page #**
Risk of bias across studies	15	The risk of bias was assessed using the Cochrane recommended tool to determine the risk of bias in RCTs	5
Additional analyses	16	Several prespecified subgroup analyses were performed for the following baseline variables: different types of anti-obesity strategies (i.e., surgical and endoscopic procedures, and anti-obesity drugs), BMI categories (mean BMI at the enrolment <30, 30-34.9, 35-39.9, and >40 kg/m^2), and type 2 diabetes mellitus (T2DM; (yes: RCT enrolling at least 75% of subjects with diabetes; no: RCT enrolling no more than 25% of subjects with T2DM). Traditional meta-analyses were performed for all the endpoints for placebo- and active-controlled trials.	5-6
**RESULTS**			
Study selection	17	The search of Medline and Embase database allowed the identification of 139 trials (i.e., 150 comparisons).	7 and SM
Study characteristics	18	The principal characteristics of included trials were reported in Supplementary Materials (Table 5S).	7 and SM
Risk of bias within studies	19	The quality of studies was heterogeneous.	7 and SM
Results of individual studies	20	Forest plots for any primary and secondary endpoint are reported in the Results section and Supplementary Materials.	8-10; SM
Synthesis of results	21	At 26-52 weeks (N=104 comparisons, with no evidence of inconsistency; H=1.07), all treatments showed a significant effect versus reference (LSI/None/Placebo), except Primary Obesity Surgery, Endoluminal (POSE) and LAGB (Laparoscopic Adjustable Gastric Banded); the estimated TBWL was greater than 10% for most surgical procedures and tirzepatide. Only a	8-10; SM

		limited number of comparisons was available at 53-104 weeks (N=42), 105-156 weeks (N=28), and 156-520 weeks (N=26). No long-term data was available for EBP and for most OMM (except orlistat). For the majority of the assessed treatments, the estimated longer-term efficacy was similar to that at 26-52 weeks, with the notable exception of LAGB and GCP, the effects of which seem to fade after 104 weeks. EBP and MBS were generally associated with a greater risk of SAE than OMM. In the long term, Bilio-Pancreatic Diversion) appeared to produce a greater incidence of SAE than other types of MBS.	
Risk of bias across studies	22	The quality of studies was heterogeneous (Figure 2S of Supporting Information). All trials on surgical and endoscopic procedures, except seven (11%) were open-label. In many trials, the attrition rate and/or the description of allocation and blinding of assessors were inadequate (Figure 2S and 3S of Supporting Information). Trials on OMM were more frequently double-blind (66%), with fewer trials with inadequate attrition and/or description of allocation or of blinding of assessors (29.3%).	7 and SM
Additional analysis	23	Subgroups analyses of trials are available at pg. 8-9.	8-9 and SM
**DISCUSSION**			
Summary of evidence	24	The GRADE profile classified the strength as moderate for the primary endpoint.	9 and SM
Limitations	25	Limitations were reported in extenso in the Discussion section.	12-13
Conclusions	26	In conclusion, despite the above-cited limitations, the results obtained are in our opinion of interest for clinicians involved in the management of overweight and obesity. For the first time, different approaches have been compared in different categories of patients, giving clearer overall picture of their effectiveness. EBP, with exception of ESG, appear to be manifestly inferior to the most commonly used OMM (i.e., semaglutide and tirzepatide), which seem to be competitive even in candidates for MBS. Some surgical interventions seem to be outdated (i.e. LAGB, and GCP) or falsely considered more effective (BPD) than others and therefore they should be abandoned.	12, 13
**FUNDING**			
Funding	27	The funders (scientific societies: IFSO, EASO, SICOB, and SIO) had no role in data collection, analysis, and interpretation, or writing of the manuscript and the decision to submit.	14

Table 2S – Detailed information on search strategy

**Limits:** Human studies; any date up to December 1st. 2024<br>**N= 1,540**
**Search string:** (obesity or overweight) AND (orlistat OR phentermine OR topiramate OR naltrexone OR bupropion OR liraglutide OR semaglutide OR tirzepatide OR Sleeve Gastrectomy OR Roux en Y Gastric Bypass OR One Anastomosis Gastric Bypass OR Laparoscopic Adjustable Gastric Banding OR Biliopancreatic Diversion OR Single Anastomosis Duodenal-Ileal bypass OR Intragastric Balloons OR Primary Obesity Surgery Endoluminal OR Endoscopic Sleeve Gastroplasty)<br><br>**Pubmed**<br>(("obeses"[All Fields] OR "obesity"[MeSH Terms] OR "obesity"[All Fields] OR "obese"[All Fields] OR "obesities"[All Fields] OR "obesity s"[All Fields] OR ("overweight"[MeSH Terms] OR "overweight"[All Fields] OR "overweighted"[All Fields] OR "overweightness"[All Fields] OR "overweights"[All Fields])) AND ("orlistat"[MeSH Terms] OR "orlistat"[All Fields] OR "orlistat s"[All Fields] OR ("phentermine"[MeSH Terms] OR "phentermine"[All Fields]) OR ("topiramate"[MeSH Terms] OR "topiramate"[All Fields] OR "topiramate s"[All Fields]) OR ("naltrexone"[MeSH Terms] OR "naltrexone"[All Fields] OR "naltrexon"[All Fields] OR "naltrexone s"[All Fields]) OR ("bupropion"[MeSH Terms] OR "bupropion"[All Fields] OR "amfebutamone"[All Fields] OR "bupropion s"[All Fields] OR "bupropione"[All Fields]) OR ("liraglutid"[All Fields] OR "liraglutide"[MeSH Terms] OR "liraglutide"[All Fields] OR "liraglutide s"[All Fields]) OR ("semaglutide"[Supplementary Concept] OR "semaglutide"[All Fields]) OR ("tirzepatide"[Supplementary Concept] OR "tirzepatide"[All Fields]) OR (("sleeve"[All Fields] OR "sleeved"[All Fields] OR "sleeves"[All Fields] OR "sleeving"[All Fields]) AND ("gastrectomy"[MeSH Terms] OR "gastrectomy"[All Fields] OR "gastrectomies"[All Fields])) OR ("gastric bypass"[MeSH Terms] OR ("gastric"[All Fields] AND "bypass"[All Fields]) OR "gastric bypass"[All Fields] OR "roux en y gastric bypass"[All Fields]) OR ("One"[All Fields] AND ("anastomosis, surgical"[MeSH Terms] OR ("anastomosis"[All Fields] AND "surgical"[All Fields]) OR "surgical anastomosis"[All Fields] OR "anastomosis"[All Fields]) AND ("gastric bypass"[MeSH Terms] OR ("gastric"[All Fields] AND "bypass"[All Fields]) OR "gastric bypass"[All Fields])) OR (("laparoscopes"[MeSH Terms] OR "laparoscopes"[All Fields] OR "laparoscope"[All Fields] OR "laparoscopical"[All Fields] OR "laparoscopically"[All Fields] OR "laparoscopics"[All Fields] OR "laparoscopy"[MeSH Terms] OR "laparoscopy"[All Fields] OR "laparoscopic"[All Fields]) AND ("adjustability"[All Fields] OR "adjustable"[All Fields] OR "adjustables"[All Fields] OR "adjustible"[All Fields]) AND ("gastrics"[All Fields] OR "stomach"[MeSH Terms] OR "stomach"[All Fields] OR "gastric"[All Fields]) AND ("banded"[All Fields] OR "banding"[All Fields] OR "bandings"[All Fields])) OR ("biliopancreatic diversion"[MeSH Terms] OR ("biliopancreatic"[All Fields] AND "diversion"[All Fields]) OR "biliopancreatic diversion"[All Fields]) OR (("single person"[MeSH Terms] OR ("single"[All Fields] AND "person"[All Fields]) OR "single person"[All Fields] OR "single"[All Fields] OR "singles"[All Fields]) AND ("anastomosis, surgical"[MeSH Terms] OR ("anastomosis"[All Fields] AND "surgical"[All Fields]) OR "surgical anastomosis"[All Fields] OR "anastomosis"[All Fields]) AND "Duodenal-Ileal"[All Fields] AND ("bypass"[All Fields] OR "bypassed"[All Fields] OR "bypasses"[All Fields] OR "bypassing"[All Fields])) OR (("intragastral"[All Fields] OR "intragastrally"[All Fields] OR "intragastric"[All Fields] OR "intragastrical"[All Fields] OR "intragastrically"[All Fields]) AND ("balloon"[All Fields] OR "balloon s"[All Fields] OR "balloons"[All Fields])) OR (("primaries"[All Fields] OR "primary"[All Fields]) AND ("obes surg"[Journal] OR ("obesity"[All Fields] AND "surgery"[All Fields]) OR "obesity surgery"[All Fields]) AND ("endoluminal"[All Fields] OR "endoluminally"[All Fields])) OR (("endoscope s"[All Fields] OR "endoscoped"[All Fields] OR "endoscopes"[MeSH Terms] OR "endoscopes"[All Fields] OR "endoscope"[All Fields] OR "endoscopical"[All Fields] OR "endoscopically"[All Fields] OR "endoscopy"[MeSH Terms] OR "endoscopy"[All Fields] OR "endoscopic"[All Fields]) AND ("sleeve"[All Fields] OR "sleeved"[All Fields] OR "sleeves"[All Fields] OR "sleeving"[All Fields]) AND ("gastroplasty"[MeSH Terms] OR "gastroplasty"[All Fields] OR "gastroplasties"[All Fields])) OR (("aspirant"[All Fields] OR "aspirants"[All Fields] OR "aspirate"[All Fields] OR "aspirates"[All Fields] OR "aspirating"[All Fields] OR "aspirational"[All Fields] OR "aspirations, psychological"[MeSH Terms] OR

("aspirations"[All Fields] AND "psychological"[All Fields]) OR "psychological aspirations"[All Fields] OR "aspirations"[All Fields] OR "aspirative"[All Fields] OR "aspirator"[All Fields] OR "aspirators"[All Fields] OR "aspire"[All Fields] OR "aspired"[All Fields] OR "aspires"[All Fields] OR "aspiring"[All Fields] OR "respiratory aspiration"[MeSH Terms] OR ("respiratory"[All Fields] AND "aspiration"[All Fields]) OR "respiratory aspiration"[All Fields] OR "aspirated"[All Fields] OR "aspiration"[All Fields]) AND ("therapeutics"[MeSH Terms] OR "therapeutics"[All Fields] OR "therapies"[All Fields] OR "therapy"[MeSH Subheading] OR "therapy"[All Fields] OR "therapy s"[All Fields] OR "therapys"[All Fields])) OR ("Duodenal-Jejunal"[All Fields] AND ("bypass"[All Fields] OR "bypassed"[All Fields] OR "bypasses"[All Fields] OR "bypassing"[All Fields])
Translations
obesity: "obeses"[All Fields] OR "obesity"[MeSH Terms] OR "obesity"[All Fields] OR "obese"[All Fields] OR "obesities"[All Fields] OR "obesity's"[All Fields]
overweight: "overweight"[MeSH Terms] OR "overweight"[All Fields] OR "overweighted"[All Fields] OR "overweightness"[All Fields] OR "overweights"[All Fields]
orlistat: "orlistat"[MeSH Terms] OR "orlistat"[All Fields] OR "orlistat's"[All Fields]
phentermine: "phentermine"[MeSH Terms] OR "phentermine"[All Fields]
topiramate: "topiramate"[MeSH Terms] OR "topiramate"[All Fields] OR "topiramate's"[All Fields]
naltrexone: "naltrexone"[MeSH Terms] OR "naltrexone"[All Fields] OR "naltrexon"[All Fields] OR "naltrexone's"[All Fields]
bupropion: "bupropion"[MeSH Terms] OR "bupropion"[All Fields] OR "amfebutamone"[All Fields] OR "bupropion's"[All Fields] OR "bupropione"[All Fields]
liraglutide: "liraglutid"[All Fields] OR "liraglutide"[MeSH Terms] OR "liraglutide"[All Fields] OR "liraglutide's"[All Fields]
semaglutide: "semaglutide"[Supplementary Concept] OR "semaglutide"[All Fields]
tirzepatide: "tirzepatide"[Supplementary Concept] OR "tirzepatide"[All Fields]
Sleeve: "sleeve"[All Fields] OR "sleeved"[All Fields] OR "sleeves"[All Fields] OR "sleeving"[All Fields]
Gastrectomy: "gastrectomy"[MeSH Terms] OR "gastrectomy"[All Fields] OR "gastrectomies"[All Fields]
Roux en Y Gastric Bypass: "gastric bypass"[MeSH Terms] OR ("gastric"[All Fields] AND "bypass"[All Fields]) OR "gastric bypass"[All Fields] OR "roux en y gastric bypass"[All Fields]
Anastomosis: "anastomosis, surgical"[MeSH Terms] OR ("anastomosis"[All Fields] AND "surgical"[All Fields]) OR "surgical anastomosis"[All Fields] OR "anastomosis"[All Fields]
Gastric Bypass: "gastric bypass"[MeSH Terms] OR ("gastric"[All Fields] AND "bypass"[All Fields]) OR "gastric bypass"[All Fields]
Laparoscopic: "laparoscopes"[MeSH Terms] OR "laparoscopes"[All Fields] OR "laparoscope"[All Fields] OR "laparoscopical"[All Fields] OR "laparoscopically"[All Fields] OR "laparoscopics"[All Fields] OR "laparoscopy"[MeSH Terms] OR "laparoscopy"[All Fields] OR "laparoscopic"[All Fields]
Adjustable: "adjustability"[All Fields] OR "adjustable"[All Fields] OR "adjustables"[All Fields] OR "adjustible"[All Fields]
Gastric: "gastrics"[All Fields] OR "stomach"[MeSH Terms] OR "stomach"[All Fields] OR "gastric"[All Fields]
Banding: "banded"[All Fields] OR "banding"[All Fields] OR "bandings"[All Fields]
Biliopancreatic Diversion: "biliopancreatic diversion"[MeSH Terms] OR ("biliopancreatic"[All Fields] AND "diversion"[All Fields]) OR "biliopancreatic diversion"[All Fields]
Single: "single person"[MeSH Terms] OR ("single"[All Fields] AND "person"[All Fields]) OR "single person"[All Fields] OR "single"[All Fields] OR "singles"[All Fields]
Anastomosis: "anastomosis, surgical"[MeSH Terms] OR ("anastomosis"[All Fields] AND "surgical"[All Fields]) OR "surgical anastomosis"[All Fields] OR "anastomosis"[All Fields]
bypass: "bypass"[All Fields] OR "bypassed"[All Fields] OR "bypasses"[All Fields] OR "bypassing"[All Fields]
Intragastric: "intragastral"[All Fields] OR "intragastrally"[All Fields] OR "intragastric"[All Fields] OR "intragastrical"[All Fields] OR "intragastrically"[All Fields]
Balloons: "balloon"[All Fields] OR "balloon's"[All Fields] OR "balloons"[All Fields]
Primary: "primaries"[All Fields] OR "primary"[All Fields]
Obesity Surgery: "Obes Surg"[Journal:__jid9106714] OR ("obesity"[All Fields] AND "surgery"[All Fields]) OR "obesity surgery"[All Fields]
Endoluminal: "endoluminal"[All Fields] OR "endoluminally"[All Fields]
Endoscopic: "endoscope's"[All Fields] OR "endoscoped"[All Fields] OR "endoscopes"[MeSH Terms] OR "endoscopes"[All Fields] OR "endoscope"[All Fields] OR "endoscopical"[All Fields] OR "endoscopically"[All Fields] OR "endoscopy"[MeSH Terms] OR "endoscopy"[All Fields] OR

"endoscopic"[All Fields]
Sleeve: "sleeve"[All Fields] OR "sleeved"[All Fields] OR "sleeves"[All Fields] OR "sleeving"[All Fields]
Gastroplasty: "gastroplasty"[MeSH Terms] OR "gastroplasty"[All Fields] OR "gastroplasties"[All Fields]
aspiration: "aspirant"[All Fields] OR "aspirants"[All Fields] OR "aspirate"[All Fields] OR "aspirates"[All Fields] OR "aspirating"[All Fields] OR "aspirational"[All Fields] OR "aspirations, psychological"[MeSH Terms] OR ("aspirations"[All Fields] AND "psychological"[All Fields]) OR "psychological aspirations"[All Fields] OR "aspirations"[All Fields] OR "aspirative"[All Fields] OR "aspirator"[All Fields] OR "aspirators"[All Fields] OR "aspire"[All Fields] OR "aspired"[All Fields] OR "aspires"[All Fields] OR "aspiring"[All Fields] OR "respiratory aspiration"[MeSH Terms] OR ("respiratory"[All Fields] AND "aspiration"[All Fields]) OR "respiratory aspiration"[All Fields] OR "aspirated"[All Fields] OR "aspiration"[All Fields]
therapy: "therapeutics"[MeSH Terms] OR "therapeutics"[All Fields] OR "therapies"[All Fields] OR "therapy"[Subheading] OR "therapy"[All Fields] OR "therapy's"[All Fields] OR "therapys"[All Fields]
Bypass: "bypass"[All Fields] OR "bypassed"[All Fields] OR "bypasses"[All Fields] OR "bypassing"[All Fields]

EMBASE
N= 2,106
('obesity'/exp OR obesity OR 'overweight'/exp OR overweight) AND ('orlistat'/exp OR orlistat OR 'phentermine'/exp OR phentermine OR 'topiramate'/exp OR topiramate OR 'naltrexone'/exp OR naltrexone OR 'bupropion'/exp OR bupropion OR 'liraglutide'/exp OR liraglutide OR 'semaglutide'/exp OR semaglutide OR 'tirzepatide'/exp OR tirzepatide OR 'sleeve gastrectomy'/exp OR 'sleeve gastrectomy' OR (sleeve AND ('gastrectomy'/exp OR gastrectomy)) OR 'roux en y gastric bypass'/exp OR 'roux en y gastric bypass' OR (roux AND ('en'/exp OR en) AND y AND gastric AND ('bypass'/exp OR bypass)) OR 'one anastomosis gastric bypass'/exp OR 'one anastomosis gastric bypass' OR (('one'/exp OR one) AND ('anastomosis'/exp OR anastomosis) AND gastric AND ('bypass'/exp OR bypass)) OR 'laparoscopic adjustable gastric banding'/exp OR 'laparoscopic adjustable gastric banding' OR (laparoscopic AND adjustable AND gastric AND banding) OR 'biliopancreatic diversion'/exp OR 'biliopancreatic diversion' OR (biliopancreatic AND ('diversion'/exp OR diversion)) OR 'single anastomosis duodenal-ileal bypass' OR (single AND ('anastomosis'/exp OR anastomosis) AND 'duodenal ileal' AND ('bypass'/exp OR bypass)) OR 'intragastric balloons' OR (intragastric AND balloons) OR 'primary obesity surgery endoluminal'/exp OR 'primary obesity surgery endoluminal' OR (primary AND ('obesity'/exp OR obesity) AND ('surgery'/exp OR surgery) AND endoluminal) OR 'endoscopic sleeve gastroplasty'/exp OR 'endoscopic sleeve gastroplasty' OR (endoscopic AND sleeve AND ('gastroplasty'/exp OR gastroplasty)) OR 'aspiration therapy'/exp OR 'aspiration therapy' OR (('aspiration'/exp OR aspiration) AND ('therapy'/exp OR therapy)) OR ('duodenal jejunal' AND ('bypass'/exp OR bypass)) AND [embase]/lim NOT ([embase]/lim AND [medline]/lim) AND ('controlled clinical trial'/de OR 'double blind procedure'/de OR 'phase 2 clinical trial topic'/de OR 'phase 3 clinical trial'/de OR 'phase 3 clinical trial topic'/de OR 'randomized controlled trial'/de OR 'randomized controlled trial topic'/de)

Additional search:
Additional manual search of the references of included trials and former meta-analyses was carried out to identify other newly published and unpublished studies. Completed but yet unpublished studies with the procedures specified above were searched in the www.clinicaltrials.gov register. using the same search string as above.

Table 3S: Information collected for each trial

First author
Publication year
National Clinical Trial (NCT) number or other registration identifiers/acronyms
Pharmacological (doses), surgical, and endoscopic procedures
Sample size
Duration of the trial
Minimum and maximum body mass index (BMI)
Minimum and maximum age
Baseline BMI
Final BMI (± SD)
Weight (± SD)
Total Weight Lost (%)
Any Serious adverse events (SAE)
All-cause mortality
Quality of life and treatment satisfaction

Table 4S. Excluded trials and reasons for the exclusion.

N	Study	Publication year	Reason for the exclusion
1	De Moura	2019	Short treatment/follow-up period
2	Pajecki	2023	Duplicate
3	Aldhwayan	2022	Duplicate
4	Newsome	2021	Submaximal doses
5	De Moura	2019	Not approved intervention for obesity
6	Fidler	2011	Not approved intervention for obesity
7	Bohula	2018	Not approved intervention for obesity
8	O'Neil	2012	Not approved intervention for obesity
9	Scirica	2019	Not approved intervention for obesity
10	Smith	2010	Not approved intervention for obesity
11	Mittemair	2007	Not approved intervention for obesity
12	Horwitz	2014	Not RCT
13	Mollan	2021	Same type of surgical procedure in both arms
14	Inagaki	2022	Not on obesity

Table 5S. Principal baseline characteristics of the included studies

n	Study Name	Intervention	Comparator	N. pat. Interv.	N. pat. Comp.	Trial duration (weeks)	BMI min. (kg/m^2)	BMI max. (kg/m^2)	Age max. (years)	BMI (kg/m^2)	Age (years)	Women (%)	DM (%)
1	*Astrup*[1]	Liraglutide	Orlistat	93	95	52	30	40	65	34.4	46	75	3
			Placebo		98	52	30	40	65	34.8	46	75	4
2	*Gudbergsen*[2]	Liraglutide	Placebo	80	76	52	27	NR	74	32.1	59	65	NR
3	*Lundgren*[3]	Liraglutide	Placebo	98	97	52	32	43	65	32.6	43	64	0
4	*O'Neil*[4]	Liraglutide	Placebo	103	136	52	30	NR	NR	39.3	47	35	0
5	*Davies*[5]	Liraglutide	Placebo	423	212	56	27	NR	NR	37.2	55	51	100
6	*Garvey*[6]	Liraglutide	Placebo	195	197	56	27	NR	NR	35.5	57	53	100
7	*Wadden*[7]	Liraglutide	Placebo	212	210	56	27	NR	NR	35.6	46	81	0
8	*Wadden*[8]	Liraglutide	Placebo	142	140	56	30	NR	NR	39.0	47	84	0
9	*Le Roux*[9]	Liraglutide	Placebo	1505	749	156	27	NR	NR	38.9	47	76	0
10	*Apovian*[10]	Naltr./Bupr.	Placebo	992	492	56	30	45	65	36.1	44	85	0
11	*Greenway*[11]	Naltr./Bupr.	Placebo	583	581	56	27	45	65	36.1	44	85	0
12	*Hollander*[12]	Naltr./Bupr.	Placebo	335	170	56	27	45	70	36.4	54	55	100
13	*Wadden*[13]	Naltr./Bupr.	Placebo	591	202	56	27	45	65	36.5	46	90	0
14	*Nissen*[14]	Naltr./Bupr.	Placebo	4455	4450	156	27	50	NR	36.6	61	54	85
15	*Bakris*[15]	Orlistat	Placebo	267	265	52	28	43	NR	35.6	53	61	8
16	*Berne*[16]	Orlistat	Placebo	111	109	52	28	40	75	32.7	59	45	100
17	*Davidson*[17]	Orlistat	Placebo	657	223	52	30	43	NR	36.3	44	84	0
18	*Derosa*[18]	Orlistat	Placebo	126	128	52	30	NR	NR	32.8	52	50	100
19	*Derosa*[19]	Orlistat	Placebo	53	47	52	30	NR	NR	32.1	51	52	0
20	*Finer*[20]	Orlistat	Placebo	110	108	52	30	43	NR	36.8	41	89	0
21	*Hill*[21]	Orlistat	Placebo	179	184	52	28	34	NR	32.8	46	85	0
22	*James*[22]	Orlistat	Placebo	23	23	52	30	43	NR	37.5	43	85	0
23	*Karhunen*[23]	Orlistat	Placebo	36	36	52	30	43	NR	35.8	44	77	0
24	*Kelley*[24]	Orlistat	Placebo	266	269	52	28	40	65	35.7	58	56	100
25	*Miles*[25]	Orlistat	Placebo	250	254	52	28	43	65	35.4	53	48	100

26	*Poston*[26]	Orlistat	Placebo	37	35	52	27	NR	65	36.8	43	100	11
27	*Sjostrom*[27]	Orlistat	Placebo	343	340	52	28	47	NR	36.0	45	83	NR
28	*Svendsen*[28]	Orlistat	Placebo	23	21	52	30	NR	63	37.5	48	55	NR
29	*Swinburn*[29]	Orlistat	Placebo	170	169	52	30	50	70	37.8	52	57	8
30	*Zavoral*[30]	Orlistat	Placebo	1561	1119	52	28	43	NR	35.0	44	84	4
31	*Hollander*[31]	Orlistat	Placebo	162	159	57	28	40	NR	34.2	55	50	100
32	*Krempf*[32]	Orlistat	Placebo	346	350	78	28	NR	65	36.1	41	86	0
33	*Hauptman*[33]	Orlistat	Placebo	210	212	104	30	44	NR	36.0	42	68	NR
34	*Rossner*[34]	Orlistat	Placebo	244	243	104	28	43	NR	35.0	44	85	NR
35	*Richelsen*[35]	Orlistat	Placebo	153	156	156	30	45	65	37.5	47	51	20
36	*Togerson*[36]	Orlistat	Placebo	1640	1637	208	30	NR	60	37.3	43	55	0
37	*Allison*[37]	Phen./Topir.	Placebo	512	514	56	35	NR	70	42.2	43	83	NR
38	*Gadde*[38]	Phen./Topir.	Placebo	995	994	56	27	45	70	36.6	51	70	67
39	*Kosiborod*[39]	Semaglutide	Placebo	310	306	52	30	NR	NR	36.9	70	44	100
40	*Kosiborod*[40]	Semaglutide	Placebo	263	266	52	30	NR	NR	37.0	69	56	0
41	*McGowan*[41]	Semaglutide	Placebo	138	69	52	30	NR	NR	40.1	53	71	0
42	*Bliddal*[42]	Semaglutide	Placebo	271	136	68	30	NR	NR	40.3	56	82	0
43	*Davies*[43]	Semaglutide	Placebo	404	403	68	27	NR	NR	35.9	55	51	100
44	*Kadowaki*[44]	Semaglutide	Placebo	199	101	68	27	NR	NR	31.9	51	37	25
45	*Rubino*[45]	Semaglutide	Placebo	535	268	68	27	NR	NR	34.3	46	78	0
46	*Wadden*[46]	Semaglutide	Placebo	407	204	68	27	NR	NR	38.0	46	82	0
47	*Wilding*[47]	Semaglutide	Placebo	1306	655	68	27	NR	NR	37.9	46	75	0
48	*Garvey*[48]	Semaglutide	Placebo	152	152	104	27	NR	NR	38.5	47	77	0
49	*Lincoff*[49]	Semaglutide	Placebo	8803	8801	104	27	NR	NR	33.3	62	28	0
50	*Aronne*[50]	Tirzepatide	Placebo	335	335	52	27	NR	NR	30.5	49	70	0
51	*Loomba* [51]	Tirzepatide	Placebo	95	48	52	27	50	80	36.1	54	57	58
52	*Malhotra*[52]	Tirzepatide	Placebo	234	235	52	30	NR	NR	39.0	50	30	0
53	*Garvey*[53]	Tirzepatide	Placebo	623	315	72	27	NR	NR	36.1	54	51	100
54	*Jastreboff*[54]	Tirzepatide	Placebo	1266	643	72	27	NR	NR	38.0	45	67	0
	**Total (mean value) in trials on OMM**			**33,622**	**28,339**	**68**	**29**	**44**	**67**	**36.1**	**50**	**66**	**28**

1	*Sullivan*[55]	Aspiration	Lifestyle	10	4	52	35	50	NR	41.0	40	90	NR
2	*Thompson*[56]	Aspiration	No therapy	111	60	52	35	55	65	41.4	45	87	6
3	*Abu Dayyeh*[63]	ESG	No therapy	77	110	52	30	40	65	35.6	47	85	25
4	*Coffin*[64]	IGB	Lifestyle	55	60	26	45	NR	65	54.3	40	73	30
5	*Genco*[65]	IGB	Lifestyle	25	25	26	40	45	35	41.2	31	70	NR
6	*Dargent*[66]	IGB	No therapy	68	32	24	27	NR	65	34.1	37	90	4
7	*Courcoulas*[67]	IGB	No therapy	135	136	26	30	40	65	34.9	39	90	7
8	*Fuller*[68]	IGB	No therapy	37	37	26	30	40	60	36.3	45	67	NR
9	*Ponce*[69]	IGB	No therapy	21	9	26	30	40	60	34.9	42	90	NR
10	*Abu Dayyeh*[70]	IGB	No therapy	187	101	32	30	40	65	35.8	44	88	NR
11	*Ponce*[69]	IGB	Placebo	187	139	24	30	40	60	35.3	44	95	7
12	*Lee*[71]	IGB	Placebo	8	10	26	27	NR	65	31.3	45	40	11
13	*Sullivan*[72]	IGB	Placebo	198	189	26	30	40	64	35.3	43	87	0
14	*Miller*[73]	POSE	No therapy	30	9	52	30	40	60	36.5	39	67	5
15	*Sullivan*[74]	POSE	Placebo	221	111	52	30	40	60	36.1	45	89	9
**Total (mean value) in trials on EP**				**1,649**	**1,285**	**36**	**31**	**44**	**61**	**37.1**	**44**	**75**	**41**

1	*Feigel-Guiller*[75]	LAGB	Lifestyle	5	24	156	35	NR	65	47.0	48	67	100
2	*Simonson*[76]	LAGB	Lifestyle	23	22	156	30	45	65	36.5	51	45	100
3	*O'Brien*[77]	LAGB	Lifestyle	40	10	520	30	35	50	33.6	41	86	NR
4	*Morino*[78]	LAGB	LVGB	49	51	156	40	50	60	44.3	38	81	29
5	*Dowsey*[79]	LAGB	No therapy	41	41	52	35	NR	65	43.7	58	80	100
6	*Dixon*[80]	LAGB	No therapy	30	30	104	30	40	60	37.1	47	54	33
7	*Dixon*[81]	LAGB	No therapy	30	30	104	35	55	60	45.0	48	42	42
8	*Xiang*[82]	LAGB	No therapy	44	44	104	30	40	65	35.3	34	78	7
9	*Nilsell*[83]	LVBG	LAGB	30	29	260	37	NR	60	43.4	39	81	3
10	*Scozzari*[84]	LVGB	LAGB	51	49	364	40	50	60	44.5	38	81	8
11	*Lundell*[85]	LVGB	LAGB	24	26	52	35	NR	NR	42.5	48	54	
12	*Darabi*[86]	OAGB	GCP	20	20	52	35	NR	65	48.5	35	70	NR
13	*Musella*[87]	OAGB	SG	32	32	52	35	NR	65	48.0	NR	NR	40

14	*Roushdy*[88]	OAGB	SG	21	21	52	35	NR	60	50.2	34	95	4
15	*Hany*[89]	OAGB	SG	150	150	260	35	NR	60	44.0	34	77	48
16	*Jain*[90]	OAGB	SG	101	100	260	35	60	60	44.4	42	36	100
17	*Lee*[91]	OAGB	SG	30	30	260	27	35	60	30.6	45	71	17
18	*Hedberg*[92]	RYGB	BPD	23	24	208	48	NR	NR	54.5	39	49	13
19	*Skroubis*[93]	RYGB	BPD	65	65	416	35	50	NR	44.9	35	52	100
20	*Mingrone*[94]	RYGB	BPD	20	20	520	35	NR	60	45.0	44	55	18
			No therapy		20	520	35	NR	60	45.0	44	50	22
		BPD	No therapy	20	20	520	35	NR	60	45.0	44	55	31
21	*Salte* [95]	RYGB	BPD	31	29	520	50	60	50	55.0	36	70	3
22	*Hall*[96]	RYGB	Gastrogastrost.	99	105	156	30	NR	NR	NR	34	95	100
			LVGB		106	156	30	NR	NR	NR	34	95	NR
		LVBG	Gastrogastrost.	106	105	156	30	NR	NR	NR	34	95	NR
23	*Casajoana*[97]	RYGB	GCP	15	15	260	35	43	60	39.5	49	66	100
			SG		15	260	35	43	60	39.5	49	60	8
		SG	GCP	15	15	260	35	43	60	39.5	49	72	56
24	*Nguyen*[98]	RYGB	LAGB	111	86	520	35	60	60	46.5	43	76	100
25	*Courcolas*[99]	RYGB	Lifestyle	20	20	260	30	40	55	35.7	47	82	100
			LAGB		21	260	30	40	55	35.7	47	82	24
		LAGB	Lifestyle	21	20	260	30	40	55	35.7	47	82	NR
26	*Cohen*[100]	RYGB	Lifestyle	51	49	104	30	35	65	32.5	51	45	100
27	*Cheng*[101]	RYGB	Lifestyle	12	14	260	27	32	65	29.0	44	35	100
28	*Schauer*[102]	RYGB	Lifestyle	50	50	260	27	43	60	36.7	49	66	NR
			SG		50	260	27	43	60	36.7	49	66	27
		SG	Lifestyle	50	50	260	27	43	60	36.7	49	66	6
29	*Lee*[103]	RYGB	LVGB	40	40	104	35	60	60	43.1	32	70	Nr
30	*Olbers*[104]	RYGB	LVGB	37	46	104	35	50	NR	42.0	35	73	100
31	*Werling*[105]	RYGB	LVGB	37	46	312	35	50	NR	42.3	45	73	3
32	*MacLean*[106]	RYGB	LVGB	52	54	156	40	50	NR	49.0	39	NR	100
33	*Cummings*[107]	RYGB	No therapy	15	17	52	30	40	65	38.3	53	69	100

34	*Liang*[108]	RYGB	No therapy	31	70	52	28	NR	30	30.4	51	33	8
35	*Schiavon*[109]	RYGB	No therapy	50	50	156	30	40	65	36.9	44	76	100
36	*Ikramuddin*[110]	RYGB	No therapy	57	56	260	30	40	67	34.6	48	60	100
37	*Delko*[111]	RYGB	OAGB	40	40	52	35	50	NR	39.8	40	89	35
38	*Eskandaros* [112]	RYGB	OAGB	40	40	52	35	NR	60	50.0	36	51	60
39	*Karagul*[113]	RYGB	OAGB	20	22	156	35	NR	65	45.5	43	82	100
40	*Singh*[114]	RYGB	OAGB	24	25	208	30	NR	NR	45.8	46	69	11
41	*Level*[115]	RYGB	OAGB	9	19	260	35	NR	NR	42.7	37	NR	27
42	*Robert*[116]	RYGB	OAGB	117	117	260	35	NR	65	43.9	43	75	22
43	*Biter*[117]	RYGB	SG	74	76	52	35	NR	NR	44.1	44	82	6
44	*Karamanakos*[118]	RYGB	SG	16	16	52	30	NR	NR	45.6	33	84	100
45	*Keidar*[119]	RYGB	SG	19	18	52	35	NR	65	42.2	49	46	35
46	*Paluszkiewicz*[120]	RYGB	SG	36	36	52	35	60	60	47.3	44	67	27
47	*Ramon*[121]	RYGB	SG	7	8	52	35	50	60	43.8	48	NR	35
48	*Verrastro*[122]	RYGB	SG	77	79	52	30	55	70	42.1	47	47	100
49	*Tang*[123]	RYGB	SG	40	40	104	28	NR	65	38.1	38	57	100
50	*Wallenius*[124]	RYGB	SG	25	24	104	35	60	60	40.2	48	47	75
51	*Pajecki*[125]	RYGB	SG	18	18	152	35	NR	NR	45.0	68	85	23
52	*Catheline*[126]	RYGB	SG	91	186	156	35	NR	60	45.3	41	86	17
53	*Kehagias*[127]	RYGB	SG	30	30	156	30	50	NR	45.4	35	36	100
54	*Svanevik*[128]	RYGB	SG	54	55	156	35	NR	NR	42.2	48	66	100
55	*Yang*[129]	RYGB	SG	32	32	156	28	35	60	32.0	41	66	100
56	*Ignat*[130]	RYGB	SG	45	55	260	40	60	60	46.3	35	82	25
57	*Peterli*[131]	RYGB	SG	110	107	260	35	NR	65	43.9	42	72	100
58	*Zhang*[132]	RYGB	SG	32	32	260	32	50	60	39.0	31	59	100
59	*Pullman*[133]	RYGB	SG	56	58	364	35	65	55	42.1	47	52	100
60	*Salminen*[134]	RYGB	SG	49	52	520	35	60	60	45.9	48	69	18
61	*Axer*[135]	SADI	BPD	30	30	52	42	72	NR	50.0	40	50	23
62	*Talebpour*[136]	SG	GCP	35	35	104	35	NR	65	46.0	37	80	9
63	*Grubnik*[137]	SG	GCP	27	25	156	35	65	75	43.7	42	76	100
64	*Spaggiari*[138]	SG	Lifestyle	11	9	52	35	NR	NR	41.8	45	56	100

**Total (mean value) in trials on MBS**	**2,930**	**3,061**	**201**	**34**	**49**	**61**	**42.1**	**43**	**67**	**56**

N.: Number; **Min.;** Minimum; **Max.:** Maximum; **BMI:** Body Mass Index; **DM:** Proportion of subjects with diabetes enrolled; **Naltr./Bupr.**: Naltrexone/Bupropion; **Phen./Topir.**: Phentermine/Topiramate; **LAGB**: Laparoscopic Adjustable Gastric Banding; **LVBG**: Vertical Banding Gastroplasty; **VG**: Vertical Gastrogastrostomy; **SG**: Sleeve Gastrectomy; **RYGB**: Roux-en-Y Gastric By-Pass; **BPD**: Bilio-Pancreatic Diversion; **GCP**: Greater Curvature Plication Gastric; **OAGB**: One-anastomosis gastric bypass; **NR**: Not Reported. **OMM**: Obesity Management Medications; **EP**: Endoscopic Procedures; **MBS**: Metabolic Bariatric Surgery.

Table 6S – Direct and estimates of effects of different anti-obesity strategies on 26-52 weeks TBWL%.

ID	Comparison	Active	Control	WMD	LCI 95%	UCI 95%
	Direct estimates					
1	AT vs LSI	Aspiration	LSI	9.18	5.38	12.97
2	BPD vs LSI	Biliopancreatic Diversion	LSI	25.90	20.41	31.39
3	IGB vs LSI	Intragastric Balloon	LSI	4.77	1.90	7.63
4	LAGB vs LSI	Laparoscopic Adjustable Gastric Binding	LSI	13.23	5.34	21.12
5	Liraglutide vs LSI	Liraglutide 3.0 mg	LSI	4.38	3.64	5.13
6	Nalt/Bup vs LSI	Naltrexone SR/Bupropion	LSI	4.76	3.73	5.80
7	OAGB vs GCP	One-Anastomosis Gastric Bypass	GCP	3.06	1.09	5.03
8	OAGB vs SG	One-Anastomosis Gastric Bypass	SG	2.40	-2.94	7.74
9	Orlistat vs LSI	Orlistat 360 mg	LSI	3.14	2.72	3.57
10	Phen/Topir vs LSI	Phentermine/Topiramate (15/92)	LSI	8.85	8.19	9.52
11	POSE vs LSI	POSE	LSI	3.76	-0.34	7.86
12	RYGB vs BPD	Rou-en-Y Gastric Bypass	BPD	-12.48	-27.47	2.52
13	RYGB vs LAGB	Rou-en-Y Gastric Bypass	LAGB	11.74	8.77	14.72
14	RYGB vs LVBG	Rou-en-Y Gastric Bypass	LVBG	5.10	1.64	8.56
15	RYGB vs LSI	Rou-en-Y Gastric Bypass	LSI	16.15	11.73	20.58
16	RYGB vs OAGB	Rou-en-Y Gastric Bypass	OAGB	-3.51	-7.26	0.24
17	RYGB vs SG	Rou-en-Y Gastric Bypass	SG	5.09	1.99	8.20
18	Semaglutide vs LSI	Semaglutide 2.4 mg	LSI	8.64	5.95	11.33
19	SG vs GCP	Sleeve Gastrectomy	LSI	4.00	0.92	7.08
20	SG vs LSI	Sleeve Gastrectomy	LSI	12.97	11.22	14.72
21	Tirzepatide vs LSI	Tirzepatide 10-15 mg	LSI	15.25	11.88	18.62
22	SADI vs BPD	Single Anastomosis Duodenal Switch	BPD	-1.50	-4.61	1.61
	**Indirect estimates** (source IDs)					
23	One-Anastomosis Gastric Bypass vs LSI (9, 22)	One-Anastomosis Gastric Bypass	LSI	16.03	13.39	18.67
24	Rou-en-Y Gastric Bypass vs LSI (14, 2)	Rou-en-Y Gastric Bypass	LSI	13.42	-2.55	29.39
25	Rou-en-Y Gastric Bypass vs LSI (15, 5)	Rou-en-Y Gastric Bypass	LSI	24.98	16.55	33.41
26	Rou-en-Y Gastric Bypass vs LSI (19, 22)	Rou-en-Y Gastric Bypass	LSI	18.07	14.50	21.63
27	Single Anastomosis Duodenal Switch vs LSI (24, 2)	Single Anastomosis Duodenal Switch	LSI	24.40	18.09	30.71
28	Biliopancreatic Diversion vs LSI (14, 17)	Biliopancreatic Diversion	LSI	28.63	13.00	44.26
29	Laparoscopic Adjustable Gastric Binding vs LSI (15, 17)	Laparoscopic Adjustable Gastric Binding	LSI	4.41	-0.92	9.74
30	Laparoscopic Vertical Banded Gastroplasty vs LSI (16, 17)	Laparoscopic Vertical Banded Gastroplasty	LSI	11.05	5.44	16.67

31	Greater Curvature Plication vs LSI (21, 22)	Greater Curvature Plication	LSI	8.97	5.43	12.51
32	One-Anastomosis Gastric Bypass vs LSI (18, 17)	One-Anastomosis Gastric Bypass	LSI	19.66	13.86	25.47
33	Sleeve Gastrectomy vs LSI (19, 17)	Sleeve Gastrectomy	LSI	11.06	5.65	16.47
	**Result estimates** (source IDs)		LSI			
	Aspiration (1)	Aspiration	LSI	9.18	5.38	12.97
	Biliopancreatic Diversion (2, 30)	Biliopancreatic Diversion	LSI	26.20	21.02	31.38
	Intragastric Balloon (4)	Intragastric Balloon	LSI	4.77	1.90	7.63
	Laparoscopic Adjustable Gastric Binding (5, 31)	Laparoscopic Adjustable Gastric Binding	LSI	7.18	-1.70	16.06
	Liraglutide 3.0 mg (6)	Liraglutide 3.0 mg	LSI	4.38	3.64	5.13
	Naltrexone SR/Bupropion (8)	Naltrexone SR/Bupropion	LSI	4.76	3.73	5.80
	One-Anastomosis Gastric Bypass (25, 34)	One-Anastomosis Gastric Bypass	LSI	16.65	13.59	19.71
	Sleeve Gastrectomy (22, 35)	Sleeve Gastrectomy	LSI	12.79	11.12	14.46
	Greater Curvature Plication (33)	Greater Curvature Plication	LSI	8.97	5.43	12.51
	Orlistat (11)	Orlistat	LSI	3.14	2.72	3.57
	Phentermine/Topiramate (15/92) (12)	Phentermine/Topiramate (15/92)	LSI	8.85	8.19	9.52
	POSE (13)	POSE	LSI	3.76	-0.34	7.86
	Rou-en-Y Gastric Bypass (17, 26-28)	Rou-en-Y Gastric Bypass	LSI	17.94	14.83	21.05
	Laparoscopic Vertical Banded Gastroplasty (32)	Laparoscopic Vertical Banded Gastroplasty	LSI	11.05	5.44	16.67
	Semaglutide 2.4 mg (20)	Semaglutide 2.4 mg	LSI	8.64	5.95	11.33
	Tirzepatide 10-15 mg (23)	Tirzepatide 10-15 mg	LSI	15.25	11.88	18.62
	SADI (29)	SADI	LSI	24.40	18.09	30.71

Naltr./Bupr.: Naltrexone/Bupropion; **Phen./Topir.**: Phentermine/Topiramate; **POSE**: Primary Obesity Surgery Endoluminal; **IGB**: Intra-Gastric Balloon; **AT**: Aspiration Therapy; **ESG**: Endoscopic Sleeve Gastroplasty; **LAGB**: Laparoscopic Adjustable Gastric Banding; **GCP**: Greater Curvature Plication Gastric; **LVGB**: Laparoscopic Vertical Banded Gastroplasty; **SG**: Sleeve Gastrectomy; **OAGB**: One-anastomosis gastric bypass; **RYGB**: Roux-en-Y Gastric By-Pass; **SADI**: Single Anastomosis Duodenal Switch; **BPD**: Bilio-Pancreatic Diversion. **LSI:** Lifestyle/Placebo/No therapy. **WMD**: Weighted Mean Difference; **LCI** and **UCI**: Lower and Upper Confidence Intervals.

Table 7S – Direct and estimates of effects of different anti-obesity strategies on 53-104 weeks TBWL%.

ID	Comparison	Active	Control	WMD	LCI 95%	UCI 95%
	Direct estimates					
1	BPD vs LSI	Biliopancreatic Diversion	LSI	23.98	18.49	29.47
2	LAGB vs LSI	Laparoscopic Adjustable Gastric Binding	LSI	13.46	6.10	20.82
3	Liraglutide vs LSI	Liraglutide 3.0 mg	LSI	5.10	4.52	5.68
4	OAGB vs SG	One-Anastomosis Gastric Bypass	SG	2.76	0.34	5.18
5	Orlistat vs LSI	Orlistat 360 mg	LSI	3.16	2.30	4.02
6	RYGB vs BPD	Rou-en-Y Gastric Bypass	BPD	-11.11	-23.95	1.74
7	RYGB vs LAGB	Rou-en-Y Gastric Bypass	LAGB	8.87	5.41	12.33
8	RYGB vs LVBG	Rou-en-Y Gastric Bypass	LVBG	3.77	0.30	7.23
9	RYGB vs LSI	Rou-en-Y Gastric Bypass	LSI	15.35	9.60	21.10
10	RYGB vs OAGB	Rou-en-Y Gastric Bypass	OAGB	1.49	-0.50	3.48
11	Semaglutide vs LSI	Semaglutide 2.4 mg	LSI	10.53	4.41	16.66
12	SG vs GCP	Sleeve Gastrectomy	GCP	4.00	0.92	7.08
13	Tirzepatide vs LSI	Tirzepatide 10-15 mg	LSI	16.47	8.21	24.73
14	RYGB vs SG	Rou-en-Y Gastric Bypass	SG	3.26	0.86	5.66
	**Indirect estimates** (source IDs)					
15	Rou-en-Y Gastric Bypass vs LSI (6, 1)	Rou-en-Y Gastric Bypass	LSI	12.87	-1.10	26.84
16	Rou-en-Y Gastric Bypass vs LSI(7, 2)	Rou-en-Y Gastric Bypass	LSI	22.33	14.20	30.46
17	Biliopancreatic Diversion vs LSI (6, 9)	Biliopancreatic Diversion	LSI	26.45	12.38	40.52
18	Laparoscopic Adjustable Gastric Binding vs LSI (7, 9)	Laparoscopic Adjustable Gastric Binding	LSI	6.47	-0.23	13.18
19	Laparoscopic Vertical Banded Gastroplasty vs LSI (8, 9)	Laparoscopic Vertical Banded Gastroplasty	LSI	11.58	4.87	18.29
20	One-Anastomosis Gastric Bypass vs LSI (10, 9)	One-Anastomosis Gastric Bypass	LSI	13.86	7.77	19.94
21	Sleeve Gastrectomy vs LSI (14, 9)	Sleeve Gastrectomy	LSI	12.09	5.86	18.32
22	Greater Curvature Plication vs LSI (12, 21)	Greater Curvature Plication	LSI	8.09	1.14	15.03
	**Result estimates** (source IDs)					
	Biliopancreatic Diversion (1, 17)	Biliopancreatic Diversion	LSI	24.31	19.19	29.42
	Laparoscopic Adjustable Gastric Binding (2, 18)	Laparoscopic Adjustable Gastric Binding	LSI	9.65	2.80	16.49
	Liraglutide 3.0 mg (3)	Liraglutide 3.0 mg	LSI	5.10	4.52	5.68
	One-Anastomosis Gastric Bypass (20)	One-Anastomosis Gastric Bypass	LSI	13.86	7.77	19.94
	Sleeve Gastrectomy (21)	Sleeve Gastrectomy	LSI	12.09	5.86	18.32

	Orlistat (5)	Orlistat	LSI	3.16	2.30	4.02
	Rou-en-Y Gastric Bypass (9, 15, 16)	Rou-en-Y Gastric Bypass	LSI	17.19	12.21	22.17
	Laparoscopic Vertical Banded Gastroplasty (19)	Laparoscopic Vertical Banded Gastroplasty	LSI	11.58	4.87	18.29
	Semaglutide 2.4 mg (11)	Semaglutide 2.4 mg	LSI	10.53	4.41	16.66
	Greater Curvature Plication (22)	Greater Curvature Plication	LSI	8.09	1.14	15.03
	Tirzepatide 10-15 mg (13)	Tirzepatide 10-15 mg	LSI	16.47	8.21	24.73

Naltr./Bupr.: Naltrexone/Bupropion; **Phen./Topir.**: Phentermine/Topiramate; **POSE**: Primary Obesity Surgery Endoluminal; **IGB**: Intra-Gastric Balloon;**AT**: Aspiration Therapy; **ESG**: Endoscopic Sleeve Gastroplasty; **LAGB**: Laparoscopic Adjustable Gastric Banding; **GCP**: Greater Curvature Plication Gastric; **LVGB**: Laparoscopic Vertical Banded Gastroplasty; **SG**: Sleeve Gastrectomy; **OAGB**: One-anastomosis gastric bypass; **RYGB**: Roux-en-Y Gastric By-Pass; **SADI**: Single Anastomosis Duodenal Switch; **BPD**: Bilio-Pancreatic Diversion. **LSI:** Lifestyle/Placebo/No therapy. **WMD**: Weighted Mean Difference; **LCI** and **UCI**: Lower and Upper Confidence Intervals.

Table 8S – Direct and estimates of effects of different anti-obesity strategies on 105-156 weeks TBWL%.

ID	Comparison	Active	Control	WMD	LCI 95%	UCI 95%
	Direct estimates					
1	BPD vs LSI	Biliopancreatic Diversion	LSI	24.10	18.61	29.59
2	LAGB vs LSI	Laparoscopic Adjustable Gastric Binding	LSI	7.21	4.99	9.44
3	Liraglutide vs LSI	Liraglutide 3.0 mg	LSI	4.20	3.62	4.78
4	OAGB vs SG	One-Anastomosis Gastric Bypass	SG	3.77	1.35	6.19
5	Orlistat vs LSI	Orlistat	LSI	3.00	2.59	3.41
6	RYGB vs BPD	Rou-en-Y Gastric Bypass	BPD	-10.07	-17.35	-2.79
7	RYGB vs LAGB	Rou-en-Y Gastric Bypass	LAGB	10.02	6.56	13.48
8	RYGB vs LVBG	Rou-en-Y Gastric Bypass	LVBG	4.02	0.56	7.48
9	RYGB vs LSI	Rou-en-Y Gastric Bypass	LSI	23.46	11.94	34.97
10	RYGB vs OAGB	Rou-en-Y Gastric Bypass	OAGB	0.96	-2.70	4.61
11	Semaglutide vs LSI	Semaglutide 2.4 mg	LSI	8.72	6.97	10.46
12	RYGB vs SG	Rou-en-Y Gastric Bypass	SG	4.10	1.33	6.87
	**Indirect estimates** (source IDs)					
13	Rou-en-Y Gastric Bypass vs LSI (6, 1)	Rou-en-Y Gastric Bypass	LSI	14.03	4.91	23.15
14	Rou-en-Y Gastric Bypass vs LSI (7, 2)	Rou-en-Y Gastric Bypass	LSI	17.23	13.12	21.35
15	Biliopancreatic Diversion vs LSI (6, 9)	Biliopancreatic Diversion	LSI	33.52	19.90	47.14
16	Laparoscopic Adjustable Gastric Binding vs LSI (7, 9)	Laparoscopic Adjustable Gastric Binding	LSI	13.44	1.41	25.46
17	Laparoscopic Vertical Banded Gastroplasty vs LSI (8, 9)	Laparoscopic Vertical Banded Gastroplasty	LSI	19.44	7.41	31.46
18	One-Anastomosis Gastric Bypass vs LSI (10, 9)	One-Anastomosis Gastric Bypass	LSI	22.50	10.42	34.58
19	Sleeve Gastrectomy vs LSI (12, 9)	Sleeve Gastrectomy	LSI	19.36	7.52	31.20
	**Result estimates** (source IDs)					
	Biliopancreatic Diversion (1, 15)	Biliopancreatic Diversion	LSI	25.42	16.84	34.00
	Laparoscopic Adjustable Gastric Binding (2, 16)	Laparoscopic Adjustable Gastric Binding	LSI	7.42	5.23	9.61
	Liraglutide 3.0 mg (3)	Liraglutide 3.0 mg	LSI	4.20	3.62	4.78
	One-Anastomosis Gastric Bypass (18)	One-Anastomosis Gastric Bypass	LSI	22.50	10.42	34.58
	Sleeve Gastrectomy (19)	Sleeve Gastrectomy	LSI	19.36	7.52	31.20
	Orlistat (5)	Orlistat	LSI	3.00	2.59	3.41
	Rou-en-Y Gastric Bypass (9, 13, 14)	Rou-en-Y Gastric Bypass	LSI	17.34	13.78	20.90
	Laparoscopic Vertical Banded Gastroplasty (17)	Laparoscopic Vertical Banded Gastroplasty	LSI	19.44	7.41	31.46
	Semaglutide 2.4 mg (11)	Semaglutide 2.4 mg	LSI	8.72	6.97	10.46

Naltr./Bupr.: Naltrexone/Bupropion; **Phen./Topir.**: Phentermine/Topiramate; **POSE**: Primary Obesity Surgery Endoluminal; **IGB**: Intra-Gastric Balloon; **AT**: Aspiration Therapy; **ESG**: Endoscopic Sleeve Gastroplasty; **LAGB**: Laparoscopic Adjustable Gastric Banding; **GCP**: Greater Curvature Plication

Gastric; **LVGB**: Laparoscopic Vertical Banded Gastroplasty; **SG**: Sleeve Gastrectomy; **OAGB**: One-anastomosis gastric bypass; **RYGB**: Roux-en-Y Gastric By-Pass; **SADI**: Single Anastomosis Duodenal Switch; **BPD**: Bilio-Pancreatic Diversion. **LSI:** Lifestyle/Placebo/No therapy. **WMD**: Weighted Mean Difference; **LCI** and **UCI**: Lower and Upper Confidence Intervals.

Table 9S – Direct and estimates of effects of different anti-obesity strategies on 105-156 weeks TBWL%.

ID	Comparison	Active	Control	WMD	LCI 95%	UCI 95%
	Direct estimates					
1	BPD vs LSI	Biliopancreatic Diversion	LSI	25,90	20,41	31,39
2	LAGB vs LSI	Laparoscopic Adjustable Gastric Binding	LSI	7,60	1,63	13,57
3	OAGB vs SG	One-Anastomosis Gastric Bypass	SG	5,12	-4,30	14,55
4	Orlistat vs LSI	Orlistat	LSI	3,00	2,59	3,41
5	RYGB vs BPD	Rou-en-Y Gastric Bypass	BPD	-10,79	-19,13	-2,45
6	RYGB vs LAGB	Rou-en-Y Gastric Bypass	LAGB	11,74	8,77	14,72
7	RYGB vs LVBG	Rou-en-Y Gastric Bypass	LVBG	5,10	1,64	8,56
8	RYGB vs LSI	Rou-en-Y Gastric Bypass	LSI	15,35	10,06	20,64
9	RYGB vs OAGB	Rou-en-Y Gastric Bypass	OAGB	1,70	-0,50	3,90
10	SG vs GCP	Sleeve Gastrectomy	GCP	3,20	-3,46	9,86
11	SG vs LSI	Sleeve Gastrectomy	LSI	13,50	9,97	17,03
12	RYGB vs SG	Rou-en-Y Gastric Bypass	SG	5,70	-1,20	12,59
13	RYGB vs GCP	Rou-en-Y Gastric Bypass	GCP	12,50	6,92	18,08
	**Indirect estimates** (source IDs)					
14	One-Anastomosis Gastric Bypass vs LSI (3, 11)	One-Anastomosis Gastric Bypass	LSI	18,62	8,56	28,69
15	Rou-en-Y Gastric Bypass vs LSI (5, 1)	Rou-en-Y Gastric Bypass	LSI	15,11	5,13	25,09
16	Rou-en-Y Gastric Bypass vs LSI (6, 2)	Rou-en-Y Gastric Bypass	LSI	19,34	12,67	26,01
17	Rou-en-Y Gastric Bypass vs LSI (12, 11)	Rou-en-Y Gastric Bypass	LSI	19,20	11,45	26,94
18	Biliopancreatic Diversion vs LSI (5, 8)	Biliopancreatic Diversion	LSI	26,14	16,26	36,01
19	Laparoscopic Adjustable Gastric Binding vs LSI (6, 8)	Laparoscopic Adjustable Gastric Binding	LSI	3,61	-2,46	9,67
20	Laparoscopic Vertical Banded Gastroplasty vs LSI (7, 8)	Laparoscopic Vertical Banded Gastroplasty	LSI	10,25	3,93	16,57
21	Greater Curvature Plication vs LSI (10, 11)	Greater Curvature Plication	LSI	10,30	2,76	17,84
22	One-Anastomosis Gastric Bypass vs LSI (9, 8)	One-Anastomosis Gastric Bypass	LSI	13,65	7,92	19,38
23	Sleeve Gastrectomy vs LSI (12, 8)	Sleeve Gastrectomy	LSI	9,65	0,96	18,34
24	Greater Curvature Plication vs LSI (13, 8)	Greater Curvature Plication	LSI	2,85	-4,84	10,54
	**Result estimates** (source IDs)					
	Biliopancreatic Diversion (1, 18)	Biliopancreatic Diversion	LSI	25,96	21,16	30,76
	Laparoscopic Adjustable	Laparoscopic Adjustable Gastric Binding	LSI	5,64	1,38	9,89

	Gastric Binding (2, 19)					
	One-Anastomosis Gastric Bypass (14, 22)	One-Anastomosis Gastric Bypass	LSI	14,87	9,89	19,84
	Sleeve Gastrectomy (11, 23)	Sleeve Gastrectomy	LSI	12,95	9,68	16,23
	Orlistat (4)	Orlistat	LSI	3,00	2,59	3,41
	Rou-en-Y Gastric Bypass (8, 15, 16, 17)	Rou-en-Y Gastric Bypass	LSI	17,13	13,70	20,57
	Laparoscopic Vertical Banded Gastroplasty (20)	Laparoscopic Vertical Banded Gastroplasty	LSI	10,25	3,93	16,57
	Greater Curvature Plication (21, 24)	Greater Curvature Plication	LSI	6,65	-0,65	13,95

Naltr./Bupr.: Naltrexone/Bupropion; **Phen./Topir.**: Phentermine/Topiramate; **POSE**: Primary Obesity Surgery Endoluminal; **IGB**: Intra-Gastric Balloon; **AT**: Aspiration Therapy; **ESG**: Endoscopic Sleeve Gastroplasty; **LAGB**: Laparoscopic Adjustable Gastric Banding; **GCP**: Greater Curvature Plication Gastric; **LVGB**: Laparoscopic Vertical Banded Gastroplasty; **SG**: Sleeve Gastrectomy; **OAGB**: One-anastomosis gastric bypass; **RYGB**: Roux-en-Y Gastric By-Pass; **SADI**: Single Anastomosis Duodenal Switch; **BPD**: Bilio-Pancreatic Diversion. **LSI:** Lifestyle/Placebo/No therapy. **WMD**: Weighted Mean Difference; **LCI** and **UCI**: Lower and Upper Confidence Intervals.

Table 9S – TBWL% of each anti-obesity strategy for diabetes status.

TBWL%	**26-52 weeks** *Diabetes*		**53-104 weeks** *Diabetes*		**105-156 weeks** *Diabetes*		**>156 weeks** *Diabetes*	
	Yes	*No*	*Yes*	*No*	*Yes*	*No*	*Yes*	*No*
**OMM**								
Orlistat	2.6	3.1	3.14	3.3	-	3.00	-	-
NB	3.2	5.0	-	-	-	-	-	-
Liraglutide	4.0	4.6	-	5.1	-	4.2	-	-
Semaglutide	6.4	10.0	6.2	11.0	-	8.7	-	-
Tirzepatide	10.5	17.4	10.5	16.9	-	-	-	-
**EP**								
IG-Balloon	-	4.2	-	-	-	-	-	-
POSE	-	3.8	-	-	-	-	-	-
Aspiration	-	8.6	-	-	-	-	-	-
Endo SG	-	12.8	-	-	-	-	-	-
**MBS**								
LAGB	6.0	-	7.0	-	7.0	18.5	5.4	-
SG	12.4	12.8	11.7	-	10.7	26.0	13.2	-
OAGB	15.7	14.7	14.4	-	17.8	31.5	16.3	-
BPD	17.3	29.6	21.2	-	21.9	39.3	22.4	-
RYGB	18.1	14.6	17.3	-	17.8	28.0	19.4	-
SADI	-	28.1	-	-	-	-	-	-
GCP	-	8.8	-	-	-	-	-	-

Naltr./Bupr.: Naltrexone/Bupropion; **Phen./Topir.**: Phentermine/Topiramate; **POSE**: Primary Obesity Surgery Endoluminal; **IGB**: Intra-Gastric Balloon; **AT**: Aspiration Therapy; **ESG**: Endoscopic Sleeve Gastroplasty; **LAGB**: Laparoscopic Adjustable Gastric Banding; **GCP**: Greater Curvature Plication Gastric; **LVGB**: Laparoscopic Vertical Banded Gastroplasty; **SG**: Sleeve Gastrectomy; **OAGB**: One-anastomosis gastric bypass; **RYGB**: Roux-en-Y Gastric By-Pass; **SADI**: Single Anastomosis Duodenal Switch; **BPD**: Bilio-Pancreatic Diversion.

Table 10S – Sensitivity analysis assessing the overall incidence of SAE at endpoint for each intervention. Point estimates express the proportion of patients experiencing at least one SAE during each type of treatment.

Group	Number Studies	Point estimate	Lower limit	Upper limit
Naltr./Bupr.	4	0.035	0.012	0.099
Aspiration	2	0.045	0.020	0.100
POSE	2	0.047	0.027	0.082
Tirzepatide	5	0.058	0.045	0.074
IGB	9	0.060	0.031	0.114
Orlistat	11	0.066	0.042	0.103
Phen./Topir.	2	0.072	0.035	0.140
Liraglutide	9	0.077	0.053	0.111
GCP	4	0.092	0.032	0.237
SG	19	0.109	0.069	0.168
Semaglutide	11	0.114	0.065	0.191
ESG	1	0.156	0.091	0.255
LAGB	11	0.163	0.091	0.275
RYGB	31	0.206	0.153	0.272
OAGB	9	0.237	0.158	0.340
LVBG	1	0.471	0.339	0.606
SADI	1	0.500	0.328	0.672
BPD	4	0.638	0.355	0.850

Random effects analysis

References

1. Astrup A, Carraro R, Finer N, Harper A, Kunesova M, Lean ME, et al. Safety, tolerability and sustained weight loss over 2 years with the once-daily human GLP-1 analog, liraglutide. Int J Obes (Lond) 2012;36(6):843-54. (In eng). DOI: 10.1038/ijo.2011.158.
2. Gudbergsen H, Overgaard A, Henriksen M, Wæhrens EE, Bliddal H, Christensen R, et al. Liraglutide after diet-induced weight loss for pain and weight control in knee osteoarthritis: a randomized controlled trial. Am J Clin Nutr 2021;113(2):314-323. (In eng). DOI: 10.1093/ajcn/nqaa328.
3. Lundgren JR, Janus C, Jensen SBK, Juhl CR, Olsen LM, Christensen RM, et al. Healthy Weight Loss Maintenance with Exercise, Liraglutide, or Both Combined. N Engl J Med 2021;384(18):1719-1730. (In eng). DOI: 10.1056/NEJMoa2028198.
4. O'Neil PM, Birkenfeld AL, McGowan B, Mosenzon O, Pedersen SD, Wharton S, et al. Efficacy and safety of semaglutide compared with liraglutide and placebo for weight loss in patients with obesity: a randomised, double-blind, placebo and active controlled, dose-ranging, phase 2 trial. Lancet 2018;392(10148):637-649. (In eng). DOI: 10.1016/s0140-6736(18)31773-2.
5. Davies MJ, Bergenstal R, Bode B, Kushner RF, Lewin A, Skjøth TV, et al. Efficacy of Liraglutide for Weight Loss Among Patients With Type 2 Diabetes: The SCALE Diabetes Randomized Clinical Trial. Jama 2015;314(7):687-99. (In eng). DOI: 10.1001/jama.2015.9676.
6. Garvey WT, Birkenfeld AL, Dicker D, Mingrone G, Pedersen SD, Satylganova A, et al. Efficacy and Safety of Liraglutide 3.0 mg in Individuals With Overweight or Obesity and Type 2 Diabetes Treated With Basal Insulin: The SCALE Insulin Randomized Controlled Trial. Diabetes Care 2020;43(5):1085-1093. (In eng). DOI: 10.2337/dc19-1745.
7. Wadden TA, Hollander P, Klein S, Niswender K, Woo V, Hale PM, et al. Weight maintenance and additional weight loss with liraglutide after low-calorie-diet-induced weight loss: the SCALE Maintenance randomized study. Int J Obes (Lond) 2013;37(11):1443-51. (In eng). DOI: 10.1038/ijo.2013.120.
8. Wadden TA, Tronieri JS, Sugimoto D, Lund MT, Auerbach P, Jensen C, et al. Liraglutide 3.0 mg and Intensive Behavioral Therapy (IBT) for Obesity in Primary Care: The SCALE IBT Randomized Controlled Trial. Obesity (Silver Spring) 2020;28(3):529-536. (In eng). DOI: 10.1002/oby.22726.
9. le Roux CW, Astrup A, Fujioka K, Greenway F, Lau DCW, Van Gaal L, et al. 3 years of liraglutide versus placebo for type 2 diabetes risk reduction and weight management in individuals with prediabetes: a randomised, double-blind trial. Lancet 2017;389(10077):1399-1409. (In eng). DOI: 10.1016/s0140-6736(17)30069-7.
10. Apovian CM, Aronne L, Rubino D, Still C, Wyatt H, Burns C, et al. A randomized, phase 3 trial of naltrexone SR/bupropion SR on weight and obesity-related risk factors (COR-II). Obesity (Silver Spring) 2013;21(5):935-43. (In eng). DOI: 10.1002/oby.20309.
11. Greenway FL, Fujioka K, Plodkowski RA, Mudaliar S, Guttadauria M, Erickson J, et al. Effect of naltrexone plus bupropion on weight loss in overweight and obese adults (COR-I): a multicentre, randomised, double-blind, placebo-controlled, phase 3 trial. Lancet 2010;376(9741):595-605. (In eng). DOI: 10.1016/s0140-6736(10)60888-4.
12. Hollander P, Gupta AK, Plodkowski R, Greenway F, Bays H, Burns C, et al. Effects of naltrexone sustained-release/bupropion sustained-release combination therapy on body weight and glycemic parameters in overweight and obese patients with type 2 diabetes. Diabetes Care 2013;36(12):4022-9. (In eng). DOI: 10.2337/dc13-0234.
13. Wadden TA, Foreyt JP, Foster GD, Hill JO, Klein S, O'Neil PM, et al. Weight loss with naltrexone SR/bupropion SR combination therapy as an adjunct to behavior modification: the COR-BMOD trial. Obesity (Silver Spring) 2011;19(1):110-20. (In eng). DOI: 10.1038/oby.2010.147.
14. Nissen SE, Wolski KE, Prcela L, Wadden T, Buse JB, Bakris G, et al. Effect of Naltrexone-Bupropion on Major Adverse Cardiovascular Events in Overweight and Obese Patients With Cardiovascular Risk Factors: A Randomized Clinical Trial. Jama 2016;315(10):990-1004. (In eng). DOI: 10.1001/jama.2016.1558.

15. Bakris G, Calhoun D, Egan B, Hellmann C, Dolker M, Kingma I. Orlistat improves blood pressure control in obese subjects with treated but inadequately controlled hypertension. J Hypertens 2002;20(11):2257-67. (In eng). DOI: 10.1097/00004872-200211000-00026.
16. Berne C. A randomized study of orlistat in combination with a weight management programme in obese patients with Type 2 diabetes treated with metformin. Diabet Med 2005;22(5):612-8. (In eng). DOI: 10.1111/j.1464-5491.2004.01474.x.
17. Davidson MH, Hauptman J, DiGirolamo M, Foreyt JP, Halsted CH, Heber D, et al. Weight control and risk factor reduction in obese subjects treated for 2 years with orlistat: a randomized controlled trial. Jama 1999;281(3):235-42. (In eng). DOI: 10.1001/jama.281.3.235.
18. Derosa G, Cicero AF, D'Angelo A, Fogari E, Maffioli P. Effects of 1-year orlistat treatment compared to placebo on insulin resistance parameters in patients with type 2 diabetes. J Clin Pharm Ther 2012;37(2):187-95. (In eng). DOI: 10.1111/j.1365-2710.2011.01280.x.
19. Derosa G, Mugellini A, Ciccarelli L, Fogari R. Randomized, double-blind, placebo-controlled comparison of the action of orlistat, fluvastatin, or both an anthropometric measurements, blood pressure, and lipid profile in obese patients with hypercholesterolemia prescribed a standardized diet. Clin Ther 2003;25(4):1107-22. (In eng). DOI: 10.1016/s0149-2918(03)80070-x.
20. Finer N, James WP, Kopelman PG, Lean ME, Williams G. One-year treatment of obesity: a randomized, double-blind, placebo-controlled, multicentre study of orlistat, a gastrointestinal lipase inhibitor. Int J Obes Relat Metab Disord 2000;24(3):306-13. (In eng). DOI: 10.1038/sj.ijo.0801128.
21. Hill JO, Hauptman J, Anderson JW, Fujioka K, O'Neil PM, Smith DK, et al. Orlistat, a lipase inhibitor, for weight maintenance after conventional dieting: a 1-y study. Am J Clin Nutr 1999;69(6):1108-16. (In eng). DOI: 10.1093/ajcn/69.6.1108.
22. James WP, Avenell A, Broom J, Whitehead J. A one-year trial to assess the value of orlistat in the management of obesity. Int J Obes Relat Metab Disord 1997;21 Suppl 3:S24-30. (In eng).
23. Karhunen L, Franssila-Kallunki A, Rissanen P, Valve R, Kolehmainen M, Rissanen A, et al. Effect of orlistat treatment on body composition and resting energy expenditure during a two-year weight-reduction programme in obese Finns. Int J Obes Relat Metab Disord 2000;24(12):1567-72. (In eng). DOI: 10.1038/sj.ijo.0801443.
24. Kelley DE, Bray GA, Pi-Sunyer FX, Klein S, Hill J, Miles J, et al. Clinical efficacy of orlistat therapy in overweight and obese patients with insulin-treated type 2 diabetes: A 1-year randomized controlled trial. Diabetes Care 2002;25(6):1033-41. (In eng). DOI: 10.2337/diacare.25.6.1033.
25. Miles JM, Leiter L, Hollander P, Wadden T, Anderson JW, Doyle M, et al. Effect of orlistat in overweight and obese patients with type 2 diabetes treated with metformin. Diabetes Care 2002;25(7):1123-8. (In eng). DOI: 10.2337/diacare.25.7.1123.
26. Poston WS, Reeves RS, Haddock CK, Stormer S, Balasubramanyam A, Satterwhite O, et al. Weight loss in obese Mexican Americans treated for 1-year with orlistat and lifestyle modification. Int J Obes Relat Metab Disord 2003;27(12):1486-93. (In eng). DOI: 10.1038/sj.ijo.0802439.
27. Sjöström L, Rissanen A, Andersen T, Boldrin M, Golay A, Koppeschaar HP, et al. Randomised placebo-controlled trial of orlistat for weight loss and prevention of weight regain in obese patients. European Multicentre Orlistat Study Group. Lancet 1998;352(9123):167-72. (In eng). DOI: 10.1016/s0140-6736(97)11509-4.
28. Svendsen M, Helgeland M, Tonstad S. The long-term influence of orlistat on dietary intake in obese subjects with components of metabolic syndrome. J Hum Nutr Diet 2009;22(1):55-63. (In eng). DOI: 10.1111/j.1365-277X.2008.00920.x.
29. Swinburn BA, Carey D, Hills AP, Hooper M, Marks S, Proietto J, et al. Effect of orlistat on cardiovascular disease risk in obese adults. Diabetes, obesity & metabolism 2005;7(3):254-62. (In eng). DOI: 10.1111/j.1463-1326.2004.00467.x.
30. Zavoral JH. Treatment with orlistat reduces cardiovascular risk in obese patients. J Hypertens 1998;16(12 Pt 2):2013-7. (In eng). DOI: 10.1097/00004872-199816121-00024.
31. Hollander PA, Elbein SC, Hirsch IB, Kelley D, McGill J, Taylor T, et al. Role of orlistat in the treatment of obese patients with type 2 diabetes. A 1-year randomized double-blind study. Diabetes Care 1998;21(8):1288-94. (In eng). DOI: 10.2337/diacare.21.8.1288.
32. Krempf M, Louvet JP, Allanic H, Miloradovich T, Joubert JM, Attali JR. Weight reduction and long-term maintenance after 18 months treatment with orlistat for obesity. Int J Obes Relat Metab Disord 2003;27(5):591-7. (In eng). DOI: 10.1038/sj.ijo.0802281.

33. Hauptman J, Lucas C, Boldrin MN, Collins H, Segal KR. Orlistat in the long-term treatment of obesity in primary care settings. Arch Fam Med 2000;9(2):160-7. (In eng). DOI: 10.1001/archfami.9.2.160.
34. Rössner S, Sjöström L, Noack R, Meinders AE, Noseda G. Weight loss, weight maintenance, and improved cardiovascular risk factors after 2 years treatment with orlistat for obesity. European Orlistat Obesity Study Group. Obes Res 2000;8(1):49-61. (In eng). DOI: 10.1038/oby.2000.8.
35. Richelsen B, Tonstad S, Rössner S, Toubro S, Niskanen L, Madsbad S, et al. Effect of orlistat on weight regain and cardiovascular risk factors following a very-low-energy diet in abdominally obese patients: a 3-year randomized, placebo-controlled study. Diabetes Care 2007;30(1):27-32. (In eng). DOI: 10.2337/dc06-0210.
36. Torgerson JS, Hauptman J, Boldrin MN, Sjöström L. XENical in the prevention of diabetes in obese subjects (XENDOS) study: a randomized study of orlistat as an adjunct to lifestyle changes for the prevention of type 2 diabetes in obese patients. Diabetes Care 2004;27(1):155-61. (In eng). DOI: 10.2337/diacare.27.1.155.
37. Allison DB, Gadde KM, Garvey WT, Peterson CA, Schwiers ML, Najarian T, et al. Controlled-release phentermine/topiramate in severely obese adults: a randomized controlled trial (EQUIP). Obesity (Silver Spring) 2012;20(2):330-42. (In eng). DOI: 10.1038/oby.2011.330.
38. Gadde KM, Allison DB, Ryan DH, Peterson CA, Troupin B, Schwiers ML, et al. Effects of low-dose, controlled-release, phentermine plus topiramate combination on weight and associated comorbidities in overweight and obese adults (CONQUER): a randomised, placebo-controlled, phase 3 trial. Lancet 2011;377(9774):1341-52. (In eng). DOI: 10.1016/s0140-6736(11)60205-5.
39. Kosiborod MN, Petrie MC, Borlaug BA, Butler J, Davies MJ, Hovingh GK, et al. Semaglutide in Patients with Obesity-Related Heart Failure and Type 2 Diabetes. N Engl J Med 2024;390(15):1394-1407. (In eng). DOI: 10.1056/NEJMoa2313917.
40. Kosiborod MN, Abildstrøm SZ, Borlaug BA, Butler J, Rasmussen S, Davies M, et al. Semaglutide in Patients with Heart Failure with Preserved Ejection Fraction and Obesity. N Engl J Med 2023;389(12):1069-1084. (In eng). DOI: 10.1056/NEJMoa2306963.
41. McGowan BM, Bruun JM, Capehorn M, Pedersen SD, Pietiläinen KH, Muniraju HAK, et al. Efficacy and safety of once-weekly semaglutide 2·4 mg versus placebo in people with obesity and prediabetes (STEP 10): a randomised, double-blind, placebo-controlled, multicentre phase 3 trial. Lancet Diabetes Endocrinol 2024;12(9):631-642. (In eng). DOI: 10.1016/s2213-8587(24)00182-7.
42. Bliddal H, Bays H, Czernichow S, Uddén Hemmingsson J, Hjelmesæth J, Hoffmann Morville T, et al. Once-Weekly Semaglutide in Persons with Obesity and Knee Osteoarthritis. N Engl J Med 2024;391(17):1573-1583. (In eng). DOI: 10.1056/NEJMoa2403664.
43. Davies M, Færch L, Jeppesen OK, Pakseresht A, Pedersen SD, Perreault L, et al. Semaglutide 2·4 mg once a week in adults with overweight or obesity, and type 2 diabetes (STEP 2): a randomised, double-blind, double-dummy, placebo-controlled, phase 3 trial. Lancet 2021;397(10278):971-984. (In eng). DOI: 10.1016/s0140-6736(21)00213-0.
44. Kadowaki T, Isendahl J, Khalid U, Lee SY, Nishida T, Ogawa W, et al. Semaglutide once a week in adults with overweight or obesity, with or without type 2 diabetes in an east Asian population (STEP 6): a randomised, double-blind, double-dummy, placebo-controlled, phase 3a trial. Lancet Diabetes Endocrinol 2022;10(3):193-206. (In eng). DOI: 10.1016/s2213-8587(22)00008-0.
45. Rubino D, Abrahamsson N, Davies M, Hesse D, Greenway FL, Jensen C, et al. Effect of Continued Weekly Subcutaneous Semaglutide vs Placebo on Weight Loss Maintenance in Adults With Overweight or Obesity: The STEP 4 Randomized Clinical Trial. Jama 2021;325(14):1414-1425. (In eng). DOI: 10.1001/jama.2021.3224.
46. Wadden TA, Bailey TS, Billings LK, Davies M, Frias JP, Koroleva A, et al. Effect of Subcutaneous Semaglutide vs Placebo as an Adjunct to Intensive Behavioral Therapy on Body Weight in Adults With Overweight or Obesity: The STEP 3 Randomized Clinical Trial. Jama 2021;325(14):1403-1413. (In eng). DOI: 10.1001/jama.2021.1831.
47. Wilding JPH, Batterham RL, Calanna S, Davies M, Van Gaal LF, Lingvay I, et al. Once-Weekly Semaglutide in Adults with Overweight or Obesity. N Engl J Med 2021;384(11):989-1002. (In eng). DOI: 10.1056/NEJMoa2032183.
48. Garvey WT, Batterham RL, Bhatta M, Buscemi S, Christensen LN, Frias JP, et al. Two-year effects of semaglutide in adults with overweight or obesity: the STEP 5 trial. Nat Med 2022;28(10):2083-2091. (In eng). DOI: 10.1038/s41591-022-02026-4.

49. Lincoff AM, Brown-Frandsen K, Colhoun HM, Deanfield J, Emerson SS, Esbjerg S, et al. Semaglutide and Cardiovascular Outcomes in Obesity without Diabetes. N Engl J Med 2023;389(24):2221-2232. (In eng). DOI: 10.1056/NEJMoa2307563.
50. Aronne LJ, Sattar N, Horn DB, Bays HE, Wharton S, Lin WY, et al. Continued Treatment With Tirzepatide for Maintenance of Weight Reduction in Adults With Obesity: The SURMOUNT-4 Randomized Clinical Trial. Jama 2024;331(1):38-48. (In eng). DOI: 10.1001/jama.2023.24945.
51. Loomba R, Hartman ML, Lawitz EJ, Vuppalanchi R, Boursier J, Bugianesi E, et al. Tirzepatide for Metabolic Dysfunction-Associated Steatohepatitis with Liver Fibrosis. N Engl J Med 2024;391(4):299-310. (In eng). DOI: 10.1056/NEJMoa2401943.
52. Malhotra A, Grunstein RR, Fietze I, Weaver TE, Redline S, Azarbarzin A, et al. Tirzepatide for the Treatment of Obstructive Sleep Apnea and Obesity. N Engl J Med 2024 (In eng). DOI: 10.1056/NEJMoa2404881.
53. Garvey WT, Frias JP, Jastreboff AM, le Roux CW, Sattar N, Aizenberg D, et al. Tirzepatide once weekly for the treatment of obesity in people with type 2 diabetes (SURMOUNT-2): a double-blind, randomised, multicentre, placebo-controlled, phase 3 trial. Lancet 2023;402(10402):613-626. (In eng). DOI: 10.1016/s0140-6736(23)01200-x.
54. Jastreboff AM, Aronne LJ, Ahmad NN, Wharton S, Connery L, Alves B, et al. Tirzepatide Once Weekly for the Treatment of Obesity. N Engl J Med 2022;387(3):205-216. (In eng). DOI: 10.1056/NEJMoa2206038.
55. Sullivan S, Stein R, Jonnalagadda S, Mullady D, Edmundowicz S. Aspiration therapy leads to weight loss in obese subjects: a pilot study. Gastroenterology 2013;145(6):1245-52.e1-5. (In eng). DOI: 10.1053/j.gastro.2013.08.056.
56. Thompson CC, Abu Dayyeh BK, Kushner R, Sullivan S, Schorr AB, Amaro A, et al. Percutaneous Gastrostomy Device for the Treatment of Class II and Class III Obesity: Results of a Randomized Controlled Trial. Am J Gastroenterol 2017;112(3):447-457. (In eng). DOI: 10.1038/ajg.2016.500.
57. Glaysher MA, Mohanaruban A, Prechtl CG, Goldstone AP, Miras AD, Lord J, et al. A randomised controlled trial of a duodenal-jejunal bypass sleeve device (EndoBarrier) compared with standard medical therapy for the management of obese subjects with type 2 diabetes mellitus. BMJ open 2017;7(11):e018598. (In eng). DOI: 10.1136/bmjopen-2017-018598.
58. Koehestanie P, de Jonge C, Berends FJ, Janssen IM, Bouvy ND, Greve JW. The effect of the endoscopic duodenal-jejunal bypass liner on obesity and type 2 diabetes mellitus, a multicenter randomized controlled trial. Ann Surg 2014;260(6):984-92. (In eng). DOI: 10.1097/sla.0000000000000794.
59. Caiazzo R, Branche J, Raverdy V, Czernichow S, Carette C, Robert M, et al. Efficacy and Safety of the Duodeno-Jejunal Bypass Liner in Patients With Metabolic Syndrome: A Multicenter Randomized Controlled Trial (ENDOMETAB). Ann Surg 2020;272(5):696-702. (In eng). DOI: 10.1097/sla.0000000000004339.
60. Ruban A, Miras AD, Glaysher MA, Goldstone AP, Prechtl CG, Johnson N, et al. Duodenal-Jejunal Bypass Liner for the management of Type 2 Diabetes Mellitus and Obesity: A Multicenter Randomized Controlled Trial. Ann Surg 2022;275(3):440-447. (In eng). DOI: 10.1097/sla.0000000000004980.
61. Rodriguez L, Reyes E, Fagalde P, Oltra MS, Saba J, Aylwin CG, et al. Pilot clinical study of an endoscopic, removable duodenal-jejunal bypass liner for the treatment of type 2 diabetes. Diabetes Technol Ther 2009;11(11):725-32. (In eng). DOI: 10.1089/dia.2009.0063.
62. Petry TZ, Fabbrini E, Otoch JP, Carmona MA, Caravatto PP, Salles JE, et al. Effect of Duodenal-Jejunal Bypass Surgery on Glycemic Control in Type 2 Diabetes: A Randomized Controlled Trial. Obesity (Silver Spring) 2015;23(10):1973-9. (In eng). DOI: 10.1002/oby.21190.
63. Abu Dayyeh BK, Bazerbachi F, Vargas EJ, Sharaiha RZ, Thompson CC, Thaemert BC, et al. Endoscopic sleeve gastroplasty for treatment of class 1 and 2 obesity (MERIT): a prospective, multicentre, randomised trial. Lancet 2022;400(10350):441-451. (In eng). DOI: 10.1016/s0140-6736(22)01280-6.
64. Coffin B, Maunoury V, Pattou F, Hébuterne X, Schneider S, Coupaye M, et al. Impact of Intragastric Balloon Before Laparoscopic Gastric Bypass on Patients with Super Obesity: a Randomized Multicenter Study. Obes Surg 2017;27(4):902-909. (In eng). DOI: 10.1007/s11695-016-2383-x.

65. Genco A, Maselli R, Frangella F, Cipriano M, Paone E, Meuti V, et al. Effect of consecutive intragastric balloon (BIB®) plus diet versus single BIB® plus diet on eating disorders not otherwise specified (EDNOS) in obese patients. Obes Surg 2013;23(12):2075-9. (In eng). DOI: 10.1007/s11695-013-1028-6.
66. Dargent J, Mion F, Costil V, Ecochard R, Pontette F, Mion V, et al. Multicenter Randomized Study of Obesity Treatment with Minimally Invasive Injection of Hyaluronic Acid Versus and Combined with Intragastric Balloon. Obes Surg 2015;25(10):1842-7. (In eng). DOI: 10.1007/s11695-015-1648-0.
67. Courcoulas A, Abu Dayyeh BK, Eaton L, Robinson J, Woodman G, Fusco M, et al. Intragastric balloon as an adjunct to lifestyle intervention: a randomized controlled trial. Int J Obes (Lond) 2017;41(3):427-433. (In eng). DOI: 10.1038/ijo.2016.229.
68. Fuller NR, Pearson S, Lau NS, Wlodarczyk J, Halstead MB, Tee HP, et al. An intragastric balloon in the treatment of obese individuals with metabolic syndrome: a randomized controlled study. Obesity (Silver Spring) 2013;21(8):1561-70. (In eng). DOI: 10.1002/oby.20414.
69. Ponce J, Quebbemann BB, Patterson EJ. Prospective, randomized, multicenter study evaluating safety and efficacy of intragastric dual-balloon in obesity. Surg Obes Relat Dis 2013;9(2):290-5. (In eng). DOI: 10.1016/j.soard.2012.07.007.
70. Abu Dayyeh BK, Maselli DB, Rapaka B, Lavin T, Noar M, Hussan H, et al. Adjustable intragastric balloon for treatment of obesity: a multicentre, open-label, randomised clinical trial. Lancet 2021;398(10315):1965-1973. (In eng). DOI: 10.1016/s0140-6736(21)02394-1.
71. Lee YM, Low HC, Lim LG, Dan YY, Aung MO, Cheng CL, et al. Intragastric balloon significantly improves nonalcoholic fatty liver disease activity score in obese patients with nonalcoholic steatohepatitis: a pilot study. Gastrointest Endosc 2012;76(4):756-60. (In eng). DOI: 10.1016/j.gie.2012.05.023.
72. Sullivan S, Swain J, Woodman G, Edmundowicz S, Hassanein T, Shayani V, et al. Randomized sham-controlled trial of the 6-month swallowable gas-filled intragastric balloon system for weight loss. Surg Obes Relat Dis 2018;14(12):1876-1889. (In eng). DOI: 10.1016/j.soard.2018.09.486.
73. Miller K, Turró R, Greve JW, Bakker CM, Buchwald JN, Espinós JC. MILEPOST Multicenter Randomized Controlled Trial: 12-Month Weight Loss and Satiety Outcomes After pose (SM) vs. Medical Therapy. Obes Surg 2017;27(2):310-322. (In eng). DOI: 10.1007/s11695-016-2295-9.
74. Sullivan S, Swain JM, Woodman G, Antonetti M, De La Cruz-Muñoz N, Jonnalagadda SS, et al. Randomized sham-controlled trial evaluating efficacy and safety of endoscopic gastric plication for primary obesity: The ESSENTIAL trial. Obesity (Silver Spring) 2017;25(2):294-301. (In eng). DOI: 10.1002/oby.21702.
75. Feigel-Guiller B, Drui D, Dimet J, Zair Y, Le Bras M, Fuertes-Zamorano N, et al. Laparoscopic Gastric Banding in Obese Patients with Sleep Apnea: A 3-Year Controlled Study and Follow-up After 10 Years. Obes Surg 2015;25(10):1886-92. (In eng). DOI: 10.1007/s11695-015-1627-5.
76. Simonson DC, Vernon A, Foster K, Halperin F, Patti ME, Goldfine AB. Adjustable gastric band surgery or medical management in patients with type 2 diabetes and obesity: three-year results of a randomized trial. Surg Obes Relat Dis 2019;15(12):2052-2059. (In eng). DOI: 10.1016/j.soard.2019.03.038.
77. O'Brien PE, Brennan L, Laurie C, Brown W. Intensive medical weight loss or laparoscopic adjustable gastric banding in the treatment of mild to moderate obesity: long-term follow-up of a prospective randomised trial. Obes Surg 2013;23(9):1345-53. (In eng). DOI: 10.1007/s11695-013-0990-3.
78. Morino M, Toppino M, Bonnet G, del Genio G. Laparoscopic adjustable silicone gastric banding versus vertical banded gastroplasty in morbidly obese patients: a prospective randomized controlled clinical trial. Ann Surg 2003;238(6):835-41; discussion 841-2. (In eng). DOI: 10.1097/01.sla.0000098627.18574.72.
79. Dowsey MM, Brown WA, Cochrane A, Burton PR, Liew D, Choong PF. Effect of Bariatric Surgery on Risk of Complications After Total Knee Arthroplasty: A Randomized Clinical Trial. JAMA Netw Open 2022;5(4):e226722. (In eng). DOI: 10.1001/jamanetworkopen.2022.6722.
80. Dixon JB, O'Brien PE, Playfair J, Chapman L, Schachter LM, Skinner S, et al. Adjustable gastric banding and conventional therapy for type 2 diabetes: a randomized controlled trial. Jama 2008;299(3):316-23. (In eng). DOI: 10.1001/jama.299.3.316.

81. Dixon JB, Schachter LM, O'Brien PE, Jones K, Grima M, Lambert G, et al. Surgical vs conventional therapy for weight loss treatment of obstructive sleep apnea: a randomized controlled trial. Jama 2012;308(11):1142-9. (In eng). DOI: 10.1001/2012.jama.11580.
82. Xiang AH, Trigo E, Martinez M, Katkhouda N, Beale E, Wang X, et al. Impact of Gastric Banding Versus Metformin on β-Cell Function in Adults With Impaired Glucose Tolerance or Mild Type 2 Diabetes. Diabetes Care 2018;41(12):2544-2551. (In eng). DOI: 10.2337/dc18-1662.
83. Nilsell K, Thörne A, Sjöstedt S, Apelman J, Pettersson N. Prospective randomised comparison of adjustable gastric banding and vertical banded gastroplasty for morbid obesity. Eur J Surg 2001;167(7):504-9. (In eng). DOI: 10.1080/110241501316914876.
84. Scozzari G, Farinella E, Bonnet G, Toppino M, Morino M. Laparoscopic adjustable silicone gastric banding vs laparoscopic vertical banded gastroplasty in morbidly obese patients: long-term results of a prospective randomized controlled clinical trial. Obes Surg 2009;19(8):1108-15. (In eng). DOI: 10.1007/s11695-009-9871-1.
85. Lundell L, Ruth M, Olbe L. Vertical banded gastroplasty or gastric banding for morbid obesity: effects on gastro-oesophageal reflux. Eur J Surg 1997;163(7):525-31. (In eng).
86. Darabi S, Talebpour M, Zeinoddini A, Heidari R. Laparoscopic gastric plication versus mini-gastric bypass surgery in the treatment of morbid obesity: a randomized clinical trial. Surg Obes Relat Dis 2013;9(6):914-9. (In eng). DOI: 10.1016/j.soard.2013.07.012.
87. Musella M, Vitiello A, Berardi G, Velotti N, Pesce M, Sarnelli G. Evaluation of reflux following sleeve gastrectomy and one anastomosis gastric bypass: 1-year results from a randomized open-label controlled trial. Surg Endosc 2021;35(12):6777-6785. (In eng). DOI: 10.1007/s00464-020-08182-3.
88. Roushdy A, Abdel-Razik MA, Emile SH, Farid M, Elbanna HG, Khafagy W, et al. Fasting Ghrelin and Postprandial GLP-1 Levels in Patients With Morbid Obesity and Medical Comorbidities After Sleeve Gastrectomy and One-anastomosis Gastric Bypass: A Randomized Clinical Trial. Surg Laparosc Endosc Percutan Tech 2020;31(1):28-35. (In eng). DOI: 10.1097/sle.0000000000000844.
89. Hany M, Zidan A, Aboelsoud MR, Torensma B. Laparoscopic sleeve gastrectomy vs one-anastomosis gastric bypass 5-year follow-up: a single-blinded randomized controlled trial. J Gastrointest Surg 2024;28(5):621-633. (In eng). DOI: 10.1016/j.gassur.2024.01.038.
90. Jain M, Tantia O, Goyal G, Chaudhuri T, Khanna S, Poddar A, et al. LSG vs MGB-OAGB: 5-Year Follow-up Data and Comparative Outcome of the Two Procedures over Long Term-Results of a Randomised Control Trial. Obes Surg 2021;31(3):1223-1232. (In eng). DOI: 10.1007/s11695-020-05119-6.
91. Lee WJ, Chong K, Lin YH, Wei JH, Chen SC. Laparoscopic sleeve gastrectomy versus single anastomosis (mini-) gastric bypass for the treatment of type 2 diabetes mellitus: 5-year results of a randomized trial and study of incretin effect. Obes Surg 2014;24(9):1552-62. (In eng). DOI: 10.1007/s11695-014-1344-5.
92. Hedberg S, Thorell A, Österberg J, Peltonen M, Andersson E, Näslund E, et al. Comparison of Sleeve Gastrectomy vs Roux-en-Y Gastric Bypass: A Randomized Clinical Trial. JAMA Netw Open 2024;7(1):e2353141. (In eng). DOI: 10.1001/jamanetworkopen.2023.53141.
93. Skroubis G, Kouri N, Mead N, Kalfarentzos F. Long-term results of a prospective comparison of Roux-en-Y gastric bypass versus a variant of biliopancreatic diversion in a non-superobese population (BMI 35-50 kg/m(2)). Obes Surg 2014;24(2):197-204. (In eng). DOI: 10.1007/s11695-013-1081-1.
94. Mingrone G, Panunzi S, De Gaetano A, Guidone C, Iaconelli A, Capristo E, et al. Metabolic surgery versus conventional medical therapy in patients with type 2 diabetes: 10-year follow-up of an open-label, single-centre, randomised controlled trial. Lancet 2021;397(10271):293-304. (In eng). DOI: 10.1016/s0140-6736(20)32649-0.
95. Salte OBK, Olbers T, Risstad H, Fagerland MW, Søvik TT, Blom-Høgestøl IK, et al. Ten-Year Outcomes Following Roux-en-Y Gastric Bypass vs Duodenal Switch for High Body Mass Index: A Randomized Clinical Trial. JAMA Netw Open 2024;7(6):e2414340. (In eng). DOI: 10.1001/jamanetworkopen.2024.14340.
96. Hall JC, Watts JM, O'Brien PE, Dunstan RE, Walsh JF, Slavotinek AH, et al. Gastric surgery for morbid obesity. The Adelaide Study. Ann Surg 1990;211(4):419-27. (In eng). DOI: 10.1097/00000658-199004000-00007.

97. Casajoana A, Guerrero-Pérez F, García Ruiz de Gordejuela A, Admella V, Sorribas M, Vidal-Alabró A, et al. Role of Gastrointestinal Hormones as a Predictive Factor for Long-Term Diabetes Remission: Randomized Trial Comparing Metabolic Gastric Bypass, Sleeve Gastrectomy, and Greater Curvature Plication. Obes Surg 2021;31(4):1733-1744. (In eng). DOI: 10.1007/s11695-020-05192-x.
98. Nguyen NT, Kim E, Vu S, Phelan M. Ten-year Outcomes of a Prospective Randomized Trial of Laparoscopic Gastric Bypass Versus Laparoscopic Gastric Banding. Ann Surg 2018;268(1):106-113. (In eng). DOI: 10.1097/sla.0000000000002348.
99. Courcoulas AP, Gallagher JW, Neiberg RH, Eagleton EB, DeLany JP, Lang W, et al. Bariatric Surgery vs Lifestyle Intervention for Diabetes Treatment: 5-Year Outcomes From a Randomized Trial. J Clin Endocrinol Metab 2020;105(3):866-76. (In eng). DOI: 10.1210/clinem/dgaa006.
100. Cohen RV, Pereira TV, Aboud CM, Petry TBZ, Lopes Correa JL, Schiavon CA, et al. Effect of Gastric Bypass vs Best Medical Treatment on Early-Stage Chronic Kidney Disease in Patients With Type 2 Diabetes and Obesity: A Randomized Clinical Trial. JAMA Surg 2020;155(8):e200420. (In eng). DOI: 10.1001/jamasurg.2020.0420.
101. Cheng A, Yeoh E, Moh A, Low S, Tan CH, Lam B, et al. Roux-en-Y gastric bypass versus best medical treatment for type 2 diabetes mellitus in adults with body mass index between 27 and 32 kg/m(2): A 5-year randomized controlled trial. Diabetes research and clinical practice 2022;188:109900. (In eng). DOI: 10.1016/j.diabres.2022.109900.
102. Schauer PR, Bhatt DL, Kirwan JP, Wolski K, Aminian A, Brethauer SA, et al. Bariatric Surgery versus Intensive Medical Therapy for Diabetes - 5-Year Outcomes. N Engl J Med 2017;376(7):641-651. (In eng). DOI: 10.1056/NEJMoa1600869.
103. Lee WJ, Huang MT, Yu PJ, Wang W, Chen TC. Laparoscopic vertical banded gastroplasty and laparoscopic gastric bypass: a comparison. Obes Surg 2004;14(5):626-34. (In eng). DOI: 10.1381/096089204323093390.
104. Olbers T, Fagevik-Olsén M, Maleckas A, Lönroth H. Randomized clinical trial of laparoscopic Roux-en-Y gastric bypass versus laparoscopic vertical banded gastroplasty for obesity. Br J Surg 2005;92(5):557-62. (In eng). DOI: 10.1002/bjs.4974.
105. Werling M, Fändriks L, Björklund P, Maleckas A, Brandberg J, Lönroth H, et al. Long-term results of a randomized clinical trial comparing Roux-en-Y gastric bypass with vertical banded gastroplasty. Br J Surg 2013;100(2):222-30. (In eng). DOI: 10.1002/bjs.8975.
106. MacLean LD, Rhode BM, Sampalis J, Forse RA. Results of the surgical treatment of obesity. Am J Surg 1993;165(1):155-60; discussion 160-2. (In eng). DOI: 10.1016/s0002-9610(05)80420-9.
107. Cummings DE, Arterburn DE, Westbrook EO, Kuzma JN, Stewart SD, Chan CP, et al. Gastric bypass surgery vs intensive lifestyle and medical intervention for type 2 diabetes: the CROSSROADS randomised controlled trial. Diabetologia 2016;59(5):945-53. (In eng). DOI: 10.1007/s00125-016-3903-x.
108. Liang Z, Wu Q, Chen B, Yu P, Zhao H, Ouyang X. Effect of laparoscopic Roux-en-Y gastric bypass surgery on type 2 diabetes mellitus with hypertension: a randomized controlled trial. Diabetes research and clinical practice 2013;101(1):50-6. (In eng). DOI: 10.1016/j.diabres.2013.04.005.
109. Schiavon CA, Bhatt DL, Ikeoka D, Santucci EV, Santos RN, Damiani LP, et al. Three-Year Outcomes of Bariatric Surgery in Patients With Obesity and Hypertension : A Randomized Clinical Trial. Ann Intern Med 2020;173(9):685-693. (In eng). DOI: 10.7326/m19-3781.
110. Ikramuddin S, Korner J, Lee WJ, Thomas AJ, Connett JE, Bantle JP, et al. Lifestyle Intervention and Medical Management With vs Without Roux-en-Y Gastric Bypass and Control of Hemoglobin A1c, LDL Cholesterol, and Systolic Blood Pressure at 5 Years in the Diabetes Surgery Study. Jama 2018;319(3):266-278. (In eng). DOI: 10.1001/jama.2017.20813.
111. Delko T, Kraljević M, Lazaridis, II, Köstler T, Jomard A, Taheri A, et al. Laparoscopic Roux-Y-gastric bypass versus laparoscopic one-anastomosis gastric bypass for obesity: clinical & metabolic results of a prospective randomized controlled trial. Surg Endosc 2024;38(7):3875-3886. (In eng). DOI: 10.1007/s00464-024-10907-7.
112. Eskandaros MS, Abbass A, Zaid MH, Darwish AA. Laparoscopic One Anastomosis Gastric Bypass Versus Laparoscopic Roux-en-Y Gastric Bypass Effects on Pre-existing Mild-to-Moderate Gastroesophageal Reflux Disease in Patients with Obesity: a Randomized Controlled Study. Obes Surg 2021;31(11):4673-4681. (In eng). DOI: 10.1007/s11695-021-05667-5.

113. Karagul S, Senol S, Karakose O, Uzunoglu K, Kayaalp C. One Anastomosis Gastric Bypass versus Roux-en-Y Gastric Bypass: A Randomized Prospective Trial. Medicina (Kaunas) 2024;60(2) (In eng). DOI: 10.3390/medicina60020256.
114. Singh B, Saikaustubh Y, Singla V, Kumar A, Ahuja V, Gupta Y, et al. One Anastomosis Gastric Bypass (OAGB) vs Roux en Y Gastric Bypass (RYGB) for Remission of T2DM in Patients with Morbid Obesity: a Randomized Controlled Trial. Obes Surg 2023;33(4):1218-1227. (In eng). DOI: 10.1007/s11695-023-06515-4.
115. Level L, Rojas A, Piñango S, Avariano Y. One anastomosis gastric bypass vs. Roux-en-Y gastric bypass: a 5-year follow-up prospective randomized trial. Langenbecks Arch Surg 2021;406(1):171-179. (In eng). DOI: 10.1007/s00423-020-01949-1.
116. Robert M, Espalieu P, Pelascini E, Caiazzo R, Sterkers A, Khamphommala L, et al. Efficacy and safety of one anastomosis gastric bypass versus Roux-en-Y gastric bypass for obesity (YOMEGA): a multicentre, randomised, open-label, non-inferiority trial. Lancet 2019;393(10178):1299-1309. (In eng). DOI: 10.1016/s0140-6736(19)30475-1.
117. Biter LU, van Buuren MMA, Mannaerts GHH, Apers JA, Dunkelgrün M, Vijgen G. Quality of Life 1 Year After Laparoscopic Sleeve Gastrectomy Versus Laparoscopic Roux-en-Y Gastric Bypass: a Randomized Controlled Trial Focusing on Gastroesophageal Reflux Disease. Obes Surg 2017;27(10):2557-2565. (In eng). DOI: 10.1007/s11695-017-2688-4.
118. Karamanakos SN, Vagenas K, Kalfarentzos F, Alexandrides TK. Weight loss, appetite suppression, and changes in fasting and postprandial ghrelin and peptide-YY levels after Roux-en-Y gastric bypass and sleeve gastrectomy: a prospective, double blind study. Ann Surg 2008;247(3):401-7. (In eng). DOI: 10.1097/SLA.0b013e318156f012.
119. Keidar A, Hershkop KJ, Marko L, Schweiger C, Hecht L, Bartov N, et al. Roux-en-Y gastric bypass vs sleeve gastrectomy for obese patients with type 2 diabetes: a randomised trial. Diabetologia 2013;56(9):1914-8. (In eng). DOI: 10.1007/s00125-013-2965-2.
120. Paluszkiewicz R, Kalinowski P, Wróblewski T, Bartoszewicz Z, Białobrzeska-Paluszkiewicz J, Ziarkiewicz-Wróblewska B, et al. Prospective randomized clinical trial of laparoscopic sleeve gastrectomy versus open Roux-en-Y gastric bypass for the management of patients with morbid obesity. Wideochir Inne Tech Maloinwazyjne 2012;7(4):225-32. (In eng). DOI: 10.5114/wiitm.2012.32384.
121. Ramón JM, Salvans S, Crous X, Puig S, Goday A, Benaiges D, et al. Effect of Roux-en-Y gastric bypass vs sleeve gastrectomy on glucose and gut hormones: a prospective randomised trial. J Gastrointest Surg 2012;16(6):1116-22. (In eng). DOI: 10.1007/s11605-012-1855-0.
122. Verrastro O, Panunzi S, Castagneto-Gissey L, De Gaetano A, Lembo E, Capristo E, et al. Bariatric-metabolic surgery versus lifestyle intervention plus best medical care in non-alcoholic steatohepatitis (BRAVES): a multicentre, open-label, randomised trial. Lancet 2023;401(10390):1786-1797. (In eng). DOI: 10.1016/s0140-6736(23)00634-7.
123. Tang Q, Sun Z, Zhang N, Xu G, Song P, Xu L, et al. Cost-Effectiveness of Bariatric Surgery for Type 2 Diabetes Mellitus: A Randomized Controlled Trial in China. Medicine (Baltimore) 2016;95(20):e3522. (In eng). DOI: 10.1097/md.0000000000003522.
124. Wallenius V, Alaraj A, Björnfot N, Orrenius B, Kylebäck A, Björklund P, et al. Sleeve gastrectomy and Roux-en-Y gastric bypass in the treatment of type 2 diabetes. Two-year results from a Swedish multicenter randomized controlled trial. Surg Obes Relat Dis 2020;16(8):1035-1044. (In eng). DOI: 10.1016/j.soard.2020.04.033.
125. Pajecki D, Dos Anjos Pinheiro MC, Dantas ACB, Corsi GC, Dias MCG, Santo MA. Sleeve Gastrectomy Versus Roux-en-Y Gastric Bypass for Treating Obesity in Patients > 65 Years Old: 3-Year Outcomes of a Randomized Trial. J Gastrointest Surg 2023;27(4):780-782. (In eng). DOI: 10.1007/s11605-023-05608-w.
126. Catheline JM, Fysekidis M, Bendacha Y, Portal JJ, Huten N, Chouillard E, et al. Prospective, multicentric, comparative study between sleeve gastrectomy and Roux-en-Y gastric bypass, 277 patients, 3 years follow-up. J Visc Surg 2019;156(6):497-506. (In eng). DOI: 10.1016/j.jviscsurg.2019.04.013.
127. Kehagias I, Karamanakos SN, Argentou M, Kalfarentzos F. Randomized clinical trial of laparoscopic Roux-en-Y gastric bypass versus laparoscopic sleeve gastrectomy for the management of patients with BMI < 50 kg/m2. Obes Surg 2011;21(11):1650-6. (In eng). DOI: 10.1007/s11695-011-0479-x.

128. Svanevik M, Lorentzen J, Borgeraas H, Sandbu R, Seip B, Medhus AW, et al. Patient-reported outcomes, weight loss, and remission of type 2 diabetes 3 years after gastric bypass and sleeve gastrectomy (Oseberg); a single-centre, randomised controlled trial. Lancet Diabetes Endocrinol 2023;11(8):555-566. (In eng). DOI: 10.1016/s2213-8587(23)00127-4.
129. Yang J, Wang C, Cao G, Yang W, Yu S, Zhai H, et al. Long-term effects of laparoscopic sleeve gastrectomy versus roux-en-Y gastric bypass for the treatment of Chinese type 2 diabetes mellitus patients with body mass index 28-35 kg/m(2). BMC Surg 2015;15:88. (In eng). DOI: 10.1186/s12893-015-0074-5.
130. Ignat M, Vix M, Imad I, D'Urso A, Perretta S, Marescaux J, et al. Randomized trial of Roux-en-Y gastric bypass versus sleeve gastrectomy in achieving excess weight loss. Br J Surg 2017;104(3):248-256. (In eng). DOI: 10.1002/bjs.10400.
131. Peterli R, Wölnerhanssen BK, Peters T, Vetter D, Kröll D, Borbély Y, et al. Effect of Laparoscopic Sleeve Gastrectomy vs Laparoscopic Roux-en-Y Gastric Bypass on Weight Loss in Patients With Morbid Obesity: The SM-BOSS Randomized Clinical Trial. Jama 2018;319(3):255-265. (In eng). DOI: 10.1001/jama.2017.20897.
132. Zhang Y, Zhao H, Cao Z, Sun X, Zhang C, Cai W, et al. A randomized clinical trial of laparoscopic Roux-en-Y gastric bypass and sleeve gastrectomy for the treatment of morbid obesity in China: a 5-year outcome. Obes Surg 2014;24(10):1617-24. (In eng). DOI: 10.1007/s11695-014-1258-2.
133. Pullman JS, Plank LD, Nisbet S, Murphy R, Booth MWC. Seven-Year Results of a Randomized Trial Comparing Banded Roux-en-Y Gastric Bypass to Sleeve Gastrectomy for Type 2 Diabetes and Weight Loss. Obes Surg 2023;33(7):1989-1996. (In eng). DOI: 10.1007/s11695-023-06635-x.
134. Salminen P, Grönroos S, Helmiö M, Hurme S, Juuti A, Juusela R, et al. Effect of Laparoscopic Sleeve Gastrectomy vs Roux-en-Y Gastric Bypass on Weight Loss, Comorbidities, and Reflux at 10 Years in Adult Patients With Obesity: The SLEEVEPASS Randomized Clinical Trial. JAMA Surg 2022;157(8):656-666. (In eng). DOI: 10.1001/jamasurg.2022.2229.
135. Axer S, Al-Tai S, Ihle C, Alwan M, Hoffmann L. Perioperative Safety and 1-Year Outcomes of Single-Anastomosis Duodeno-Ileal Bypass (SADI) vs. Biliopancreatic Diversion with Duodenal Switch (BPD/DS): A Randomized Clinical Trial. Obes Surg 2024;34(9):3382-3389. (In eng). DOI: 10.1007/s11695-024-07421-z.
136. Talebpour M, Sadid D, Talebpour A, Sharifi A, Davari FV. Comparison of Short-Term Effectiveness and Postoperative Complications: Laparoscopic Gastric Plication vs Laparoscopic Sleeve Gastrectomy. Obes Surg 2018;28(4):996-1001. (In eng). DOI: 10.1007/s11695-017-2951-8.
137. Grubnik VV, Ospanov OB, Namaeva KA, Medvedev OV, Kresyun MS. Randomized controlled trial comparing laparoscopic greater curvature plication versus laparoscopic sleeve gastrectomy. Surg Endosc 2016;30(6):2186-91. (In eng). DOI: 10.1007/s00464-015-4373-9.
138. Spaggiari M, Di Cocco P, Tulla K, Kaylan KB, Masrur MA, Hassan C, et al. Simultaneous robotic kidney transplantation and bariatric surgery for morbidly obese patients with end-stage renal failure. Am J Transplant 2021;21(4):1525-1534. (In eng). DOI: 10.1111/ajt.16322.
